# Patterns of Predicted T-Cell Epitopes Associated with Antigenic Drift in Influenza H3N2 Hemagglutinin

**DOI:** 10.1371/journal.pone.0026711

**Published:** 2011-10-24

**Authors:** E. Jane Homan, Robert D. Bremel

**Affiliations:** ioGenetics LLC, Madison, Wisconsin, United States of America; Mayo Clinic, United States of America

## Abstract

Antigenic drift allowing escape from neutralizing antibodies is an important feature of transmission and survival of influenza viruses in host populations. Antigenic drift has been studied in particular detail for influenza A H3N2 and well defined antigenic clusters of this virus documented. We examine how host immunogenetics contributes to determination of the antibody spectrum, and hence the immune pressure bringing about antigenic drift. Using uTOPE™ bioinformatics analysis of predicted MHC binding, based on amino acid physical property principal components, we examined the binding affinity of all 9-mer and 15-mer peptides within the hemagglutinin 1 (HA1) of 447 H3N2 virus isolates to 35 MHC-I and 14 MHC-II alleles. We provide a comprehensive map of predicted MHC-I and MHC-II binding affinity for a broad array of HLA alleles for the H3N2 influenza HA1 protein. Each HLA allele exhibited a characteristic predicted binding pattern. Cluster analysis for each HLA allele shows that patterns based on predicted MHC binding mirror those described based on antibody binding. A single amino acid mutation or position displacement can result in a marked difference in MHC binding and hence potential T-helper function. We assessed the impact of individual amino acid changes in HA1 sequences between 10 virus isolates from 1968–2002, representative of antigenic clusters, to understand the changes in MHC binding over time. Gain and loss of predicted high affinity MHC-II binding sites with cluster transitions were documented. Predicted high affinity MHC-II binding sites were adjacent to antibody binding sites. We conclude that host MHC diversity may have a major determinant role in the antigenic drift of influenza A H3N2.

## Introduction

Influenza viruses cause a major burden of disease, and spread rapidly throughout global populations. Many factors contribute to the infectivity and transmissibility of influenza viruses. Among these are the presence of specific sialic acid receptors [1], the enzyme cleavage sites in hemagglutinin [2], peptide transporter processing [3], innate immune defenses [4], and the presence of neutralizing antibody [5]. The high degree of variability of the hemagglutinin protein subunit (HA1), to which neutralizing antibody binds, is well known. Antigenic drift allowing escape from neutralizing antibodies is an important feature of the continued transmission and survival of seasonal influenza viruses in populations from year to year [6], [7]. This makes the task of selecting vaccines an ongoing challenge [8].

Antigenic drift is attributed to selection under pressure of an immune response and has been measured primarily by escape from the neutralizing effect of antibodies [7]. Antigenic drift has been studied in particular detail for influenza A H3N2, which emerged first in epidemic form in 1968. Multiple specific amino acid changes in the HA1 protein associated with antigenic drift have been identified [9]–[14]. Smith *et al* [9] have mapped the effect of progressive genetic mutations leading to antigenic change, as detected by hemagglutination inhibition with polyclonal ferret antisera. This study showed well-defined antigenic clusters of influenza A H3N2 virus isolates emerging chronologically.

Antibody alone can neutralize influenza virus infectivity and prevent infection [15], [16]. In primary infection the antibody response is the result of both B-cell and T–cell stimulation and is polyclonal. The spectrum of antibodies present in a polyclonal response is a function of which B-cells and T-helper cells are stimulated. The neutralizing antibody response on re-exposure is the combination of neutralization by residual circulating antibody, anamnestic stimulation of a subset of both B-memory cells and T-memory cells, as well as *de novo* antibody formation. Unlike the molecular mechanism of neutralization of virus by antibody, the pathways of antibody production which involve function of T-cells are dependent on MHC binding of peptides and hence vary with host MHC allelic diversity. CD8+ cytotoxic T-cells (CTL) have been shown to have a role in limiting the duration of virus shedding and in eliminating virus infected cells [17], [18].

CD4+ cells are not effective at achieving viral clearance in the absence of B-cells; a T-dependent antibody response is a key component of the CD4+ role [16], [19]. CD4+ T-cell responses are also essential for a fully developed CD8+ T-cell response to influenza [20].

Screening studies using synthetic peptide probes have identified CD4+ T-cell epitopes broadly distributed in the HA and neuraminidase [21], [22]. Importantly, Barnett *et al* in 1989 [23] showed the common location of CD4+ epitopes and B-cell epitopes, primarily in the variable regions of HA1 in H3N2 influenza, and pointed to the possibility of a role of MHC polymorphism in antigenic drift. CD8+ CTL epitopes have been identified in most influenza A proteins [24]. Single amino acid sequence differences in H3N2 nucleoprotein peptides binding to MHC-I molecules have been shown experimentally to allow escape from recognition by cytotoxic lymphocytes [25]–[28].

In understanding the role of antigen presenting cells (APC) in influenza, considerable emphasis has been placed on MHC-I CD8+ epitopes [29]–[31]. The role of B-cells as APC in influenza has received less attention. While all APC within the lung were able to stimulate MHC-I restricted responses, B-cells were very inefficient compared to macrophages or dendritic cells in this role [30]. The inability to infect B-cells with influenza, attributed to the absence of NF-kB signaling [32], means they may not be exposed to influenza core proteins and so may have a different APC function for influenza than do dendritic cells and macrophages.

Given the role of T-helper cells in determining the antibody spectrum, and CTLs in curtailing virus shedding, understanding the influence of host immunogenetics is a key part of understanding the immunological pressure bringing about antigenic drift. Experimental studies of T-cell epitopes necessarily take a reductionist approach, in which individual interactions of single MHC alleles and specific virus peptides are examined.

In this study we use a bioinformatics approach to examine the interface of influenza virus diversity with host immunogenetics at a population level and to address the consequent variations in immunologic selection pressure.

We recently described a method, termed uTope™ analysis, for whole proteome mapping of predicted binding to MHC-I and MHC-II molecules. In this approach binding affinity is represented by the three dominant physical property principal components of each amino acid making up each peptide 9-mer or 15-mer, respectively [33], [34]. This method offers significant advantages over previous bioinformatics approaches, which depend on position specific matrices [35]. In the present study, we applied uTOPE™ analysis to ask how patterns of antigenic drift in influenza H3N2, as monitored by antibody binding over time, compared to the patterns of predicted T-cell epitopes reflected in predicted MHC binding within the HA1 of influenza H3N2. We examined the interaction of protein sequences for the HA1 of 447 H3N2 virus isolates with 35 MHC-I HLAs and 14 MHC-II alleles. We compared clusters based on predicted MHC binding patterns with those described by Smith *et al* [9]. We further examined the impact of individual changes in HA1 amino acid sequences between virus isolates representative of different antigenic clusters over time to understand the changes in MHC binding. By analyzing all possible MHC-peptide interactions within HA1 of several hundred virus isolates and for a large number of MHC alleles, sufficient data density is achieved to allow patterns of MHC binding to be appreciated and analyzed. We examined how host immunogenetics contributes to determination of the antibody spectrum and hence the immune pressure bringing about antigenic drift. We conclude that MHC diversity likely has a major determinant role in the antigenic drift of influenza A H3N2.

## Methods

### Database and Statistical Software

All mathematical operations, database operations, and statistical analysis were carried out with JMP® version 9.0 or JMP Genomics® version 5/SAS 9.2 using the JMP® scripting language [36]. The MegAlign® application within LaserGene® v8 was used for sequence alignments. Much of the work involved populations of numerical data standardized to zero mean and unit variance. The term σ is used throughout to describe the numerical data in standard deviation units.

### Workflow

**Figure 1:**
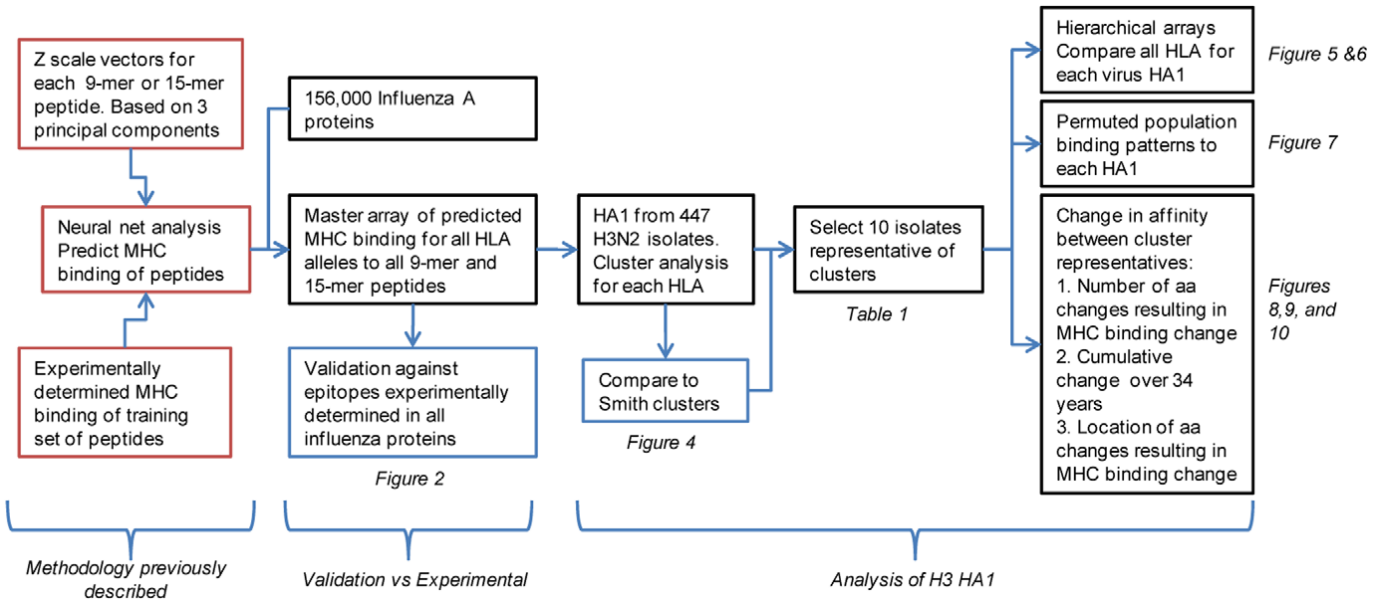
Workflow of bioinformatics analysis.

Figure 1 outlines the sequence of analytical steps applied. In particular it differentiates between those analyses conducted on all influenza proteins (validation), all H3N2 HA1 proteins (cluster analysis), and on a representative subset of H3N2 HA1 (detailed analysis on the impact of mutations).

### Viruses and Curation of Sequences

A set of approximately 156,000 influenza A proteins was assembled from the Genbank Influenza database in December 2010. HA1 proteins for all H3N2 isolates from 1968 to present were extracted. Some of the H3N2 viruses used by Smith *et al* [9] were not in the primary influenza database; these were located by Genbank searches and consolidated with the primary collection. Several quality control procedures were applied to the dataset prior to use. A small number of duplicate sequences with the same Virus ID but different Genbank accession numbers were removed. All amino acid position assignments used in this paper are based on the N-terminal methionine and include the signal peptide. As the clustering algorithms used are intolerant of missing amino acids, sequences in the database without a signal peptide were edited to add a consensus signal peptide. Sequence submitters have not used uniform C-termini for HA1; we terminated all HA1 at position 345 prior to cluster analysis. One sequence, A/Moscow/10/1999(H3N2)_49339009, had several amino acid deletions marked and was removed.

The resulting virus dataset comprised 260 HA1 used by Smith *et al* [9] which we designated by a prefix of the Smith cluster designation (HK68, EN72 etc.). An additional 187 H3N2 HA1 proteins from isolates dated 1968–2002 were included and given the prefix of the year of the isolate and “NON” (NON1998, NON2001 etc.).

For each Smith cluster a single representative virus isolate was selected for further analysis. The isolates selected are shown in Table 1. These were chosen from those which, on initial clustering analysis based on MHC binding patterns, were located in the mainstream of the Smith cluster groups. As our list only comprised two isolates from TX77 and in many instances these isolates clustered with EN72, no TX77 representative was selected for further comparisons.

**Table 1:** Influenza H3N2 virus isolates selected as cluster representatives.

Cluster	Representative virus isolate	GI Accession number for HA
HK68	Bilthoven/16190/68	49339049
EN72	England/42/1972	6470275
VI75	Bilthoven/1761/76	49338983
BK79	Netherlands/209/80	49339065
SI87	Victoria/7/87	2275517
BE89	Madrid/G12/91	49339129
BE92	Finland/247/1992	49339247
WU95	Wuhan/359/1995	49339351
SY97	Netherlands/427/98	49339385
FU02	Netherlands/22/03	49339039

### Epitope prediction methods

The uTOPE™ methods used to predict MHC binding affinity using a neural network prediction scheme based on amino acid physical property principal components have been described in detail elsewhere [33], [34]. Briefly, for MHC-II the protein was broken down into 15-mer peptides each offset by 1 amino acid. The peptide 15-mers were converted into vectors of principal components wherein each amino acid in a 15-mer is replaced by three z-scale descriptors. {z_1_(aa_1_),z_2_(aa_1_),z_3_(aa_1_)}, {z_1_(aa_2_),z_2_(aa_2_),z_3_(aa_2_)}, … {z_1_(aa_15_),z_2_(aa_15_),z_3_(aa_15_} that are effectively physical property proxy variables. With these descriptors, ensembles of neural network prediction equation sets were developed using publicly available datasets of peptide-MHC binding data wherein the inhibitory concentration 50% (ic_50_) has been catalogued as a measure of binding affinity of the peptides for a number of different HLAs [37], [38]. Because the ic_50_ data have a numerical range in excess of 10,000-fold, they were natural logarithm transformed to give the data better distributional properties for predictions, and subsequent statistical analysis used the log normal inhibitory concentration_50_ (ln(ic_50_)). For each of the 15-mers predicted ln(ic_50_) values were computed for fourteen different human MHC-II alleles: DRB1*01:01, DRB1*03:01, DRB1*04:01, DRB1*04:04, DRB1*04:05, DRB1*07:01, DRB1*08:02, DRB1*09:01, DRB1*11:01, DRB1*13:02, DRB1*15:01, DRB3*01:01, DRB4*01:01, DRB5*01:01. The peptide data was indexed to the N-terminal amino acid; thus each prediction corresponds to the 15-amino acid peptide downstream from the index position.

An identical process was then followed with all 9-mer peptides for prediction of binding to 35 MHC-I alleles: A*01:01, A*02:01, A*02:02, A*02:03, A*02:06, A*03:01, A*11:01, A*23:01, A*24:02, A*24:03, A*26:01, A*29:02, A*30:01, A*30:02, A*31:01, A*33:01, A*68:01, A*68:02, A*69:01, B*07:02, B*08:01, B*15:01, B*18:01, B*27:05, B*35:01, B*40:01, B*40:02, B*44:02, B*44:03, B*45:01, B*51:01, B*53:01, B*54:01, B*57:01, B*58:01.

Each of the alleles has a different characteristic mean and standard deviation of binding affinity to 9-mer and 15-mer peptides. Thus for statistical comparisons involving multiple HLA alleles, the predicted ln(ic_50_) values were standardized to zero mean and unit standard deviation on a within-protein basis. For the virus HA1 sequences the standardization of the affinity of the HLA binding predictions was done on a within-virus basis. For the validation test set, the grand mean and grand standard deviation of each HLA across all 10 influenza proteins from multiple influenza viruses represented in the dataset (124 different proteins in all), were used to compute the standardized binding affinity of the isolated peptides.

### Validation Dataset and Approach

To evaluate the validity of the epitope prediction method in the context of influenza virus, a dataset of T-cell epitopes experimentally defined by others in each of the proteins of influenza A viruses was prepared from records at the Immune Epitope Database (IEDB, iedb.org) and by reference to the source publications. From the available experimental data, a set of 460 non-redundant experimentally defined unique peptides was assembled. This included 296 experimentally determined epitope positive and 164 negative. Epitopes had been defined since 2000 using chromium release, ELISPOT, or MHC tetramer binding and the HLA restriction was known. Details of the selection process are included in Table S1, which includes a table of the epitopes used.

Using a set of 156,000 influenza A proteins, representing all influenza proteins, we generated a master array of the predicted binding affinity to HLA alleles for all 9-mer and 15-mer peptides for each protein. Those corresponding to the experimentally determined epitopes were compared. For this purpose the recursive partitioning platform of JMP using a 5 K-fold cross-validation was used. The area under the receiver operator characteristic curve (AROC) was used as a measure of the sensitivity and accuracy of the predictions. A confusion matrix was generated showing agreement/ disagreement (true positives, false positives, true negatives, false negatives) with the 460 experimental measurements. We have previously described the use of uTOPE™ analysis for B-cell epitope prediction [34]. This approach was applied to the cluster representative isolates.

### MHC binding predictions and analysis and clustering methods

All subsequent analyses were derived from a subset of the master array of individual MHC-peptide binding affinities of all influenza proteins described above, initially for 447 H3 HA1 and then for a representative subset of 10 HA1 proteins.

Cluster analysis was conducted to examine patterns of similarity and dissimilarity in MHC binding between virus isolates. For clustering of the virus protein sequences, the amino acid sequence of each isolate protein was represented by vectors of predicted ln(ic_50_) data sequentially indexed by 1 amino acid as described above. Two forms of data were used depending on the application. For certain analyses the ln(ic_50_) data were standardized by subtraction of the mean and division by the standard deviation to produce a uniform scale with a mean of zero and standard deviation of one, a process common in statistical analysis. In other analyses non-standardized ln(ic_50_) data were used. Clustering was done using the hierarchical and K-means clustering platforms of JMP®. These platforms have a wide range of tools to assess the quality of the analysis, and these were used as appropriate. The K-means platform was used initially to obtain an estimate of the optimal number of clusters within the dataset for each of the HLA alleles [39]. Using the 447 viruses in the data set with each of 35 MHC-I and 14 MHC-II alleles, the optimal number of clusters ranged from 17 to 23. As a compromise, the larger of these numbers was used for all subsequent work with hierarchical clustering where the number of clusters of interest can be manually specified for graphical and textual output. To order the clusters in the hierarchical clustering process, principal component analysis was first carried out on the dataset, and the order of the clusters was based on the first principal component of the dataset under consideration. Clusters of HA for the 447 viruses based on MHC binding were assembled; initially one by one for each HLA to compare to the Smith clusters. We then prepared hierarchical arrays which compared binding for each HLA to the HA1 of selected representatives of each cluster.

### Permuted population binding patterns

For certain analyses and graphical representations of MHC binding by the population, the permuted minimum algorithm described previously was used [34]. This is based on the concept that within a heterozygous or homozygous individual, the HLA allele providing the highest binding affinity to any specific peptide will prevail at each MHC locus. All possible heterozygous and homozygous HLA pair combinations at each allele were determined and the best affinity of the two selected. These values were then averaged across all possible combinatorial pairs of alleles available in the prediction data set to arrive at a prediction for each peptide for a metapopulation. Hence, for N = 14 MHC-II, 105 combinations are used (((N^2^−N)/2)+N). The MHC binding affinity to a peptide can change over several orders of magnitude for a single amino acid index position increment along the protein. As an MHC can bind in any of several different indexing positions, if one of the indexing positions of either of the heterozygous alleles provides for a high binding affinity then the peptide will effectively be trapped in that location. Thus, to compute the permuted phenotype, the highest binding affinity for each allelic pair within a narrow averaging window (±2–±4 amino acids) around the index amino acid position was recorded and then averaged over all potential combinatorial pairs in a population. For plotting the results we applied the minimal distortion polynomial filter of Savitsky and Golay [40].

### Changes in binding affinity

We developed algorithms and statistical metrics that enabled quantifying and visualizing changes in individual peptide-MHC binding arising from the amino acid changes between each pair of cluster-representative viruses stepwise in chronological order (HK68 to EN72…HK68 to FU02). Changes in predicted binding of a peptide, indexed to the N-terminus amino acid of the 9-mer or 15-mer, were scored on a 5-point basis as shown in Table 2. The sign and magnitude of the scoring parameter indicate the direction and size of the change in ln(ic_50_) value; hence a negative sign indicates a shift toward higher affinity. A −2 therefore indicates a large change in affinity with the result being a high affinity binder. This allowed us to analyze (1) how many amino acid mutations were needed to bring about a significant change in MHC binding, (2) to determine the aggregate of changes in the HA1 protein over time, and (3) to plot where within HA1 mutations leading to gain or loss of high affinity MHC binding were occurring.

**Table 2:** Classification of changes in predicted peptide HLA binding affinity due to amino acid changes.

Classification	High affinity binder status	Binding affinity before and after	Description of amino acid change impact
−2	New	>−1σ to ≤−1σ	Large increase in affinity (>1σ change) to surpass threshold and become a new high binder
−1	Retained	≤−1σ to ≤−1σ	Already a high binder, further affinity increase
0	No Change	≤−1σ to ≤−1σ	High affinity, no change
+1	Retained	≤−1σ to ≤−1σ	A high binder, affinity loss but remains above threshold as a high binder
+2	Lost	≤−1σ to > = 0	High affinity initially; large decrease to below high binder threshold

The impact on binding affinity associated with amino acid changes arising in cluster transition was determined. Based on impact of the mutations on predicted binding affinity a classification code was assigned to each peptide for each of the affected MHC molecules. The threshold of −1σ was selected based on the outcome of recursive partitioning (Figure S1). The peptides were then assigned to different one of five different classifications according to whether high affinity binding was gained, lost or retained above the threshold. Only high binders were considered, so some peptides that did not surpass the threshold were not included in the analysis of the particular transition.

## Results

### Validation of analysis

**Figure 2:**
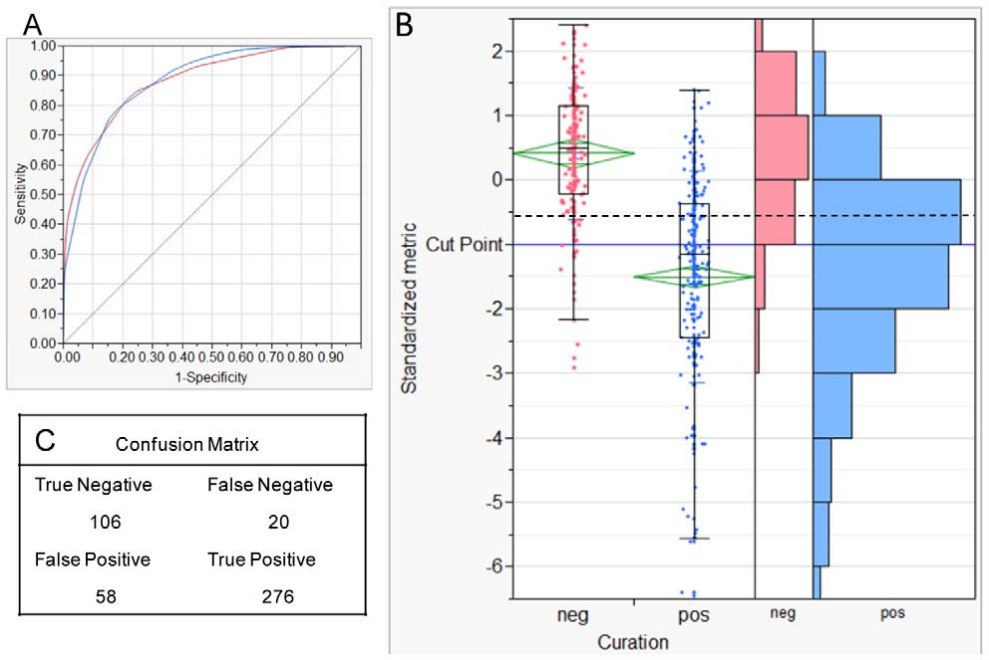
Relationship between MHC binding prediction and curated epitope characterizations. **Panel A**. The area under the ROC curve of 0.88 reflects the categorization reliability at the optimum cut-point of −0.53σ chosen by the recursive categorization process. **Panel B**. The chart shows that the mean of the positive class was approximately −1.5σ (center of the diamond). The mean of the negative class is approximately +0.4σ. The ends of the boxes are the 25th and 75th percentile and the horizontal line in the box is the median. The ends of the lines are the 5th and 95th percentiles of the respective distributions. Many points are plotted on top of each other but the histograms on the right shows the data distribution. A cut-point of −0.53σ was chosen by the recursive categorization process (dotted line). The cutpoint at −1σ (solid line) is a more rigorous threshold which was used as a readily understandable criterion throughout the study. **Panel C**. The confusion matrix documents agreement and disagreement between predictive and experimental methods.

A meta-analysis was carried out using experimentally defined epitopes from all influenza proteins compared to the MHC affinities predicted by the *in silico* approach. Results are shown in Figure 2 for T-cell epitopes. There are caveats to interpretation of this type of analysis because the source of the data is the result of several experimental techniques, each with different endpoints and carried out by numerous laboratories without inter-laboratory validation. An extensive international collaboration effort for assessment of T-cell epitopes in Type I diabetes [41] has shown cross-laboratory standardization to be critical to producing valid T-cell epitope results. Nevertheless, by pooling epitopes characterized over several years by many laboratories, we obtained a much broader comparison than would have been possible within our own laboratory. Experimentalists tend to focus on testing and reporting on epitope peptides that are positive. This characteristic has three consequences that potentially affect the statistical analysis: the overall mean of the dataset is less than the mean of the proteins from which the epitopes were selected, the number of positives documented experimentally is much larger than the negatives, and the same epitope is tested experimentally numerous times by different laboratories. With these points in mind, we carried out a comparison between our *in silico* predictions and the assay results combining all HLAs, peptides, and experimental methods into a single consolidated dataset after having eliminated redundant measurements on the same epitope. Figure S1, provides the same analysis with redundant measurements included. For the combined, non-redundant dataset, the AROC across all experimental methods (Cr release, ELISPOT, and MHC tetramers) was 0.89 with an optimal cutpoint of −0.53σ (below the overall mean) on a standardized binding scale for selection of a positive. Thus, a binding affinity of less than −0.53σ can be interpreted as being a prediction metric for a positive T-cell response. This does not mean that binding predictions >−0.53σ might not be associated with a positive cellular response, but they cannot be reliably predicted as such by our neural net method. At this cutpoint, our predictions for the 460 experimentally characterized epitope were in agreement for 382 (83%) of the epitopes. For the remaining 17%, we cannot be sure whether our prediction or the experimental determination was the correct one; we note that in some cases experimental determination by two different methods also gave conflicting outcomes. Between the cutpoint and the overall mean of the dataset there is considerable overlap between the predictions for the positive and negative group. Both negative and positive categories span both sides of the mean with the negatives averaging about +0.4 σ and the positives average about −1.4 σ. As the binding data was normally distributed, we chose to use a somewhat more conservative cut point of −1σ of standardized binding data in the remainder of the study. In a recent study by Greenbaum *et al* [38] the 20^th^ percentile was chosen as a cutpoint for inclusion of data for a clustering analysis of MHC-II binding to a dataset of peptides, quite close to the −1σ value used here. A more detailed discussion and an analysis showing the effects of epitope selection bias and measurement redundancy is provided in Figure S1. The comparison of B-cell epitope predictions and curated epitope characterizations, less central to this study, are shown in Figure S2.

### Observations of binding patterns in the master array

**Figure 3:**
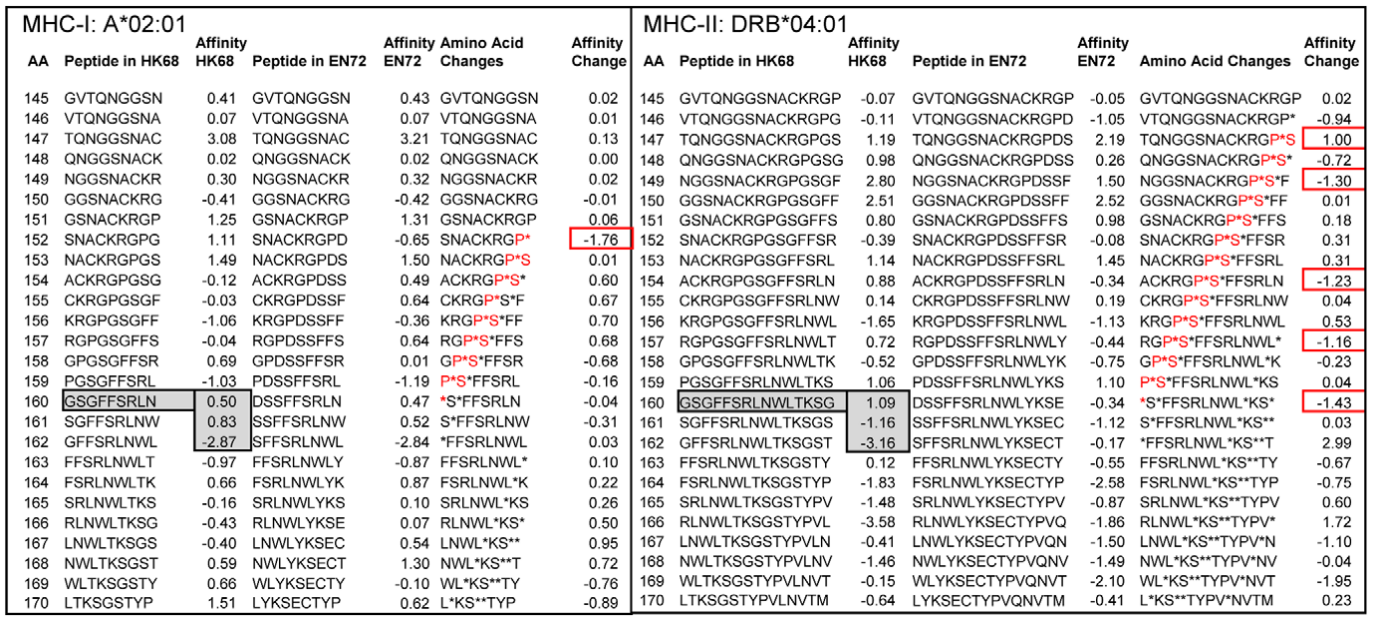
Impact of single amino acid changes on MHC-I and MHC-II binding. The examples of MHC-I A*02:01 and DRB1*04:01 binding to peptides in a section of HA1 from two isolates representing clusters HK68 and EN72 (isolates as shown in Table 1) show (a) that a single amino acid displacement can significantly impact predicted binding affinity and (b) that a single amino acid change impacts predicted MHC binding across multiple registers. For both A*02:01 and DRB1*04:01 the predicted binding affinity of the peptide starting at index position 160 is significantly different from that in either of the two positions starting at 161 or 162. A change in the amino acid at position 160 may contribute to the appearance of a new MHC-1 A*02:01 high affinity binding site with index position 152; it may also contribute to new high affinity MHC-II DRB1*04:01 binding peptides with index positions at 160, 157, 154, and 149 and the loss of a high affinity binding peptide with index at position 147. Standardized binding affinity is shown as standard deviations below the mean (σ). Position numbering is from the signal peptide N terminus.

As an illustration of the binding prediction array output, Figure 3 shows binding affinity for two HLAs, DRB1*04:01 and A*02:01, for a small subset of sequential peptides from two virus isolates (HK68 A/Bilthoven/16190/68 and EN72 A/England/42/1972), as well as the difference in binding affinity between the isolates at each position. The binding affinity varies greatly with a single amino acid displacement along the protein. As shown in Figure 3 for the HK68 isolate, a DRB1*04:01 MHC-II binding a 15-mer with N terminus at 160 (ln(ic_50_) = +1.09 σ) would have a very different outcome from one which binds one or two amino acids downstream (ln(ic_50_) = −1.16 σ and −3.16 σ). Likewise, single amino acid substitutions can result in a significant change in predicted MHC binding affinity (see right hand side of each panel in Figure 3). In assessing the effect of an amino acid mutation on MHC binding to its impact on antibody binding, it is important to recognize the multiple registers of MHC binding that may be affected by a single amino acid. Although conceptualized and treated as specific 15-mers with a core 9-mer binding domain, the large entropy contribution implies a more dynamic association of the HLA with the peptide [41], where the peptide might adopt several energetically equivalent binding registers. Further, the effect of an amino acid change is not necessarily, or only, when it occurs at the index position of a peptide, but rather may extend upstream to all 15-mer or 9-mer registers in which it participates. For example, substitution of glycine by aspartate at position 160 between the HK68 isolate and the EN72 isolate brings about changes of >1σ in binding affinity in multiple registers upstream for both the alleles shown.

### Cluster analysis of H3N2 HA1 based on predicted MHC binding patterns over time

The array of predicted binding affinities of 345 successive peptides for each of the 447 HA1 was clustered, based on the patterns of binding affinity for each one of the 35 MHC-I and 14 MHC-II alleles. These are very large arrays comprising over 154,000 datapoints. Dendrograms were drawn of the clustering patterns for each MHC allele. Two representative dendrograms are shown in Figures S3 and S4, these are for the widely studied HLAs A*02:01 and DRB1*04:01. These large figures can be zoomed to allow the list of viruses to be reviewed. Observation of the dendrograms shows that MHC-I binding patterns were more conserved than MHC-II binding patterns. Low binding affinity regions of the protein remained unchanged, in some cases through 34 years. Based on the K-means clustering algorithm the 447 viruses were grouped into 23 clusters. Although in the present analysis there are more clusters than were found by Smith *et al* [9], this difference is largely due to additional metrics used in the present study. Despite the larger number, clustering based on MHC binding closely mirrors that identified by Smith *et al* based on antibody hemagglutination inhibition studies and shows clearly defined clusters following a chronological progression.

**Figure 4:**
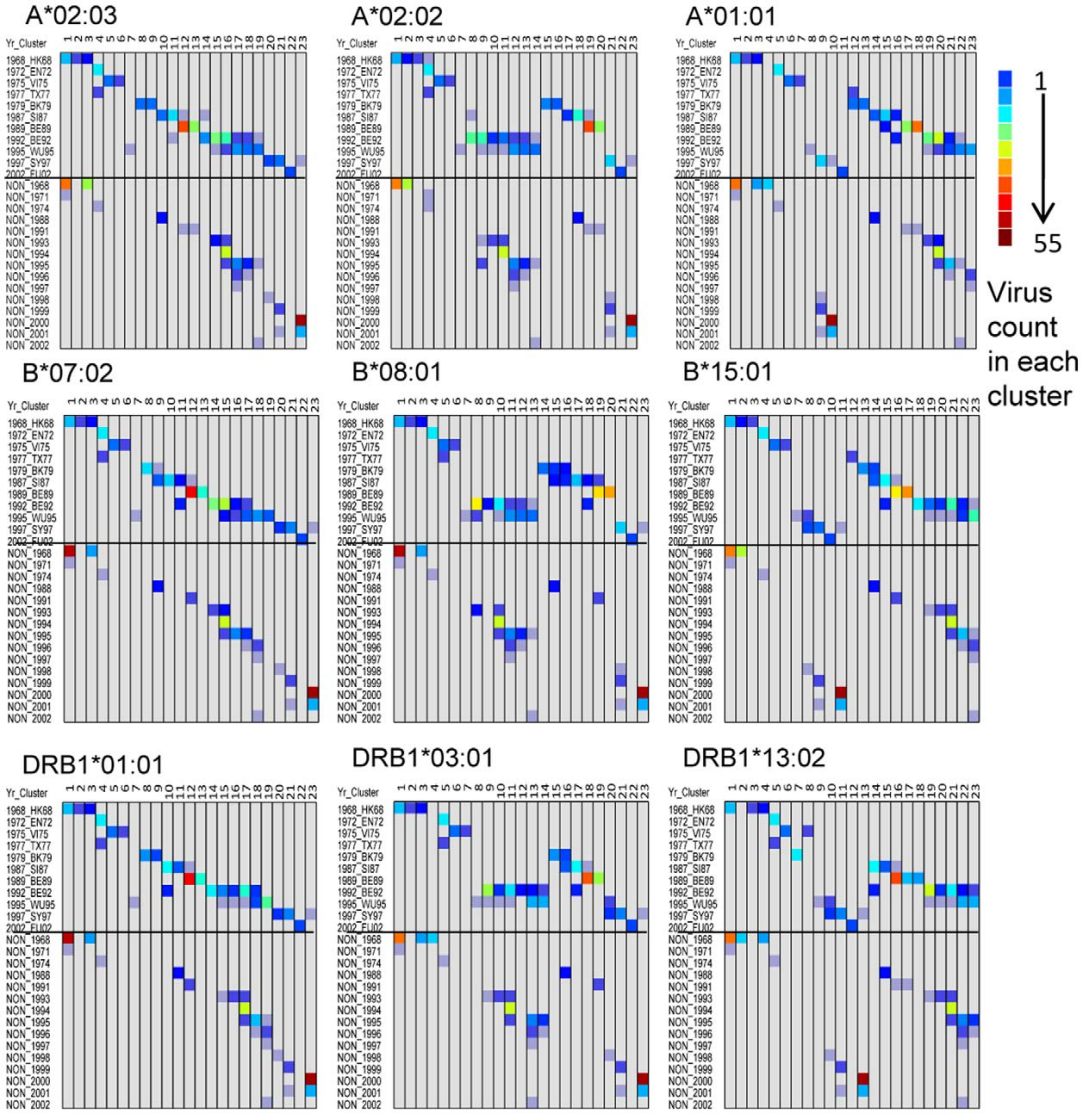
Representative cell plot patterns of the clustering of virus isolates based on predicted MHC binding patterns of HA1 compared to clusters defined by antibody binding. Hierarchical clustering of affinity patterns in HA1 of 447 virus isolates was carried out for each HLA as shown in Figures S3 and S4 using the Ward method. Each cluster was assigned a numerical ID from 1 to 23, shown in the X-axis. Cross-tabulations were then prepared that tallied the cluster numerical ID with the clusters identified by Smith *et al* [9] and shown in the upper half of the y-axis. The lower half of each plot shows the clustering of contemporaneous virus isolates that were not evaluated by Smith *et al*. arrayed against the year of isolation in the y-axis. The color scale shows the count of viruses in each cell. Each HLA generated a similar pattern but with different counts. Three broad types of pattern were noted and representative examples are shown here for MHC-I A and B and MHC-II DRB.

To facilitate comparisons between HLA alleles of the clustering patterns seen in the large dendrograms, cell plots of contingency tables were assembled. For each HLA allele these plots count the number of isolates in each cluster. Representative examples are shown in Figure 4. Isolates from each Smith cluster group are present in 1–4 contiguous clusters based on MHC binding. A few exceptions are noted. For example, in the case of A*02:01, the Smith BE92 cluster, which comprises 57 virus isolates, spans 7 clusters. Three WU95 isolates (A/Madrid/G252/93(H3N2))_49339273, A/Netherlands/399/93(H3N2))_49339305, and A/Netherlands/372/93(H3N2))_49339297 ) cluster with BE92; notably these are isolates which Smith found to be interdigitated with BE92.

Each HLA allele generates a slightly different contingency plot indicative of different clustering patterns. However, the plot patterns fall into three related groups within each of MHC-I A, MHC-I B and DRB1, examples of which are in Figure 4. For each HLA, isolates comprising each Smith cluster tend to locate together, but in a different relative order. NON isolates (the contemporaneous virus set not studied by Smith *et al*) are arrayed below the Smith cluster isolates in each cell plot and form an approximately parallel pattern by date order for each HLA.

### *Selection of cluster representative viruses*

In light of the observation that each HLA generated a different cluster pattern, we selected a single representative virus from each of ten Smith clusters and examined their interaction with the different MHC alleles more closely. These viruses are listed in Table 1.

### Hierarchical clustering shows diverse binding by HLA

**Figure 5:**
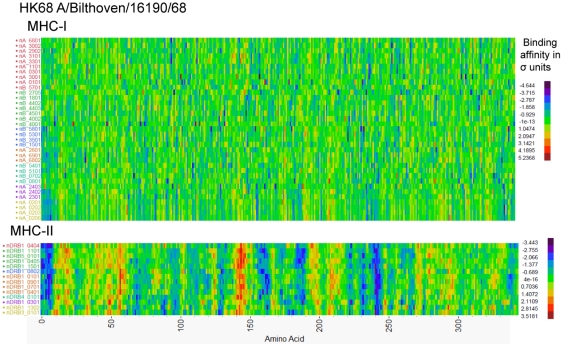
Hierarchical Cluster of predicted MHC-I and MHC-II binding to HA1 from a HK68 cluster representative isolate. Hierarchical clustering of predicted binding of multiple MHC-I and MHC-II alleles to consecutive peptides in HA1 of Influenza A/Bilthoven/16190/68 was done by the method of Ward using Johnson Sb distribution standardized binding data [42]. The Y-axis lists the MHC alleles. The X-axis shows the protein amino acid positions N-C. Each pixel represents the 9-mer or 15-mer peptide which starts at that amino acid position. The binding data for each HLA was first submitted to principal component analysis and the first principal component was used as an ordering variable. In each case clusters are indicated by the color of the y-axis legend. Comparable plots of binding to HA1 of other cluster representative viruses are shown in Figure S5. The color scale shows the binding affinity as ln(ic_50_). Purple-blue colors indicate strong binders and red-orange pixels are weak binders.

The peptide binding affinity arrays for all peptides in the 10 representative HA1 were clustered based on similarity of the binding affinity patterns for each HLA. Within-virus standardization of each HLA allele prediction was done using the Johnson Sb distribution, which is most appropriate for measurements with experimental data having upper and lower bounds [42]. The output for one virus (HK68 A/Bilthoven/16190/68) is shown in Figure 5. Arrays for the other nine viruses are in Figure S5. In these plots the peptide binding patterns for all HLAs can be compared.

In general, more variation in binding affinity pattern is seen between MHC-II alleles than between MHC-I alleles. It should be emphasized that in the convention we have adopted, the coloration indicating affinity of binding the N-terminus of a 9-mer or 15-mer. When the arrays for each of the virus isolates are compared, the patterns across the protein are clearly visible but they differ, sometimes greatly, between HLA alleles at any single peptide. Marked differences in binding affinity can arise from a single amino acid displacement (see Figure 3). In these plots it is also apparent that certain regions of the molecule, for example peptides initiating at amino acids 155–190 and 220–250, have a much higher frequency of predicted high affinity binders (purple-blue coloration). Other regions, for example amino acids 40–60, have a preponderance of low affinity predictions (yellow–red).

**Figure 6:**
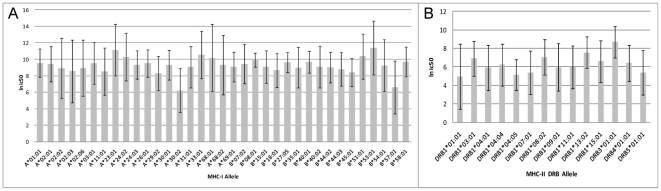
Comparison of mean and variation of binding affinity between HLA. Hierarchical clustering was performed as shown in Figure 3 for all cluster representative viruses shown in Table 1. **A.** shows the grand mean and standard deviation of predicted MHC-I binding affinity over all peptides in HA1 for the 10 representative viruses by allele. **B.** shows the same data for MHC-II.

When the ranking of HLA based on predicted MHC binding pattern is compared between each of the ten representative viruses, differences in rank order were noted. Figure 6 shows the variation in mean and standard deviation of predictions between HLA alleles. While some alleles were consistently high affinity binders (A*30:02, B*57:01, DRB1*01:01) and some were consistently low affinity binders (A*23:01, B*53:01, DRB3*01:01), others ranked differently for each virus. Again more variation in rank order and affinity was noted for MHC-II alleles than MHC-I.

### Permuted population binding patterns

**Figure 7:**
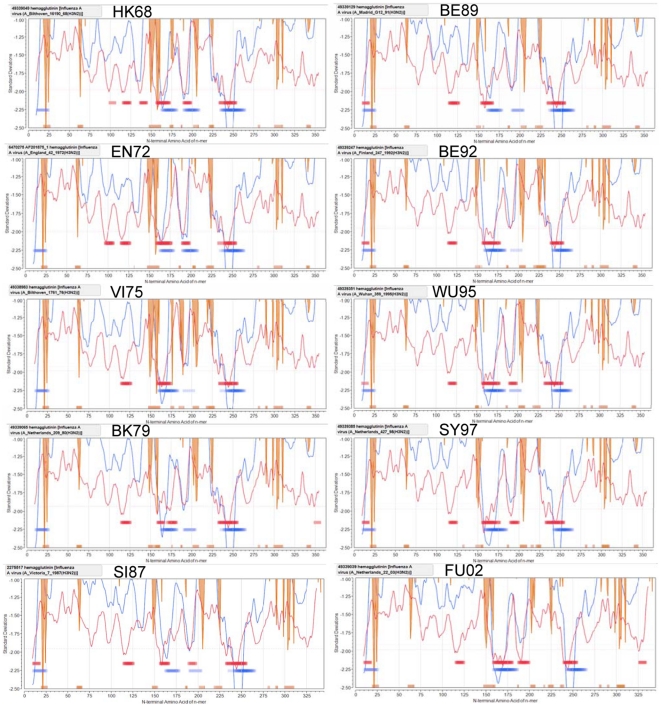
Permuted population plots showing predicted MHC-I and MHC-II binding and B-cell epitope sequences in the HA1 of ten selected viruses. The MHC-I (red line) and MHC-II (blue line) binding data are the permuted average binding for all combinations of alleles of each used in this study. Amino acid principal component neural net prediction of B-cell epitope sequences (orange vertical lines) are based on a neural net trained by BepiPred 1.0 output (not permuted). The horizontal red and blue bands are respectively the top 10th percentile of the permuted MHC- I and MHC-II predicted binding affinity. The orange bands are the top 25th percentile of B-cell epitope sequence predictions. In each case the line or band marks the index N terminal position of the 9-mer or 15-mer peptide for which it provides the data.

The cluster analyses have considered each HLA allele in isolation. However, in a population of individual hosts it is further necessary to consider the way in which a heterozygote carrying different combinations of alleles might respond. As described previously [34], we have found that a relatively simple combinatorial calculation for a heterozygous population captures some of the qualitative patterns noted above by the clustering graphics. For each of the ten representative viruses, graphical representations of the MHC binding affinity for the permuted population are shown in Figure 7. The regions with high and low binding for many alleles, noted in the cluster graphics above clearly stand out in these plots. The population plots bring together data for multiple immunological characteristics (predicted MHC-I binding as the red line, predicted MHC-II binding as the blue line, B-cell epitope probability by orange lines) for a metapopulation. They identify sequences which contain the highest probability, based on HLA genotype-pairs, of MHC-I and MHC-II high binding affinity (10^th^ percentile of minimum trace shown in red and blue ribbons, respectively) and high probability B-cell binding (orange ribbons show 25^th^ percentile Bayesian probability). We have shown that this type of plot correlates closely with experimentally defined epitopes in a very wide range of proteins [34]. Notably there is overlap of many MHC-I and MHC-II high probability binding sequences, and likewise overlap or proximity of MHC binding regions to B-cell binding. Each of the lines in these graphs are the running affinity averages of a very large number of combinatorial binding data (105 HLA pairs for MHC-II and 630 pairs for MHC-I). The range and pattern of changes of each of these averages is very characteristic for each protein. When the ten viruses are compared in Figure 7, subtle differences are seen in the trace of the average MHC-I and MHC-II binding (red line and blue line, respectively) over time. The plots clearly show minima (high affinity MHC binding) in areas that have been identified as being highly mutable and close to antibody binding sites [6], [9], [14] and that are visible as qualitative patterns in the cluster graphics (Figure 5). The orange shading marks predicted B-cell epitope sequences. These are consistent with the antibody binding regions mapped by Wiley at al [6]. Once adjusted for the 16 amino acid signal peptide, Wiley's Site A is at 138–162, Site B 171–213, Site C 66, 69–70, Site D 217–238, and Site E 276 and 278.

### Gain and loss of high affinity binding sites between cluster representatives

Based on the apparent coincidence between the areas of high affinity binding predictions and high mutability, the temporal changes in MHC binding over the entire HA1 molecule were examined by comparing the transitions between representative viruses.

### Amino acid mutations affecting MHC binding

**Figure 8:**
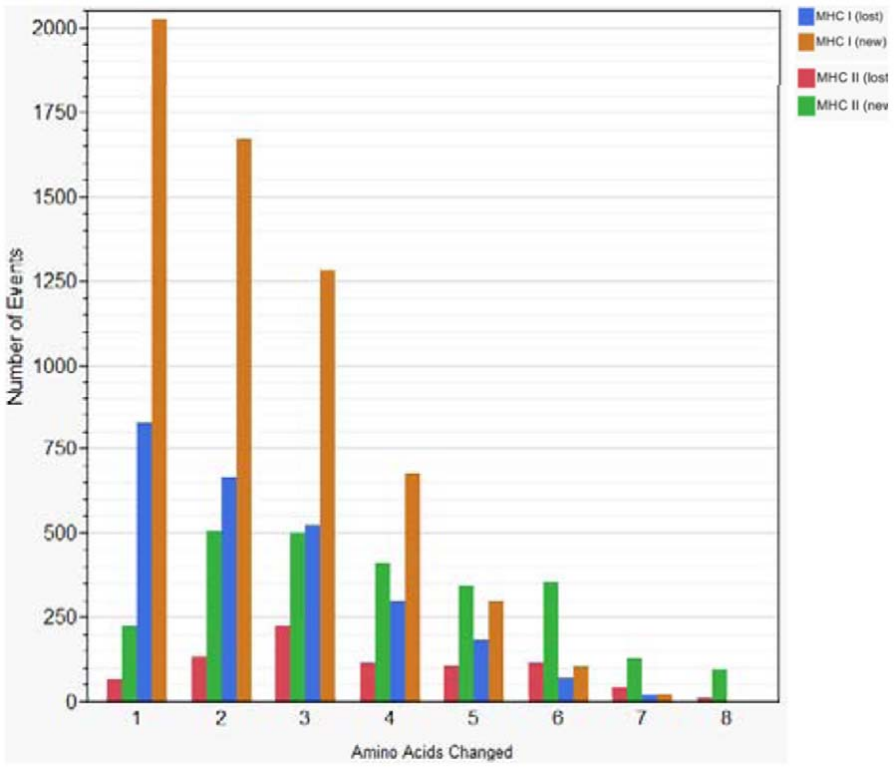
Number of amino acid mutations associated with the appearance or loss of high affinity binding peptides. The number of amino acid substitution events in 9-mer and 15-mer peptides from HA1 of the 447 viruses which result in appearance or loss of predicted high affinity binding of MHC-I and MHC-II respectively. Criteria for loss or gain are shown in Table 2. MHC-I loss: blue; MHC-I gain: tan; MHC-II loss: red; MHC-II gain: green.

Figure 8 shows that predicted MHC-II binding affinity changes and consequently epitope gain or loss occurred when 1 to 8 amino acid changes happened within a 15-mer peptide, however most changes resulted from 1, 2 or 3 amino acids mutations. For the MHC-II predictions it appears that the highest probability for generating a new high affinity binder is either 2 or 3 simultaneous mutations, whereas a single amino acid change can have dramatic effects on MHC-I binding. A striking feature is that simultaneous changes in several amino acids within either a 9-mer or 15-mer can give rise to effectively quantum changes in binding affinity. Thus a small number of simultaneous amino acid mutations triggering a change in MHC-II binding affinity could give rise to a change in the spectrum of epitopes presented to T-helper cells. An example of the data set showing the changes is provided in Table S2.

### Cumulative change in MHC binding

**Figure 9:**
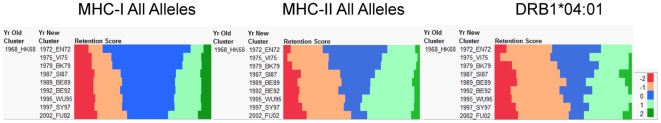
Predicted high affinity MHC binding peptides gained, lost and conserved in HA1 in transitions between temporal clusters of H3N2. For cluster representative viruses shown in Table 1, the scoring system shown in Table 2 was used to assign a categorical classification to predicted high affinity peptides according to whether they were new (−2), showed enhanced binding of an existing high binder (−1), conserved (0), showed reduced binding but were still high affinity (+1), or lost their status as predicted high affinity binders (+2). The plots show the aggregate number of each class of change in the HA1 protein associated with the transitions between clusters (HK68 to EN72, HK68-FU02 etc). Panel A shows the aggregate for all MHC-I alleles studied; Panel B shows the aggregate for all MHC-II alleles shown; Panel C shows DRB1*04:01 as an example of a single allele, other MHC-II alleles are shown in Figure S6.

We noted that there were regions of the HA1 molecule within which relatively high (−1σ) binding of peptides was retained over the entire period from 1968–2002. Other regions of the molecule showed very rapid changes in HLA binding characteristics. We therefore developed a system that enabled the visualization of temporal changes in binding behavior for all HLAs over the entire HA1 sequence over time. Figure 9 shows the aggregate change in MHC binding peptides for all peptides in HA1 at each cluster transition, as represented by the subset of ten viruses for all MHC-I and MHC-II alleles (Figure 9a and 9b). Figure 9c shows the aggregate changes for DRB1*04:01 as one example of the change in a single allele. Figure S6 provides the corresponding plots for other DRB alleles, each of which differs in the rate of change. Using the cluster transition of HK68 to EN72 as an example, during the ∼4 year period the majority of the HLA binding peptides were unchanged, but a few new high binders appeared and a few were lost. Comparing the changes across 34 years from HK68 to FU02 there is an accumulation of new high affinity binders. Even after this time a few high affinity peptides (<−1σ) are still retained, but most have been replaced. The patterns are similar for each of the successive transitional pairings. While the overall pattern is obvious, there are some HLAs in which practically all of the high affinity binders are lost over the 34 year period. On an individual allele basis very few high affinity MHC binding sites are retained intact through all the cluster transitions. It should be noted that the signal peptide (16 aa) was excluded from these plots as it was conserved (and in some cases inserted for alignment).

### Position of changes in MHC binding within HA1

**Figure 10:**
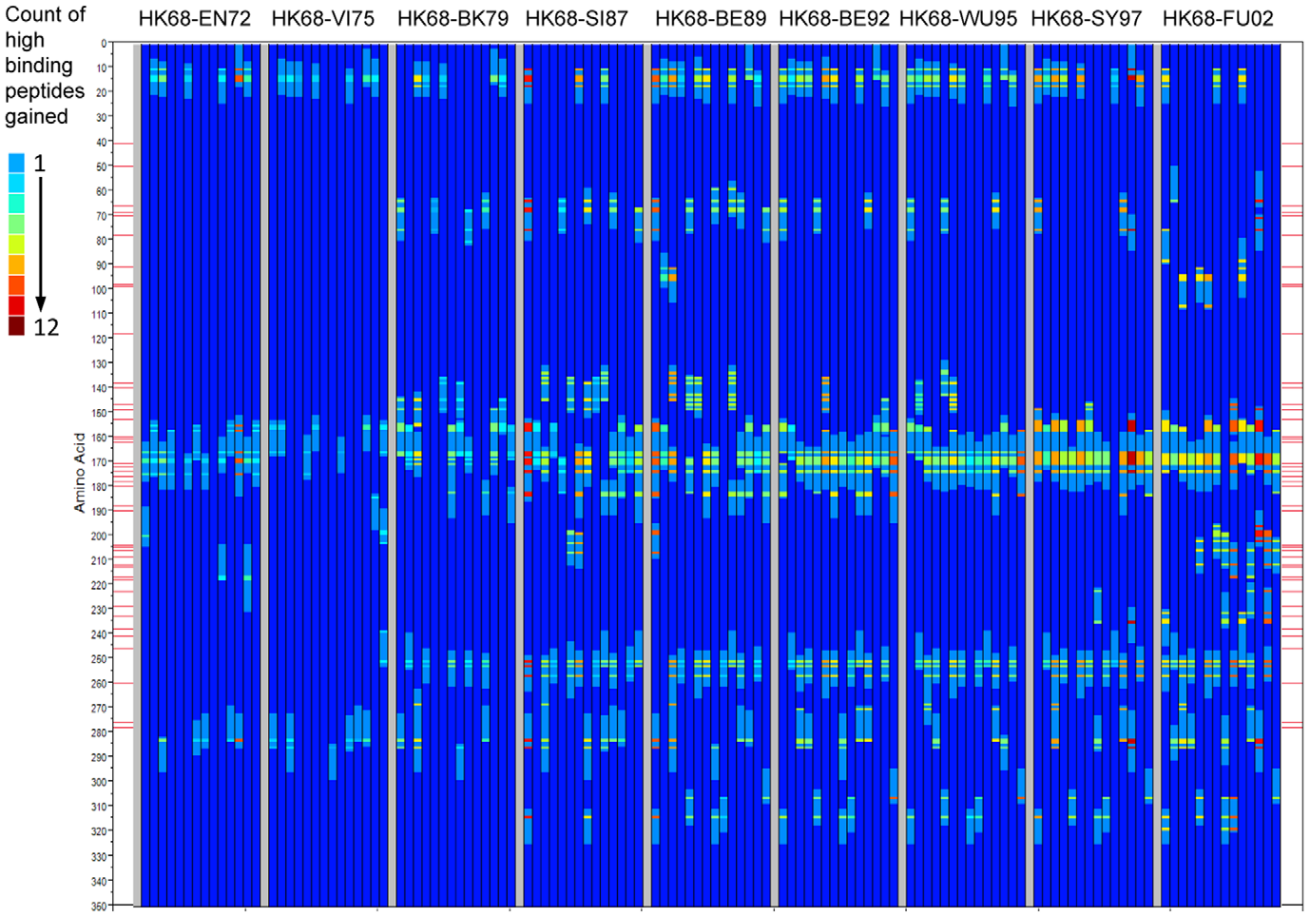
Position of peptides in which high affinity MHC-II binding is gained in cluster transition. The categorical classification of peptides according to whether they gained or lost high affinity binder status (Table 2) as a result of the transition between two cluster representative viruses (Table 1) was plotted across all HA1 amino acid positions for 14 MHC-II's. Each panel of nine lanes shows the changes in binding for the cluster transition indicated at the top of the panel (HK68-EN72 etc). Each of the lanes within the panel is the change in predicted binding affinity for a different MHC-II allele, left to right DRB1*01:01, DRB1*03:01, DRB1*04:01, DRB1*04:04, DRB1*04:05, DRB1*07:01, DRB1*08:02, DRB1*09:01, DRB1*11:01, DRB1*13:02, DRB1*15:01, DRB3*01:01, DRB4*01:01, DRB5*01:01. The red lines on each side are amino acid positions identified by Smith *et al* [9] as key mutation points for antigenic drift, these are taken from Smith's Table I and corrected for the presence of signal peptides in our numbering convention. This Figure shows the positions of predicted new high affinity binding peptides gained in the cluster transition. The color scale shown in the scale at left is the count of amino acid changes at a given position for all HLAs which result in the category of change mapped. Figure S7 shows intermediate changes in high affinity binding (−1 and +1 categories).

**Figure 11:**
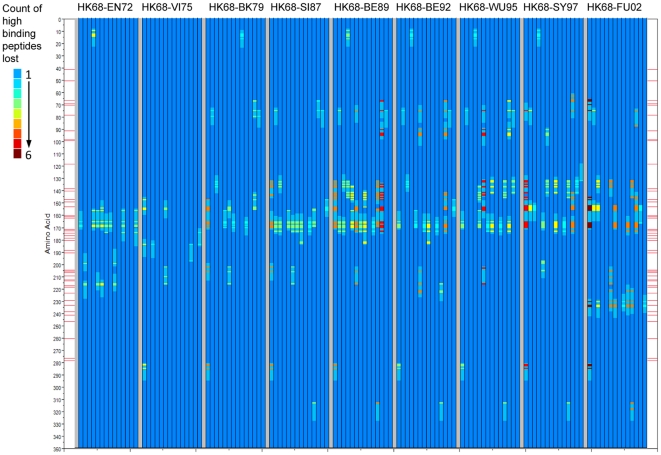
Position of peptides in which high affinity MHC-II binding is lost in cluster transition. Axes and lanes as described in Figure 10. The color scale shown in the scale at left is the count of amino acid changes at a given position for all HLAs which result in the category of change mapped.

**Figure 12:**
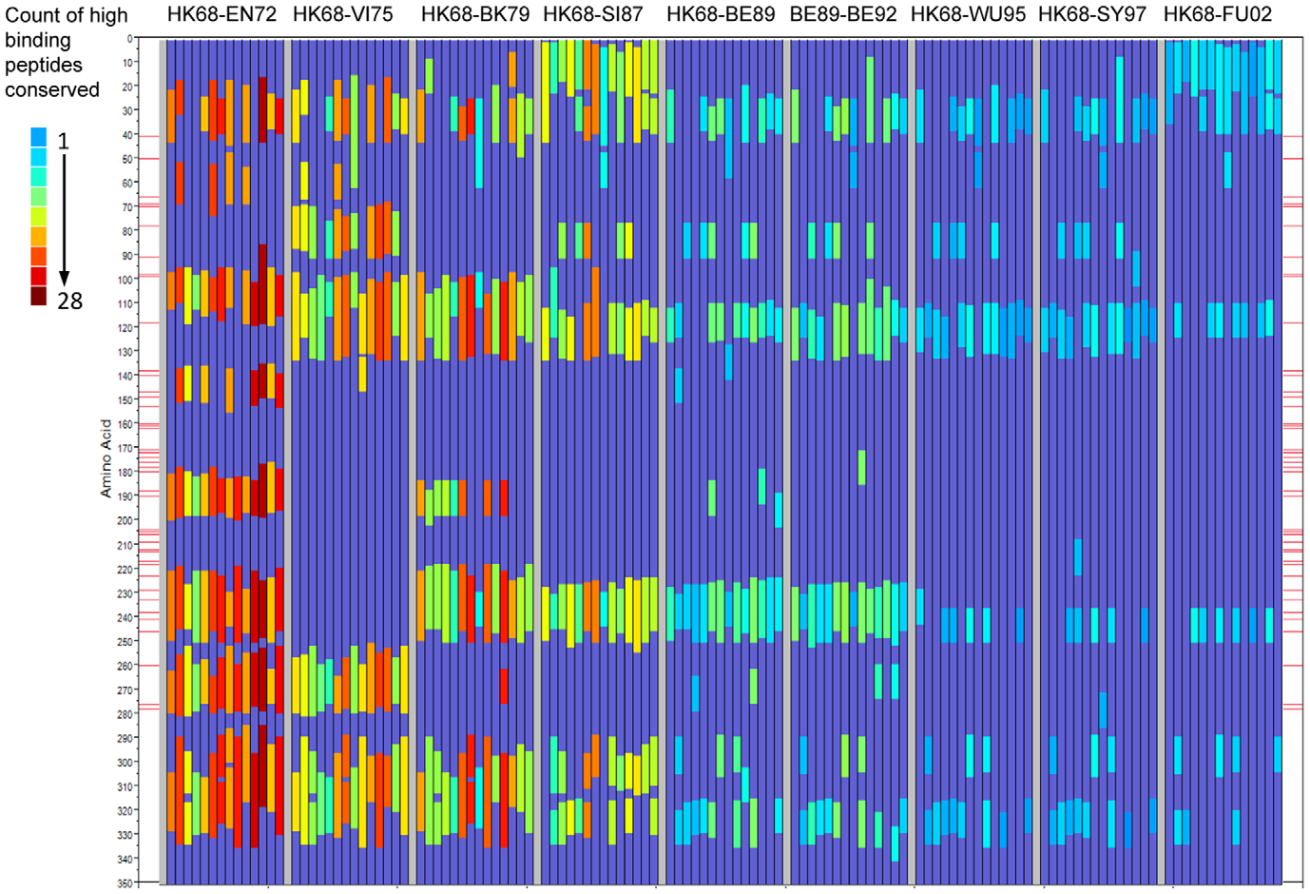
Position of peptides in which high affinity MHC-II binding is conserved in cluster transition. Axes and lanes as described in Figure 10. The color scale shown in the scale at left is the count of amino acid changes at a given position for all HLAs which result in the category of change mapped.

As Figure 9 considers the entire HA1 molecule, it is also of interest to know where in the sequence the change (or conservation) is occurring. Therefore we next constructed a graphic to show the locations within the HA1 proteins of peptides where binding affinity changes occurred between representative virus isolates. Figure 10 shows the cumulative gain in predicted high binding peptides across the nine cluster transitions for each of 14 MHC-II alleles. Figure 11 shows conversely the predicted high binding affinity lost by each allele over the same transitions. Figure 12 maps the high MHC binding affinity sites retained. Most addition and loss of high affinity MHC binding is seen in those peptides with index positions between amino acids 150–180 and 245–290. This places the highest probability of MHC-II binding change adjacent to or overlapping with neutralizing B-cell epitopes [6]. It is interesting that, in many cases, amino acids identified by Smith as essential to cluster transitional changes (and marked as red lateral lines in these figures) would be members of these 15-mer peptide. Once again we note the differences between individual MHC-II alleles. Figures 10a and 10b only represent the highest affinity binding peptide losses and gains (categories −2 and +2 in Table 2). Losses and gains of binding sites with less change in affinity (categories −1 and +1 in Table 2) are provided in Figures S7a and S7b but follow broadly similar patterns.

## Discussion

Our analysis provides the first comprehensive map of predicted MHC-I and MHC-II binding affinity for a broad array of HLA alleles for a complete influenza protein. We have analyzed binding for all 9-mer and 15-mer candidate MHC-I and MHC-II binding peptides in the HA1 protein of 447 H3N2 influenza virus isolates representing a span of 34 years, during which the changing pattern of antibody reactivity is known. This approach allows us to examine how a large group of viruses exhibiting antigenic drift may interact with an immunogenetically diverse host population.

The correlation of the experimental T-cell epitope definitions in the validation set with our predicted MHC binding affinity indicates that the *in silico* approach we have adopted is a valid surrogate for *in vitro* or *in vivo* experimental assays. uTOPE™ prediction provides a means of large scale analysis of potential host interface with hundreds of virus isolates. This allows a higher level view than can be derived from experimental data, which necessarily address a limited set of circumstances of virus isolate and HLA. *In silico* prediction provides a rapid and cost effective screening tool and guide to planning experimental design. As a single amino acid displacement can fundamentally change binding affinity of a peptide, it is essential to examine every candidate 9-mer or 15-mer peptide to appreciate the distribution of MHC binding sites across a protein. Many experimental efforts to determine T-cell epitopes have been undercut by the economics of peptide synthesis, which have dictated use of insufficient peptides to examine all possible sequential peptides. Our predictions suggest this pragmatic approach has likely caused many potential epitopes to be missed. The arrays used for the visualization and analysis shown here correspond to over 7 million individual peptide-MHC interactions, beyond the scope of the laboratory bench. When viewed on this large scale, pattern recognition is possible across the whole protein as well as between HLA alleles.

We have shown that the clustering of H3N2 viruses based on predicted MHC binding patterns within HA1 follows closely that described by Smith *et al* from analysis of antibody binding patterns [9]. Predicted MHC binding at any given peptide differs between HLA alleles. Single amino acid mutations or displacements can provoke dramatic differences in MHC binding. Similarly, simultaneous changes in several amino acids within a 15-mer or 9-mer can give rise to the appearance or loss of potential T-cell epitopes. We observed most variability in predicted MHC binding in positions adjacent to neutralizing antibody binding sites.

Several steps occur between the uptake of influenza virus or protein by an APC and the presentation of peptides by MHC molecules as potential T-cell epitopes. Variables such as antigen dose, peptide:MHC affinity, and the cytokine environment can all have an effect on the process [43]–[45]. Nevertheless, MHC binding is an absolute prerequisite and the stability of the peptide MHC complex plays an important role in determining T-cell clonal selection [43]. Hence, predictions based on affinity of MHC-peptide binding are a good starting point in T-cell epitope identification, whether evaluated *in silico* or by tetramer binding.

Proteasomal cleavage likely has a uniform and relatively random effect on all proteins. While each protein will have its own characteristic unfolding during the degradation process, peptides with higher affinity binding to MHC simply have a greater competitive advantage for MHC capture and presentation. Prediction tools (NetChop at CBS, cbs.dtu.dk/services/NetChop/) can be used to gain an assessment of the impacts of proteasomal processing on the presentation of peptides to bind to an MHC-I. The proteolytic activity of both the immunoproteasome and constitutive proteasome is quite aggressive. Indeed, several trials using recommended settings for the NetChop predictors show high probability of cleavage every 3–5 amino acids. There are no reliable predictors of MHC-II peptide production in APC.

T-cell responses have been reported to many epitopes in hemagglutinin in natural and experimental infections [21], [24], [27], [46]. HA peptide epitopes which elicit CD4+ T-cell responses have been shown to provide partial protection when used to vaccinate mice [47]. T-cell epitopes were shown to be located in the antigen binding sites of HA and shown to be affected by amino acid changes that occur during antigenic drift [23]. The most recent comprehensive study of CD4+ T-cell epitopes in hemagglutinin of H1N1 viruses by Richards *et al* [22], using naive DR1 transgenic mice and overlapping peptides, demonstrated that CD4+ T-cell epitopes are far more widely distributed in HA1 than previously appreciated.

That MHC binding patterns track antibody-detected antigenic change is, on one hand, not surprising; both are derivates of nucleotide sequences and mutation therein. As shown in Figures 10,11,12, many of the key amino acid positions important to antibody-detected cluster transitions can also affect MHC binding affinity of 15-mer peptides in multiple registers. On the other hand, the close relationship between antibody-detected antigenic drift and change in predicted MHC binding suggests a more functional relationship. T-cell responses to high affinity binding peptides which overlap, or are located close to, B-cell epitopes in the same protein may determine antibody responses through specific T-cell helper functions. This is consistent with the experimental finding that a single amino acid change in HA1 of H3N2 can abrogate both antibody binding and CD4+ response and that there was frequent coincidence of B and T-cell epitopes on HA [23], [48]. Overlap of CD8+ epitopes with B-cell epitopes has also been documented in other influenza A viruses [49]. In the case of vaccinia, Sette *et al* have demonstrated a deterministic linkage between a CD4+ epitope providing specific help to B-cells binding an epitope located in the same protein [50]. Our own *in silico* analyses of many organisms show the close association of B-cell epitopes and predicted high affinity MHC binding sites in coincident epitope groups [34].

**Figure 13:**
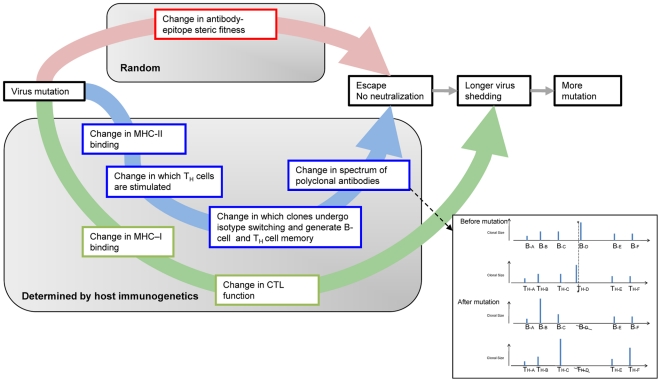
Summary of pathways for antigenic escape from immunologic pressure. The flow diagram summarizes the consequences of virus mutation. The inset panel is a hypothetical example of the change in the polyclonal spectrum of antibody and T-helper cells as a result of mutation. A mutation in the viral protein at the position marked by the asterisk and dotted line causes loss of MHC binding at that position and hence no further stimulation of T helper clone TH-D and hence loss of antibody of the clonotype B–D.

The possible pathways of immune pressure and escape of a mutating virus are summarized in Figure 13. A mutated virus has an altered binding interface with an existing antibody (see red pathway in Figure 13) but through changed T-cell helper functions (blue pathway), it may also recall a different spectrum of B-memory, and simultaneously stimulate a different *de novo* spectrum of B-cells from those of its un-mutated predecessor. While escape from pre-existing antibody is essentially the direct random effect of mutation, the response mediated by CD4+T helper cells is defined by host HLA and, as we show, may vary between hosts of different HLA. Mutation also has the capability to change CD8+ mediated immune pressure (green pathway), although this may be more important for other influenza proteins.

We should be cautious in extrapolating based on analysis of one protein. However, the hemagglutinin protein presents a special case as the primary site of binding of neutralizing antibody and cell entry receptors. The essential role of B-cells together with CD4+ cells in enabling a T-dependent antibody response and recovery from influenza infection has been shown [16], [51]–[53]. Influenza does not infect normal B-cells [32], which implies that their role as APC differs from that of dendritic cells and macrophages which are exposed to the full gamut of viral proteins during infection or phagocytosis [29]. Recognition by B-cell receptors of hemagglutinin, followed by internalization, leads to exposure to and/or uptake of coincident MHC binding peptides allowing immediate MHC binding, presentation to T-cells, and recruitment of T-helper cells [54]. Following re-infection with a virus where one or more amino acid changes have occurred in a cognate T-cell epitope, the change in MHC-peptide binding affinity may skew the balance of T-helper clones (see Figure 13 inset). This may favor selected clonotypes among the original polyclone of B-memory cells, and lead to either escape or heterosubtypic immunity. This suggests that host immunogenetics play a more active and possibly determinant role in antigenic drift of influenza viruses. In a recent analogous study, Tong et al have described the role of MHC-I driven evolution of Chikungunya virus [55].

The focus on MHC binding brings to the fore consideration of the diversity of HLA. Figures 5 and 6 show that each HLA has a different response to the peptides in any specific virus isolate, indicating that the outcome of infection is unique to the virus-host combination. Figures 10,11,12 show how over 34 years the pattern of change in MHC binding across all alleles is generally in the same regions of the protein, but that the detail for each HLA allele is different. A single amino acid change may cause a gain of binding sites for some HLAs but loss for others. Most hosts are heterozygous at each locus of the HLA and each allele will have a compensating effect. Likewise within a mixed population different pressures arise from each successive heterozygous host causing a different selection pressure. Indeed the outcome of infection may depend not only on the conjunction of the strain of virus with the HLA alleles of the current host, but on the selective pressure applied by the prior host's immunogenetics. We can also envision that a less genetically diverse population may elicit a more directional selection. There are examples of this for other viruses [56].

While several models of the evolution of H3N2 have been proposed [9], [11], [57]–[60], they rely on analysis of virus genotype and antigenicity as determined only by antibody binding. These previous approaches allow modeling of many features of strain emergence. However, they do not take full account of how changes in host genotype also exert selective pressure. This has been examined experimentally for single HLA peptide interactions [27]. A number of studies have identified key amino acid changes in HA1 associated with antibody escape [9], [11], [14]; however each amino acid change potentially impacts the binding of MHC-II molecules in 15 registers, each with the potential to affect T-cell helper function (Figure 3). Hence our findings reflect a more complex picture in which potential T-cell epitopes come and go with amino acid changes, and do so in a pattern that is unique to each HLA allele. This pattern is not readily appreciated by experimental single epitope characterization.

The hierarchies of MHC binding patterns (Figure 6) show that some HLA have a consistently lower absolute binding affinity to HA1 than others and some are more variable than others. Whether this translates to changes in CD4+ response or clinical outcome is not known. A number of field observations have linked severity of influenza or vaccine response to HLA [61]–[64] but given the number of variables and the small size of some of these studies, no definitive conclusions could be drawn. Patients with DRB1*09:01 have been reported to respond less well to vaccination with H3N2 [65], as have HLA-DR3 and DR4 patients [66]. We show DRB3*01:01 and DRB1*13:02 to be consistently lower affinity binders than other HLAs.

We are not aware of any specific studies in which HLA is linked to duration of influenza viral shedding although the diversity of HLA-determined T-cell response suggests variation is likely. Immunocompromised hosts have been observed to shed influenza A virus longer [67]–[69]. As a further step, experimental studies in horses [70] and birds [71] have shown that longer shedding is associated with the emergence of more new variant viruses. A small extension of shedding on an individual basis could have a significant compound effect in a population on the eventual number of new variants generated over time. This points to the possibility of immunogenetically based “incubator” individuals or populations which have a greater propensity to seed new virus variants for spread into global circulation. Other infections or diseases which affect B-cell numbers, function or susceptibility to infection could also be expected to change the selective process invoked by T-helper responses [32], [72].

Understanding the causes and extent of immunogenetically driven variation in the response to influenza is important to the development of broadly effective vaccines [65]. Vaccinal epitopes should be selected to protect all immunogenetic groups and to provide heterosubtypic immunity. Also, if influenza vaccines focused primarily on eliciting a T-cell response are to be developed, whether from HA or other proteins, a prerequisite will be to have a clearer understanding of how much variation in T-cell response occurs among individuals. The methods described here offer a way to examine such variability. Efforts to generate a universal vaccine for influenza should take into consideration the possibility that a T-cell epitope closely associated with a neutralizing B-cell epitope may be needed to ensure memory.

This study presents predicted MHC binding data for only one of the proteins of influenza A virus; it clearly provokes broader large scale analysis of the complete array of influenza proteins and other important serogroups of the virus, as well as application as a cost effective screening method which can help focus additional experimental studies.

### Acronyms Used

ic_50_: inhibitory concentration 50.

AROC: Area under the receiver operator characteristic curve

σ: one standard deviation.

## Supporting Information

Figure S1 **Relationship between MHC binding predictions and curated epitope categorizations including redundant epitopes.**
(PDF)

Figure S2 **Relationship between B-cell epitope sequence predictions and curated epitope categorizations.**
(PDF)

Figure S3 Cluster dendrogram of predicted binding patterns of peptides in 447 HA1 to A*02:01.
(PDF)

Figure S4 Cluster dendrogram of predicted binding patterns of peptides in 447 HA1 to DRB1*04:01.
(PDF)

Figure S5 **Hierarchical cluster of predicted MHC-I and MHC-II binding to HA1 from 10 cluster representative isolates.**
(PDF)

Figure S6 **Predicted high affinity MHC-II binding peptides gained, lost and conserved in HA1 in transitions between temporal clusters of H3N2.**
(PDF)

Figure S7 **Position of peptides in which change in MHC-II binding occurs in cluster transition.**
(PDF)

Table S1 **Epitope set from IEDB used for validation.**
(PDF)

Table S2 **Example of data set showing change in predicted MHC binding affinity with cluster transition changes in amino acids.**
(PDF)

## References

[1] Suzuki Y, Ito T, Suzuki T, Holland RE, Chambers TM (2000). Sialic acid species as a determinant of the host range of influenza A viruses.. J Virol.

[2] Horimoto T, Kawaoka Y (1994). Reverse genetics provides direct evidence for a correlation of hemagglutinin cleavability and virulence of an avian influenza A virus.. J Virol.

[3] Tewari MK, Sinnathamby G, Rajagopal D, Eisenlohr LC (2005). A cytosolic pathway for MHC class II-restricted antigen processing that is proteasome and TAP dependent.. Nat Immunol.

[4] Tscherne DM, Garcia-Sastre A (2011). Virulence determinants of pandemic influenza viruses.. J Clin Invest.

[5] Gerhard W (2001). The role of the antibody response in influenza virus infection.. Curr Top Microbiol Immunol.

[6] Wiley DC, Wilson IA, Skehel JJ (1981). Structural identification of the antibody-binding sites of Hong Kong influenza haemagglutinin and their involvement in antigenic variation.. Nature.

[7] Ferguson NM, Anderson RM (2002). Predicting evolutionary change in the influenza A virus.. Nat Med.

[8] Smith DJ (2003). Applications of bioinformatics and computational biology to influenza surveillance and vaccine strain selection.. Vaccine.

[9] Smith DJ, Lapedes AS, de Jong JC, Bestebroer TM, Rimmelzwaan GF (2004). Mapping the antigenic and genetic evolution of influenza virus.. Science.

[10] Plotkin JB, Dushoff J, Levin SA (2002). Hemagglutinin sequence clusters and the antigenic evolution of influenza A virus.. Proc Natl Acad Sci U S A.

[11] Bush RM, Bender CA, Subbarao K, Cox NJ, Fitch WM (1999). Predicting the evolution of human influenza A.. Science.

[12] Lee MS, Chen JS (2004). Predicting antigenic variants of influenza A/H3N2 viruses.. Emerg Infect Dis.

[13] Chi XS, Bolar TV, Zhao P, Tam JS, Rappaport R (2005). Molecular evolution of human influenza A/H3N2 virus in Asia and Europe from 2001 to 2003.. J Clin Microbiol.

[14] Both GW, Sleigh MJ, Cox NJ, Kendal AP (1983). Antigenic drift in influenza virus H3 hemagglutinin from 1968 to 1980: multiple evolutionary pathways and sequential amino acid changes at key antigenic sites.. J Virol.

[15] Scherle PA, Palladino G, Gerhard W (1992). Mice can recover from pulmonary influenza virus infection in the absence of class I-restricted cytotoxic T cells.. J Immunol.

[16] Gerhard W, Mozdzanowska K, Furchner M, Washko G, Maiese K (1997). Role of the B-cell response in recovery of mice from primary influenza virus infection.. Immunol Rev.

[17] Thomas PG, Keating R, Hulse-Post DJ, Doherty PC (2006). Cell-mediated protection in influenza infection.. Emerg Infect Dis.

[18] McMichael AJ, Michie CA, Gotch FM, Smith GL, Moss B (1986). Recognition of influenza A virus nucleoprotein by human cytotoxic T lymphocytes.. J Gen Virol.

[19] Mozdzanowska K, Furchner M, Maiese K, Gerhard W (1997). CD4+ T cells are ineffective in clearing a pulmonary infection with influenza type A virus in the absence of B cells.. Virology.

[20] Belz GT, Wodarz D, Diaz G, Nowak MA, Doherty PC (2002). Compromised influenza virus-specific CD8(+)-T-cell memory in CD4(+)-T-cell-deficient mice.. J Virol.

[21] Babon JA, Cruz J, Orphin L, Pazoles P, Co MD (2009). Genome-wide screening of human T-cell epitopes in influenza A virus reveals a broad spectrum of CD4(+) T-cell responses to internal proteins, hemagglutinins, and neuraminidases.. Hum Immunol.

[22] Richards KA, Chaves FA, Krafcik FR, Topham DJ, Lazarski CA (2007). Direct ex vivo analyses of HLA-DR1 transgenic mice reveal an exceptionally broad pattern of immunodominance in the primary HLA-DR1-restricted CD4 T-cell response to influenza virus hemagglutinin.. J Virol.

[23] Barnett BC, Graham CM, Burt DS, Skehel JJ, Thomas DB (1989). The immune response of BALB/c mice to influenza hemagglutinin: commonality of the B cell and T cell repertoires and their relevance to antigenic drift.. Eur J Immunol.

[24] Gianfrani C, Oseroff C, Sidney J, Chesnut RW, Sette A (2000). Human memory CTL response specific for influenza A virus is broad and multispecific.. Hum Immunol.

[25] Voeten JT, Bestebroer TM, Nieuwkoop NJ, Fouchier RA, Osterhaus AD (2000). Antigenic drift in the influenza A virus (H3N2) nucleoprotein and escape from recognition by cytotoxic T lymphocytes.. J Virol.

[26] Boon AC, de MG, Graus YM, Fouchier RA, Sintnicolaas K (2002). Sequence variation in a newly identified HLA-B35-restricted epitope in the influenza A virus nucleoprotein associated with escape from cytotoxic T lymphocytes.. J Virol.

[27] Berkhoff EG, Geelhoed-Mieras MM, Jonges M, Smith DJ, Fouchier RA (2007). An amino acid substitution in the influenza A virus hemagglutinin associated with escape from recognition by human virus-specific CD4+ T-cells.. Virus Res.

[28] Berkhoff EG, Boon AC, Nieuwkoop NJ, Fouchier RA, Sintnicolaas K (2004). A mutation in the HLA-B*2705-restricted NP383-391 epitope affects the human influenza A virus-specific cytotoxic T-lymphocyte response in vitro.. J Virol.

[29] VanoOsten AR, McGill J, Legge KL (2010). Quantification of the frequency and multiplicity of infection of respiratory- and lymph node-resident dendritic cells during influenza virus infection.. PLoS One.

[30] Hamilton-Easton A, Eichelberger M (1995). Virus-specific antigen presentation by different subsets of cells from lung and mediastinal lymph node tissues of influenza virus-infected mice.. J Virol.

[31] Crowe SR, Turner SJ, Miller SC, Roberts AD, Rappolo RA (2003). Differential antigen presentation regulates the changing patterns of CD8+ T cell immunodominance in primary and secondary influenza virus infections.. J Exp Med.

[32] Nimmerjahn F, Dudziak D, Dirmeier U, Hobom G, Riedel A (2004). Active NF-kappaB signalling is a prerequisite for influenza virus infection.. J Gen Virol.

[33] Bremel RD, Homan EJ (2010). An integrated approach to epitope analysis I: Dimensional reduction, visualization and prediction of MHC binding using amino acid principal components and regression approaches.. Immunome Res.

[34] Bremel RD, Homan EJ (2010). An integrated approach to epitope analysis II: A system for proteomic-scale prediction of immunological characteristics.. Immunome Res.

[35] Lund O, Nielsen M, Lundegaard C, Kesmir C, Brunak S (2005). Immunological Bioinformatics.

[36] SAS Institute Inc (2010). JMP® 9 Modelling and Multivariate Methods.

[37] Wold S, Sjorstrom M, Eriksson L (2001). PLS-regression: a basic tool of chemometrics.. Chemometrics and Intelligent Laboratory Systems.

[38] Greenbaum J, Sidney J, Chung J, Brander C, Peters B (2011). Functional classification of class II human leukocyte antigen (HLA) molecules reveals seven different supertypes and a surprising degree of repertoire sharing across supertypes.. Immunogenetics.

[39] Pham DT, Dimov S, Nguyen CD (2005). Selection of K in K-means clustering.. J Mech Eng Sci.

[40] Savitzky A, Golay MJE (1964). Smoothing and differentiation of data by simplified least squares procedures.. Anal Chem.

[41] Desmet J, Meersseman G, Boutonnet N, Pletinckx J, De CK (2005). Anchor profiles of HLA-specific peptides: analysis by a novel affinity scoring method and experimental validation.. Proteins.

[42] Johnson NL (1949). Systems of frequency curves generated by methods of translation.. Biometrika.

[43] Baumgartner CK, Ferrante A, Nagaoka M, Gorski J, Malherbe LP (2010). Peptide-MHC class II complex stability governs CD4 T cell clonal selection.. J Immunol.

[44] Lazarski CA, Chaves FA, Jenks SA, Wu S, Richards KA (2005). The kinetic stability of MHC class II:peptide complexes is a key parameter that dictates immunodominance.. Immunity.

[45] Constant SL, Bottomly K (1997). Induction of Th1 and Th2 CD4+ T cell responses: the alternative approaches.. Annu Rev Immunol.

[46] Roti M, Yang J, Berger D, Huston L, James EA (2008). Healthy human subjects have CD4+ T cells directed against H5N1 influenza virus.. J Immunol.

[47] Crowe SR, Miller SC, Brown DM, Adams PS, Dutton RW (2006). Uneven distribution of MHC class II epitopes within the influenza virus.. Vaccine.

[48] Barnett BC, Hartlmayr I, Graham CM, Thomas DB (1990). Single amino acid residues in a synthetic peptide of influenza haemagglutinin, HA 1 177–199, distinguish I-Ad- and I-Ed-restricted T-cell epitopes.. Immunology.

[49] Hioe CE, Dybdahl-Sissoko N, Philpott M, Hinshaw VS (1990). Overlapping cytotoxic T-lymphocyte and B-cell antigenic sites on the influenza virus H5 hemagglutinin.. J Virol.

[50] Sette A, Moutaftsi M, Moyron-Quiroz J, McCausland MM, Davies DH (2008). Selective CD4+ T cell help for antibody responses to a large viral pathogen: deterministic linkage of specificities.. Immunity.

[51] Topham DJ, Doherty PC (1998). Clearance of an influenza A virus by CD4+ T cells is inefficient in the absence of B cells.. J Virol.

[52] Mozdzanowska K, Furchner M, Zharikova D, Feng J, Gerhard W (2005). Roles of CD4+ T-cell-independent and -dependent antibody responses in the control of influenza virus infection: evidence for noncognate CD4+ T-cell activities that enhance the therapeutic activity of antiviral antibodies.. J Virol.

[53] Mozdzanowska K, Furchner M, Maiese K, Gerhard W (1997). CD4+ T cells are ineffective in clearing a pulmonary infection with influenza type A virus in the absence of B cells.. Virology.

[54] Batista FD, Iber D, Neuberger MS (2001). B cells acquire antigen from target cells after synapse formation.. Nature.

[55] Tong JC, Simarmata D, Lin RT, Renia L, Ng LF (2010). HLA class I restriction as a possible driving force for Chikungunya evolution.. PLoS One.

[56] de Campos-Lima PO, Gavioli R, Zhang QJ, Wallace LE, Dolcetti R (1993). HLA-A11 epitope loss isolates of Epstein-Barr virus from a highly A11+ population.. Science.

[57] Bedford T, Cobey S, Beerli P, Pascual M (2010). Global migration dynamics underlie evolution and persistence of human influenza A (H3N2).. PLoS Pathog.

[58] Russell CA, Jones TC, Barr IG, Cox NJ, Garten RJ (2008). The global circulation of seasonal influenza A (H3N2) viruses.. Science.

[59] Koelle K, Cobey S, Grenfell B, Pascual M (2006). Epochal evolution shapes the phylodynamics of interpandemic influenza A (H3N2) in humans.. Science.

[60] Rambaut A, Pybus OG, Nelson MI, Viboud C, Taubenberger JK (2008). The genomic and epidemiological dynamics of human influenza A virus.. Nature.

[61] Lebiush M, Rannon L, Kark JD (1981). The relationship between epidemic influenza (A(H1N1) and ABO blood group.. J Hyg (Lond).

[62] Maruyama N, Yoshida Y, Inamatsu T, Fujita K, Handa S (2004). HLA-DR allotype is associated with responsiveness to influenza vaccine in elderly Japanese.. Geriat Gerentol Int.

[63] Gelder CM, Lambkin R, Hart KW, Fleming D, Williams OM (2002). Associations between human leukocyte antigens and nonresponsiveness to influenza vaccine.. J Infect Dis.

[64] Albright FS, Orlando P, Pavia AT, Jackson GG, Cannon Albright LA (2008). Evidence for a heritable predisposition to death due to influenza.. J Infect Dis.

[65] Poland GA, Ovsyannikova IG, Jacobson RM (2008). Immunogenetics of seasonal influenza vaccine response.. Vaccine.

[66] Ruben FL, Fireman P, LaPorte RE, Drash AL, Uhrin M (1988). Immune responses to killed influenza vaccine in patients with type 1 diabetes: altered responses associated with HLA-DR 3 and DR 4.. J Lab Clin Med.

[67] Giannella M, Alonso M, Garcia dV, Roa PL, Catalan P (2010). Prolonged viral shedding in pandemic influenza A(H1N1): clinical significance and viral load analysis in hospitalized patients.. Clin Microbiol Infect.

[68] Pinsky BA, Mix S, Rowe J, Ikemoto S, Baron EJ (2010). Long-term shedding of influenza A virus in stool of immunocompromised child.. Emerg Infect Dis.

[69] Li CC, Wang L, Eng HL, You HL, Chang LS (2010). Correlation of pandemic (H1N1) 2009 viral load with disease severity and prolonged viral shedding in children.. Emerg Infect Dis.

[70] Murcia PR, Baillie GJ, Daly J, Elton D, Jervis C (2010). Intra- and interhost evolutionary dynamics of equine influenza virus.. J Virol.

[71] Iqbal M, Xiao H, Baillie G, Warry A, Essen SC (2009). Within-host variation of avian influenza viruses.. Philos Trans R Soc Lond B Biol Sci.

[72] Cagigi A, Nilsson A, Pensieroso S, Chiodi F (2010). Dysfunctional B-cell responses during HIV-1 infection: implication for influenza vaccination and highly active antiretroviral therapy.. Lancet Infect Dis.

