# Supplementary material for: Patterns of Predicted T-Cell Epitopes Associated with Antigenic Drift in Influenza H3N2 Hemagglutinin
Source: PLoS One. 2011 Oct 24;6(10):e26711. doi: 10.1371/journal.pone.0026711 (PMC3200361; doi:10.1371/journal.pone.0026711)
Supplement: Figure S1 — Relationship between MHC binding predictions and curated epitope categorizations including redundant epitopes. (PDF) [file pone.0026711.s001.pdf]

Homan and Bremel, Supporting materials

Figure S1: Relationship between MHC binding predictions and curated epitope categorizations including redundant epitopes.

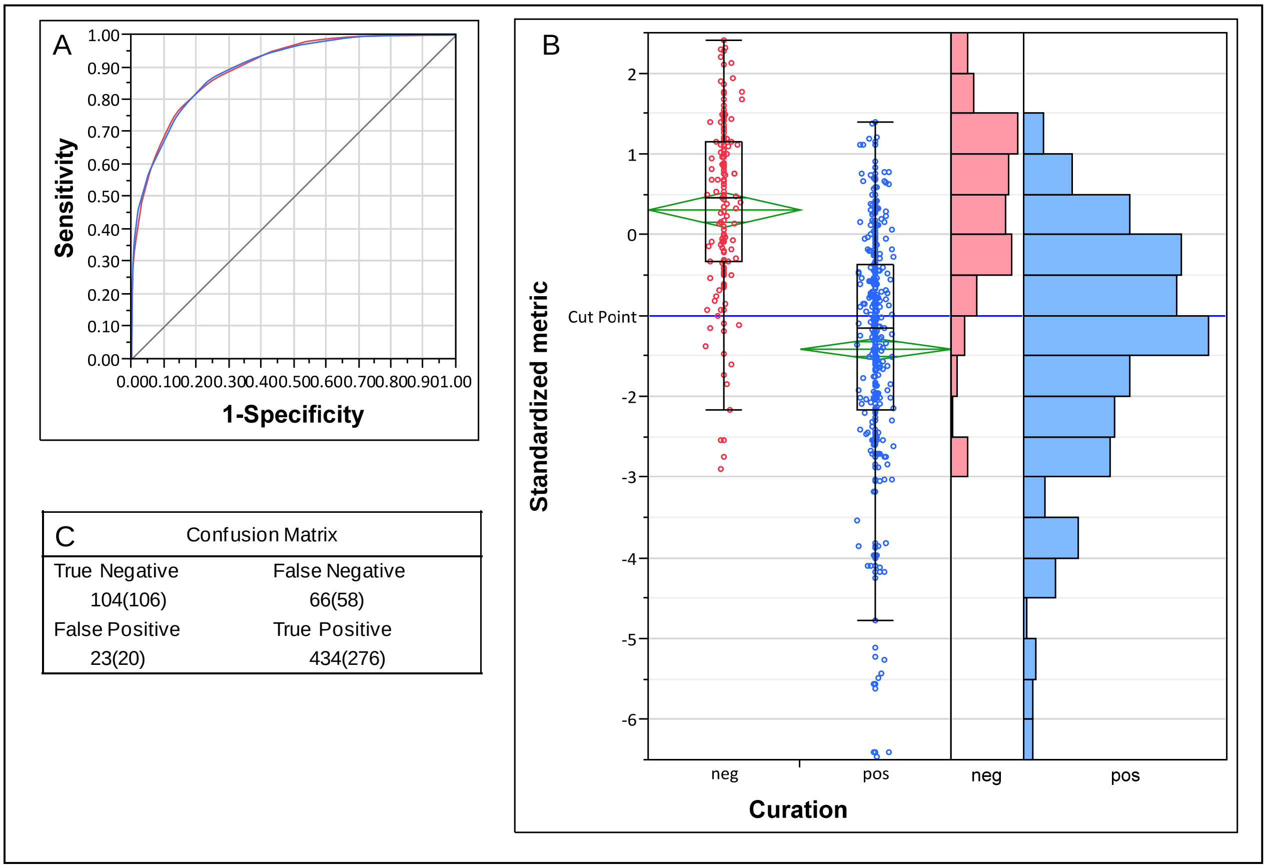

True Negative 104(106)	False Negative 66(58)
False Positive 23(20)	True Positive 434(276)

Panel A. The area under the ROC curve of 0.89. **Panel B**. The chart shows that the mean of the positive class was approximately -1.5σ (center of the diamond). The mean of the negative class is approximately +0.4σ. The ends of the boxes are the 25th and 75th percentile and the horizontal line in the box is the median. The ends of the lines are the 5th and 95th percentiles of the respective distributions. A cut-point of -0.53σ was chosen by the recursive categorization process. The cutpoint at -1σ (solid line) is a more rigorous threshold which was used as a readily understandable criterion throughout the study. **Panel C**. The confusion matrix documents agreement and disagreement between predictive and experimental methods. Numbers in parenthesis are the non-redundant data.

The NN-predicted binding affinity as the $\ln(ic_{50})$ was computed for a total of 627 peptides that had been curated and classified as either positive or negative according to their recorded experimental activity in one of several different bioassays as described in Table S1. The neural net is trained on and can only make predictions for 9-mers or 15-mers, peptides longer than these lengths were broken into a series of peptides of the appropriate length and binding affinities were computed for each sub-peptide for the particular HLA under consideration. The sub-peptide with the highest affinity is used in the further analysis. The implicit assumption is that a 20-mer will adopt a binding registration in the binding pocket

for which the MHC has the highest affinity and that a 15-mer embedded within a 20-mer will behave like 15-mer in isolation. The binding affinity data was standardized to zero mean and unit variance to account for the substantially different means and variance of the $\ln(ic_{50})$ data from different MHC alleles. The standardized binding predictions (in standard deviation units) and the curated classification were then analyzed using the recursive partitioning platform of JMP. A 5 k-fold cross-validation analysis was carried out and the partitioning algorithms were set to choose the optimum cut-point for classification.

As noted in the body of the paper this dataset has a high level of redundancy and contains peptides that have been tested multiple times by different laboratories and using different methods. We were concerned that this redundancy could bias the comparison with the predictions. In the confusion matrix (Panel C) elements where the numbers in parenthesis are the non-redundant data. A total of 158 true positive results were removed to create the non-redundant dataset described in the main body of the text, while very few true negatives, false negatives or false positives were removed. The overall effects on the prediction accuracy is relatively modest, with a slightly higher degree of agreement in the redundant dataset. The AROC of the redundant data set is 0.898 as compared to 0.848 for the non-redundant set.
