# Supplementary material for: Patterns of Predicted T-Cell Epitopes Associated with Antigenic Drift in Influenza H3N2 Hemagglutinin
Source: PLoS One. 2011 Oct 24;6(10):e26711. doi: 10.1371/journal.pone.0026711 (PMC3200361; doi:10.1371/journal.pone.0026711)
Supplement: Figure S2 — Relationship between B-cell epitope sequence predictions and curated epitope categorizations. (PDF) [file pone.0026711.s002.pdf]

Figure S2: Relationship between B-cell epitope sequence predictions and curated epitope categorizations.

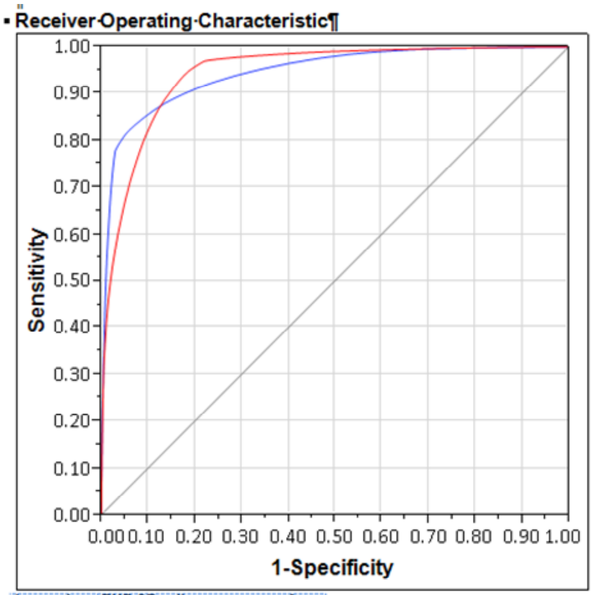

Legend	EXP SP adj	Area
(blue line)	.	0.9454
(red line)	1	0.9454

Partition for EXP SP adj 10 HA1 from H3N2 over 35 years

RSquare	N	Number of Splits	Imputes	AICc
0.603	3438	241	554	6013.31

Crossvalidation

k-fold		-2LogLike	RSquare
5	Folded	1279.89616	0.5204
	Overall	993.944202	0.6025

Fit Details

Measure	Training	Definition
Entropy RSquare	0.6025	1-Loglike(model)/Loglike(0)
Generalized R-Square	0.6919	(1-(L(0)/L(model))^(2/n))/(1-L(0)^(2/n))
Mean -Log p	0.1542	∑ -Log(ρ[j])/n
RMSE	0.2212	√ ∑(y[j]-ρ[j])²/n
Mean Abs Dev	0.1056	∑ |y[j]-ρ[j]|/n
Misclassification Rate	0.0710	∑ (ρ[j]≠ρMax)/n
N	3438	n

Confusion Matrix

True Negative	False Negative
2943	45
**False Positive**	**True Positive**
199	251

The 10 proteins used as cluster representatives (Table 1) were used to compare the BEPI predictions with experimental B-cell epitope (BEPI)contact points. BEPI contact locations were obtained from Smith et *al* [1] Table I and adjusted for the presence of a signal peptide in all protein amino acid coordinates in the current work. The B-cell epitopes of H3N2 HA1 have been well characterized for several isolates [2]. A code of 1 was given to each amino acid location in the prediction arrays that matched these positions and all other positions were treated as missing data and coded as 0 (no negative predictions were made). A NN based on training with BepiPred 1.0 output as described previously [3,4] was used to compute a probability value for each amino acid in the primary sequence to be found within a BEPI and these probabilities were then standardized to zero mean and unit variance. This also uses amino acid principal components and neural network (NN) predictions, trained and cross validated on the output of a large random peptide set submitted to BepiPred 1.0 (cbs.dtu.dk/services/BepiPred) [4]. This NN (a BepiPred 1.0 mimic) was then used to predict sites in the influenza HA1 proteins associated with antibody binding domains and which, when mutated, are associated with large changes in the binding patterns of polyclonal antibodies used as standard references [1]. As BepiPred relies heavily on the work of Parker [5] and Hopp and Woods [6], it is effectively a structural predictor. The output is not an “epitope” prediction *per se*, but rather a probability that a particular amino acid (within a window ± 4 amino acids) is on the outside of a protein and thus could be a contact point for a binding antibody. It does not predict whether such amino acids are participants in a more complex 3-D epitope configuration.

As described above for MHC binding predictions(Supplemental S1), the performance of the B-cell epitope predictor was evaluated as the AROC and a confusion matrix determined. The standardized probabilities were then used as predictors of BEPI contact points by 5 k-fold training in the recursive partitioning platform of JMP. The recursive partitioning platform was set to seek the optimum cut-point in the probability data for predicting a potential BEPI contact point. A total of 3438 predictions were made for the 10 proteins with 45 marked contact points per protein. The overall statistics are given in the Figure panel above. The optimum cut-point was found to be 1.3 standard deviation units. The AROC was calculated as 0.945 and the resulting true positive rate was 0.6. Experimental testing revealed that the K-fold cross validation process which is the best to use for these purposes is somewhat compromised by the very large number of non-contact (missing) points relative (300 of 345 total) to true (45 of 345 total) contact points because the 20% random sample selected in the K-fold process can have highly variable numbers of true positives to work with.

Reference List

[1] Smith DJ, Lapedes AS, de Jong JC, Bestebroer TM, Rimmelzwaan GF, Osterhaus AD, Fouchier RA. Mapping the antigenic and genetic evolution of influenza virus. Science 2004; 305(5682): 371-6.

[2] Wiley DC, Wilson IA, Skehel JJ. Structural identification of the antibody-binding sites of Hong Kong influenza haemagglutinin and their involvement in antigenic variation. Nature 1981; 289(5796): 373-8.

[3] Bremel RD, Homan EJ. An integrated approach to epitope analysis II: A system for proteomic-scale prediction of immunological characteristics. Immunome Res 2010; 6(1): 8.

[4] Bremel RD, Homan EJ. An integrated approach to epitope analysis I: Dimensional reduction, visualization and prediction of MHC binding using amino acid principal components and regression approaches. Immunome Res 2010; 6(1): 7.

[5] Parker JM, Guo D, Hodges RS. New hydrophilicity scale derived from high-performance liquid chromatography peptide retention data: correlation of predicted surface residues with antigenicity and X-ray-derived accessible sites. Biochemistry 1986; 25(19): 5425-32.

[6] Hopp TP, Woods KR. Prediction of protein antigenic determinants from amino acid sequences. Proc Natl Acad Sci U S A 1981; 78(6): 3824-8.
