# Supplementary material for: Patterns of Predicted T-Cell Epitopes Associated with Antigenic Drift in Influenza H3N2 Hemagglutinin
Source: PLoS One. 2011 Oct 24;6(10):e26711. doi: 10.1371/journal.pone.0026711 (PMC3200361; doi:10.1371/journal.pone.0026711)

Figure S3: Cluster dendrogram of predicted binding patterns of peptides in 447 HA1 to A*02:01

HA1 from 447 H3N2 viruses covering the period for 1968 – 2002 were clustered by their ln(ic50) values for each HLA. Hierachical clustering using the Ward method was carried out using 23 clusters [3]. The cluster count was based on preliminary determination of optimum cluster number from K-means clustering. This analysis yielded optimum clusters numbers ranging from 17-23 depending on the HLA. For the remainder of analysis 23 clusters were used. The binding data for each HLA was first submitted to principal component analysis and the mean of the first principal component was used as an ordering variable [3].

(large figure on next page, zoom for detail)

Report: Untitled 23

Hierarchical Clustering

Method = Ward

Dendrogram

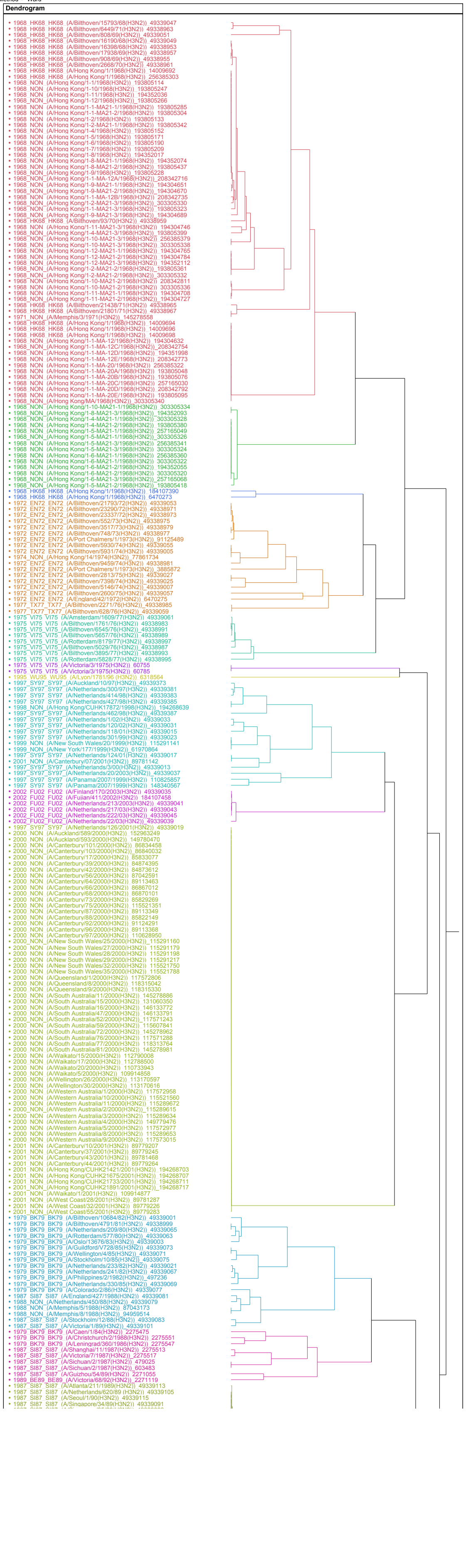

Report: Untitled 23

Hierarchical Clustering

Dendrogram

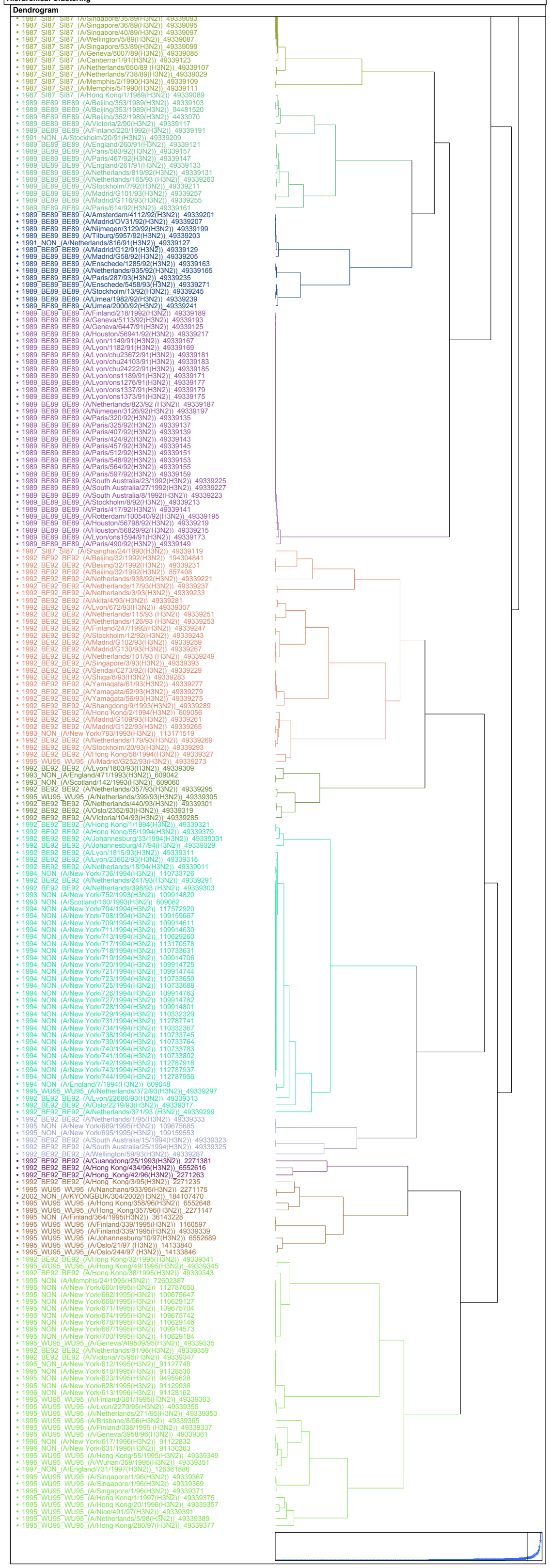

Supplement: Figure S3 — Cluster dendrogram of predicted binding patterns of peptides in 447 HA1 to A*02:01. (PDF) [file pone.0026711.s003.pdf]
