# Supplementary material for: Patterns of Predicted T-Cell Epitopes Associated with Antigenic Drift in Influenza H3N2 Hemagglutinin
Source: PLoS One. 2011 Oct 24;6(10):e26711. doi: 10.1371/journal.pone.0026711 (PMC3200361; doi:10.1371/journal.pone.0026711)
Supplement: Figure S5 — Hierarchical cluster of predicted MHC-I and MHC-II binding to HA1 from 10 cluster representative isolates. (PDF) [file pone.0026711.s005.pdf]

Figure S5: Hierarchical clusters of predicted MHC-I and MHC-II binding to HA1 from additonal cluster representative isolates.

Hierarchical clustering of predicted binding of multiple MHC-I and MHC-IIs to consecutive peptides in HA1 of each cluster representative virus listed in Table 1was done by the method of Ward using standardized binding data. The binding data for each HLA was first submitted to principal component analysis and the mean of the first principal component was used as an ordering variable. A comparable plots of binding to HA1 of Influenza A/Bilthoven/16190/68 is shown in Figure 3. The color scale shows the ln(ic50).

MHC-I

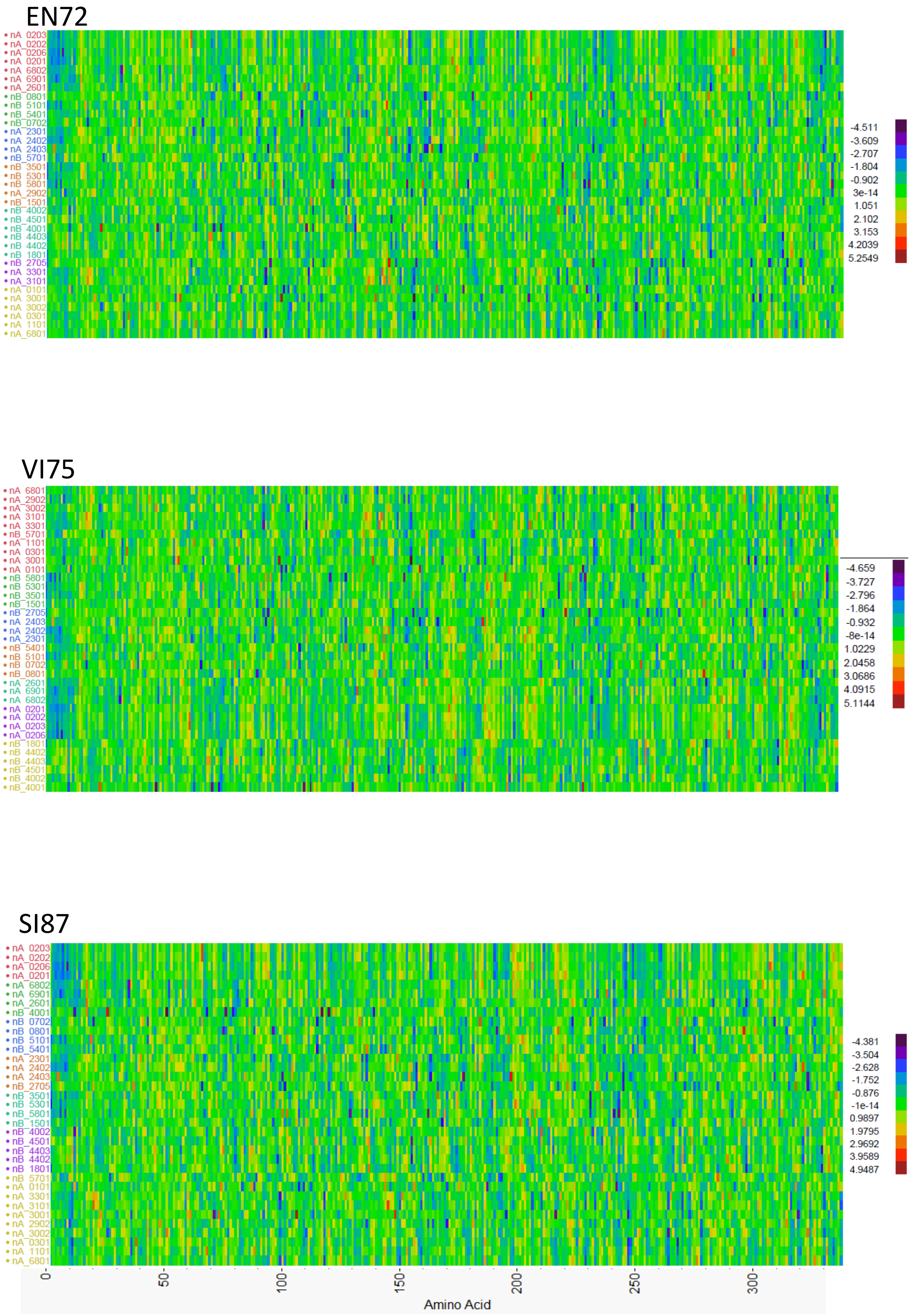
EN72
nA_0203
nA_0202
nA_0206
nA_0201
nA_6802
nA_6901
nA_2601
nB_0801
nB_5101
nB_5401
nB_0702
nA_2301
nA_2402
nA_2403
nB_5701
nB_3501
nB_5301
nB_5801
nA_2902
nB_1501
nB_4002
nB_4501
nB_4001
nB_4403
nB_4402
nB_1801
nB_2705
nA_3301
nA_3101
nA_0101
nA_3001
nA_3002
nA_0301
nA_1101
nA_6801
-4.511
-3.609
-2.707
-1.804
-0.902
3e-14
1.051
2.102
3.153
4.2039
5.2549
VI75
nA_6801
nA_2902
nA_3002
nA_3101
nA_3301
nB_5701
nA_1101
nA_0301
nA_3001
nA_0101
nB_5801
nB_5301
nB_3501
nB_1501
nB_2705
nA_2403
nA_2402
nA_2301
nB_5401
nB_5101
nB_0702
nB_0801
nA_2601
nA_6901
nA_6802
nA_0201
nA_0202
nA_0203
nA_0206
nB_1801
nB_4402
nB_4403
nB_4501
nB_4002
nB_4001
-4.659
-3.727
-2.796
-1.864
-0.932
-8e-14
1.0229
2.0458
3.0686
4.0915
5.1144
SI87
nA_0203
nA_0202
nA_0206
nA_0201
nA_6802
nA_6901
nA_2601
nB_4001
nB_0702
nB_0801
nB_5101
nB_5401
nA_2301
nA_2402
nA_2403
nB_2705
nB_3501
nB_5301
nB_5801
nB_1501
nB_4002
nB_4501
nB_4403
nB_4402
nB_1801
nB_5701
nA_0101
nA_3301
nA_3101
nA_3001
nA_2902
nA_3002
nA_0301
nA_1101
nA_6801
-4.381
-3.504
-2.628
-1.752
-0.876
-1e-14
0.9897
1.9795
2.9692
3.9589
4.9487
0
50
100
150
200
250
300
Amino Acid

MHC-I

BK79

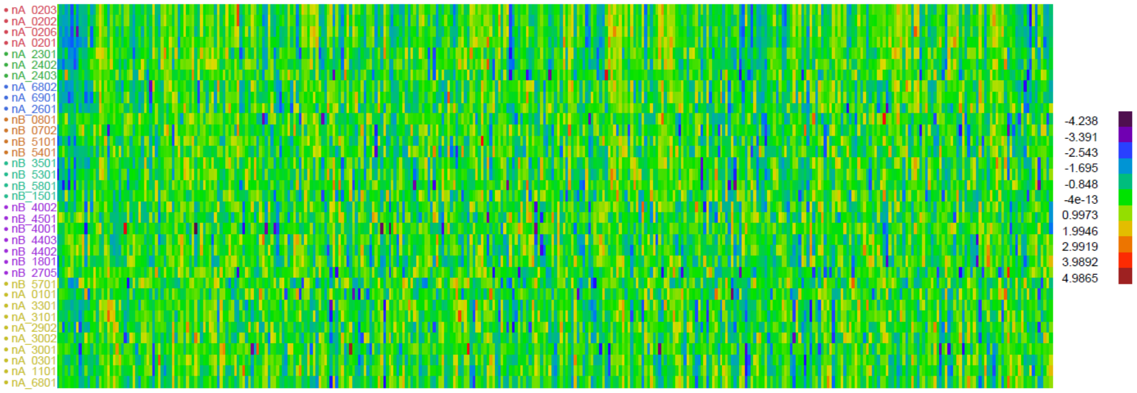

BE89

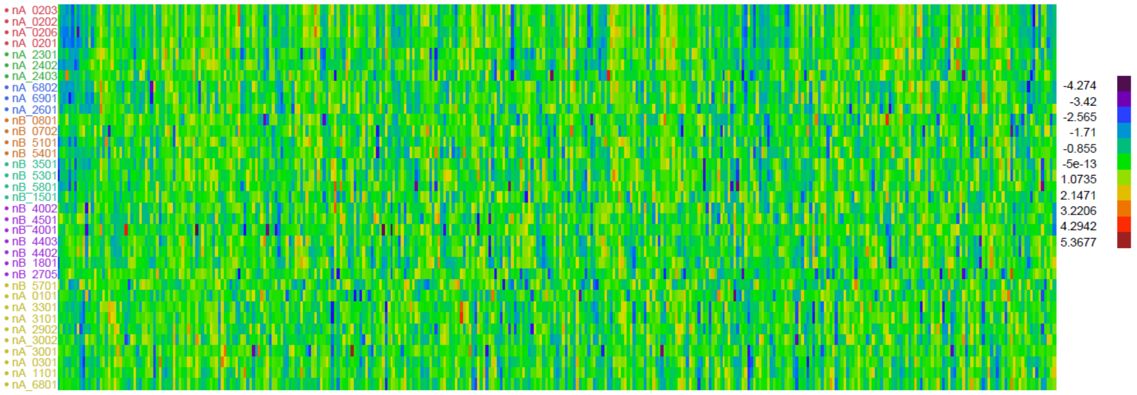

BE92

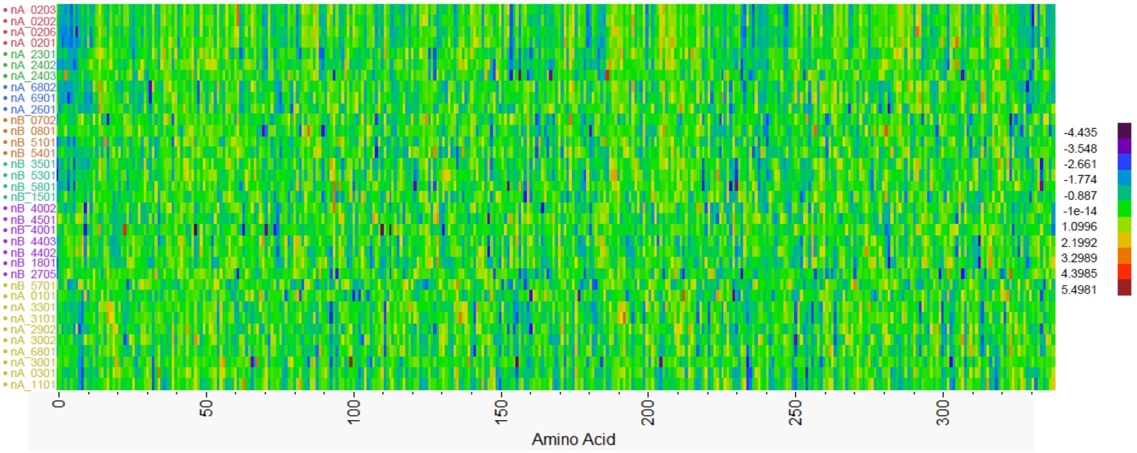

MHC-I

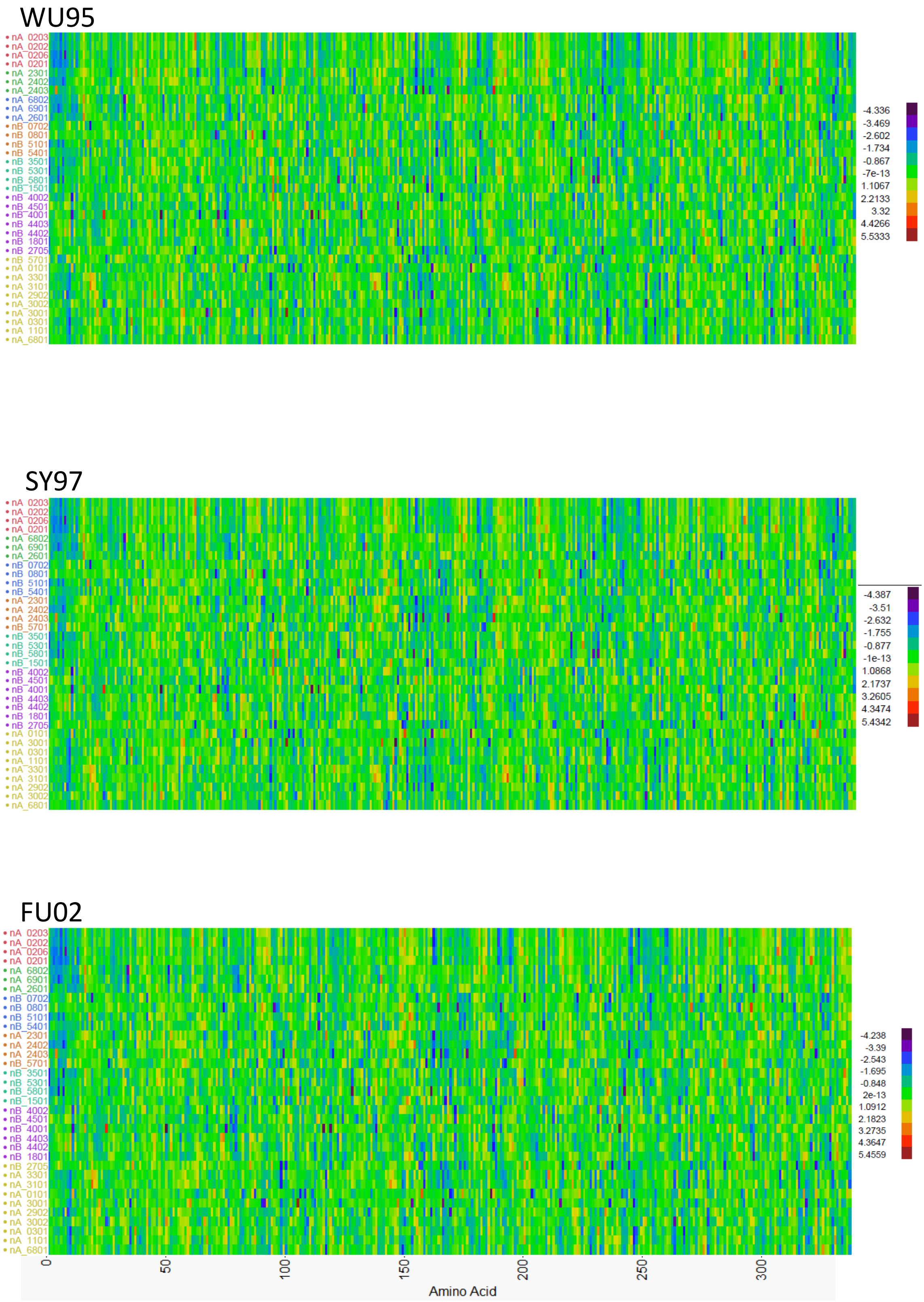

WU95
nA_0203
nA_0202
nA_0206
nA_0201
nA_2301
nA_2402
nA_2403
nA_6802
nA_6901
nA_2601
nB_0702
nB_0801
nB_5101
nB_5401
nB_3501
nB_5301
nB_5801
nB_1501
nB_4002
nB_4501
nB_4001
nB_4403
nB_4402
nB_1801
nB_2705
nB_5701
nA_0101
nA_3301
nA_3101
nA_2902
nA_3002
nA_3001
nA_0301
nA_1101
nA_6801
-4.336
-3.469
-2.602
-1.734
-0.867
-7e-13
1.1067
2.2133
3.32
4.4266
5.5333
SY97
nA_0203
nA_0202
nA_0206
nA_0201
nA_6802
nA_6901
nA_2601
nB_0702
nB_0801
nB_5101
nB_5401
nA_2301
nA_2402
nA_2403
nB_5701
nB_3501
nB_5301
nB_5801
nB_1501
nB_4002
nB_4501
nB_4001
nB_4403
nB_4402
nB_1801
nB_2705
nA_0101
nA_3001
nA_0301
nA_1101
nA_3301
nA_3101
nA_2902
nA_3002
nA_6801
-4.387
-3.51
-2.632
-1.755
-0.877
-1e-13
1.0868
2.1737
3.2605
4.3474
5.4342
FU02
nA_0203
nA_0202
nA_0206
nA_0201
nA_6802
nA_6901
nA_2601
nB_0702
nB_0801
nB_5101
nB_5401
nA_2301
nA_2402
nA_2403
nB_5701
nB_3501
nB_5301
nB_5801
nB_1501
nB_4002
nB_4501
nB_4001
nB_4403
nB_4402
nB_1801
nB_2705
nA_3301
nA_3101
nA_0101
nA_3001
nA_2902
nA_3002
nA_0301
nA_1101
nA_6801
-4.238
-3.39
-2.543
-1.695
-0.848
2e-13
1.0912
2.1823
3.2735
4.3647
5.4559
0
50
100
150
200
250
300
Amino Acid

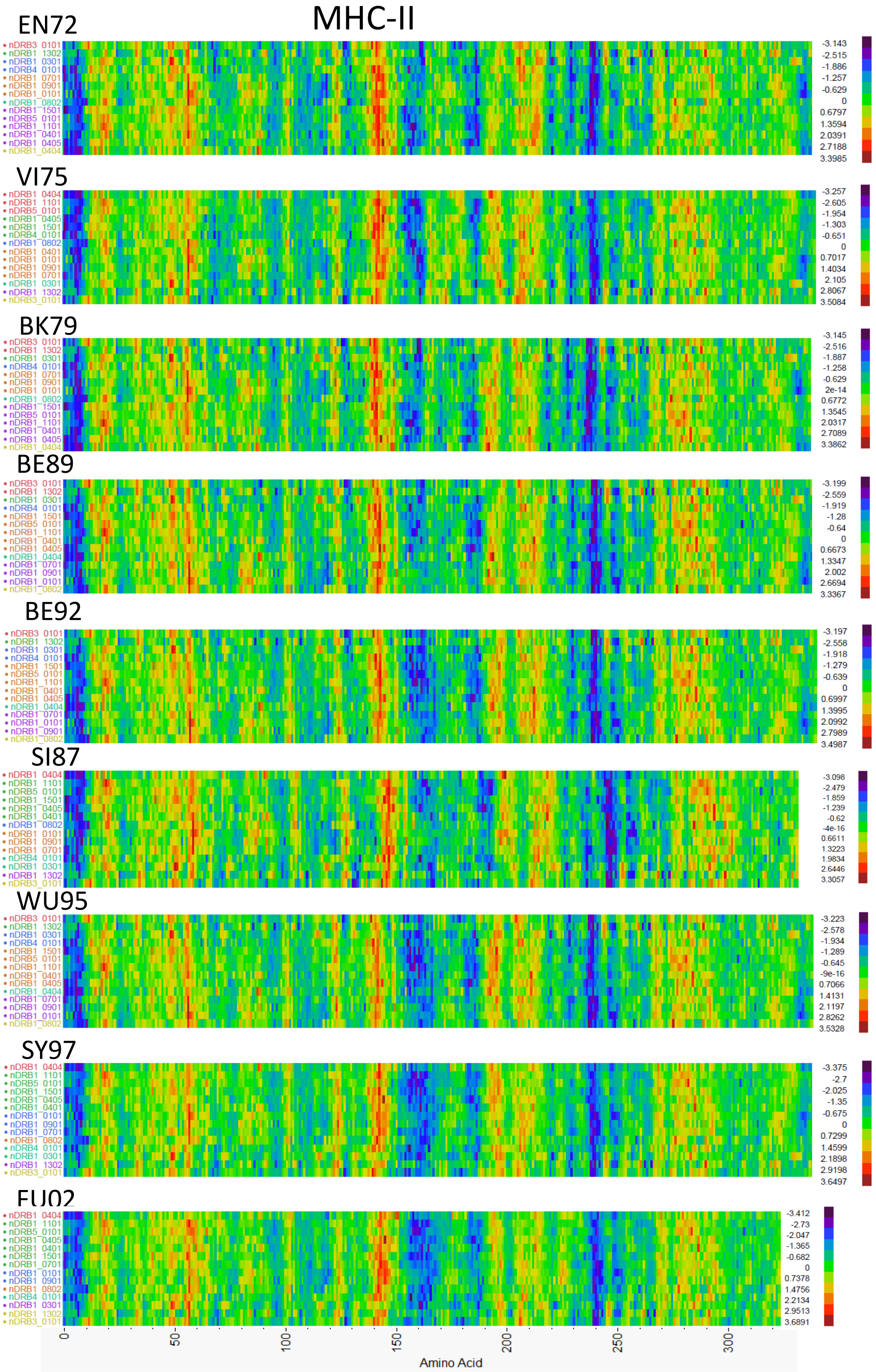
MHC-II
EN72
VI75
BK79
BE89
BE92
SI87
WU95
SY97
FU02
0
50
100
150
200
250
300
Amino Acid
