# Supplementary material for: Patterns of Predicted T-Cell Epitopes Associated with Antigenic Drift in Influenza H3N2 Hemagglutinin
Source: PLoS One. 2011 Oct 24;6(10):e26711. doi: 10.1371/journal.pone.0026711 (PMC3200361; doi:10.1371/journal.pone.0026711)
Supplement: Figure S6 — Predicted high affinity MHC-II binding peptides gained, lost and conserved in HA1 in transitions between temporal clusters of H3N2. (PDF) [file pone.0026711.s006.pdf]

Figure S6: Predicted high affinity MHC-II binding peptides gained, lost and conserved in HA1 in transitions between temporal clusters of H3N2.

For cluster representative viruses shown in Table 1 the scoring system shown in Table 2 was used to assign a categorical classification to predicted high affinity peptides according to whether they were new (-2), showed enhanced binding of an existing high binder (-1), conserved (0), showed reduced binding but were still high affinity (+1), or lost their status as predicted high affinity binders (+2). The plots show the aggregate number of each class of change in the HA1 protein associated with the transitions between clusters (HK68 to EN77, HK68 to FU02 etc) for 14 different MHC-II.

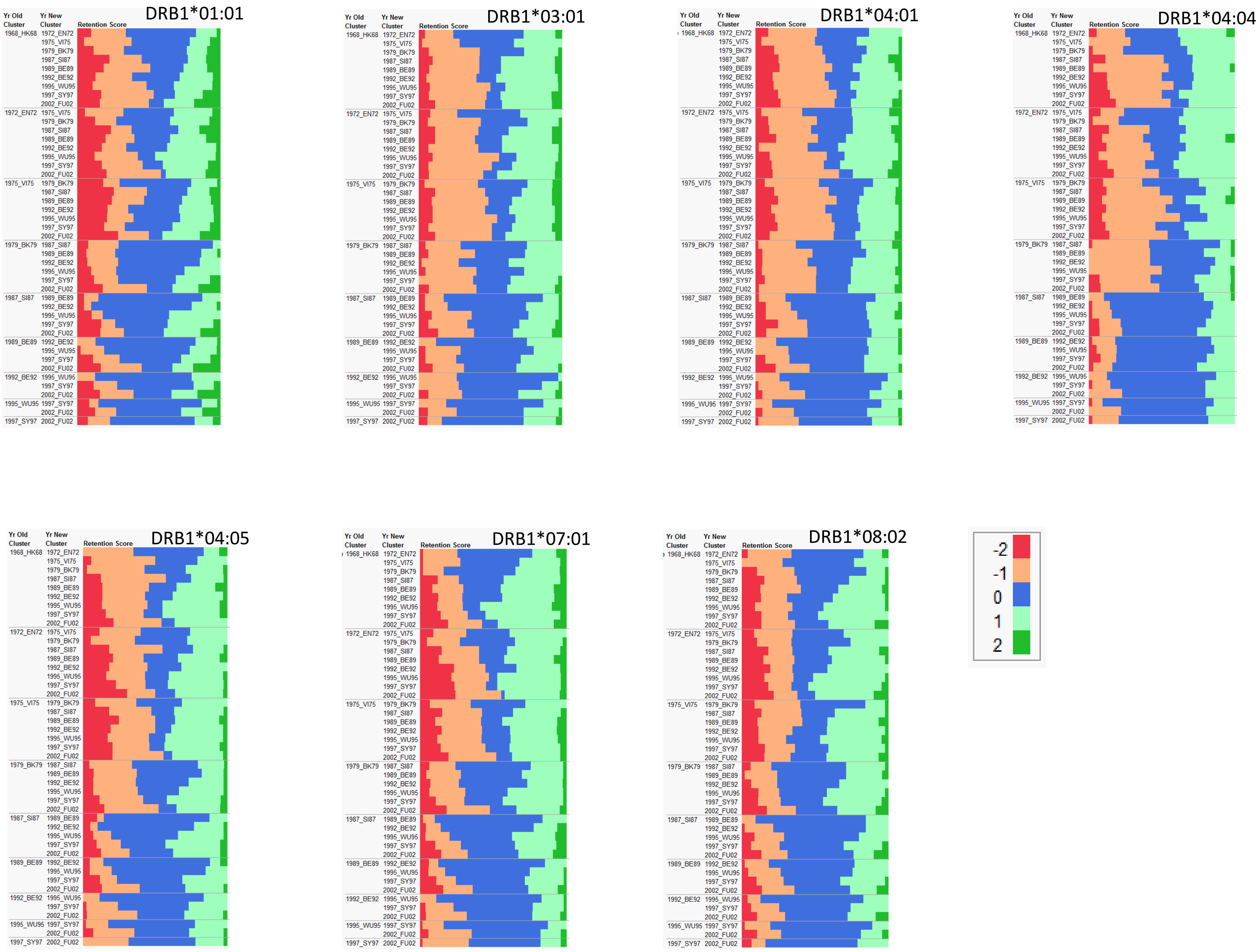
DRB1*01:01
DRB1*03:01
DRB1*04:01
DRB1*04:04
DRB1*04:05
DRB1*07:01
DRB1*08:02
Yr Old Cluster
Yr New Cluster
Retention Score
1968_HK68
1972_EN72
1975_VI75
1979_BK79
1987_SI87
1989_BE89
1992_BE92
1995_WU95
1997_SY97
2002_FU02
-2
-1
0
1
2

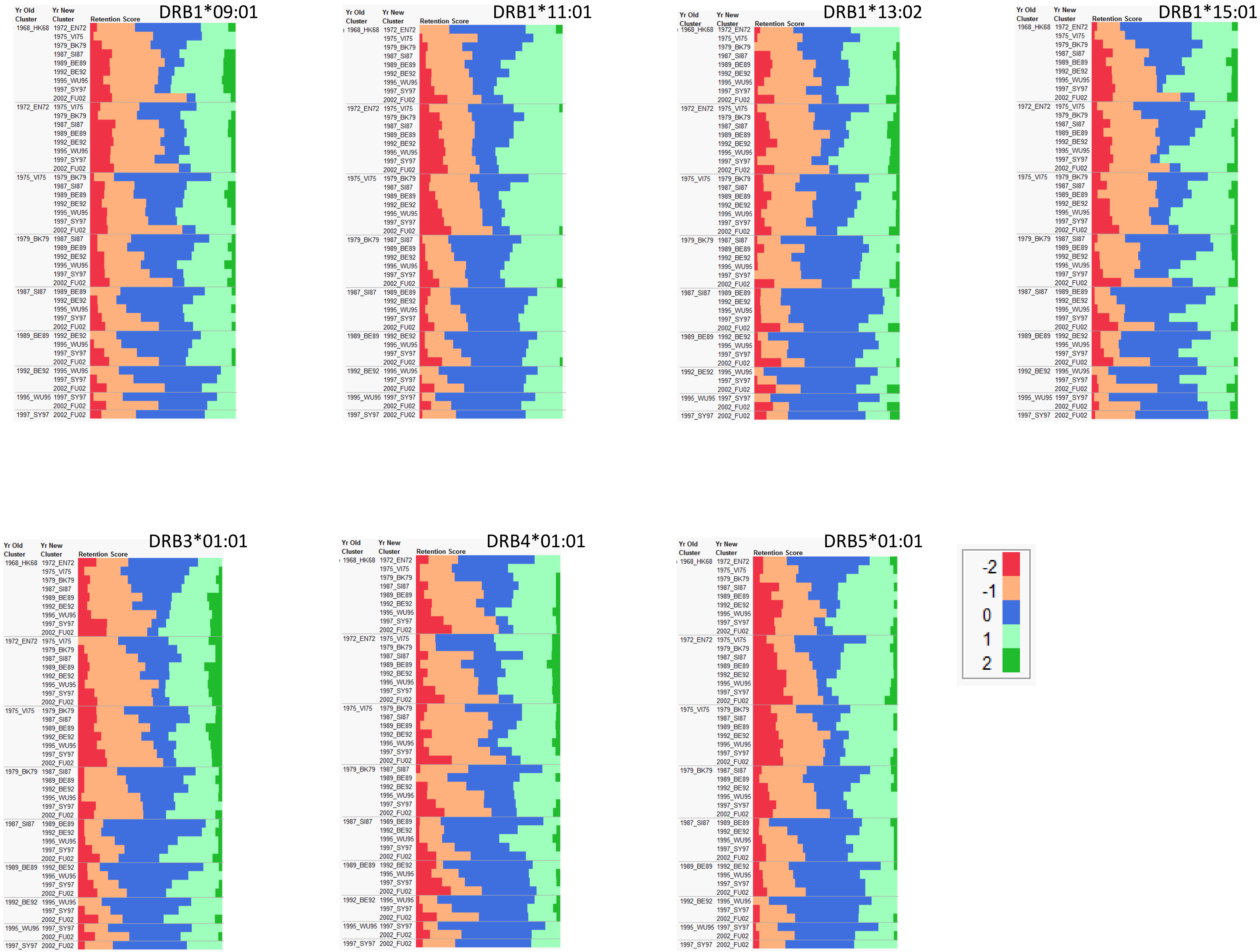
DRB1*09:01
DRB1*11:01
DRB1*13:02
DRB1*15:01
DRB3*01:01
DRB4*01:01
DRB5*01:01
Yr Old Cluster
Yr New Cluster
Retention Score
1968_HK68
1972_EN72
1975_VI75
1979_BK79
1987_SI87
1989_BE89
1992_BE92
1995_WU95
1997_SY97
2002_FU02
-2
-1
0
1
2
