# Supplementary material for: Patterns of Predicted T-Cell Epitopes Associated with Antigenic Drift in Influenza H3N2 Hemagglutinin
Source: PLoS One. 2011 Oct 24;6(10):e26711. doi: 10.1371/journal.pone.0026711 (PMC3200361; doi:10.1371/journal.pone.0026711)
Supplement: Figure S7 — Position of peptides in which change in MHC-II binding occurs in cluster transition. (PDF) [file pone.0026711.s007.pdf]

Figure S7: Position of peptides in which change in MHC-II binding occurs in cluster transition.

These figures correspond to Figure 8 A,B, C and show the moderate changes in high affinity binding (-1 and +1 categories). The categorical classification of peptides according to whether they gained or lost high affinity binder status (Table 2) as a result of the transition between two cluster representative viruses (Table 1) was plotted across all HA1 amino acid positions for 14 MHC-II's. Each panel of nine lanes shows the changes in binding for the cluster transition indicated at the top of the panel (HK68-EN77 etc). Each of the lanes within the panel is the change in predicted binding affinity for a different MHC-II allele, left to right DRB1*01:01, DRB1*03:01, DRB1*04:01, DRB1*04:04, DRB1*04:05, DRB1*07:01, DRB1*08:02, DRB1*09:01, DRB1*11:01, DRB1*13:02, DRB1*15:01, DRB3*01:01, DRB4*01:01, DRB5*01:01. The red lines on each side are amino acid positions identified by Smith *et al* as key mutation points for antigenic drift, these are taken from Smith's Table I and corrected for the presence of signal peptides. The color scale shown in the scale at left is the count of amino acid changes at a given position which result in the category of change mapped.

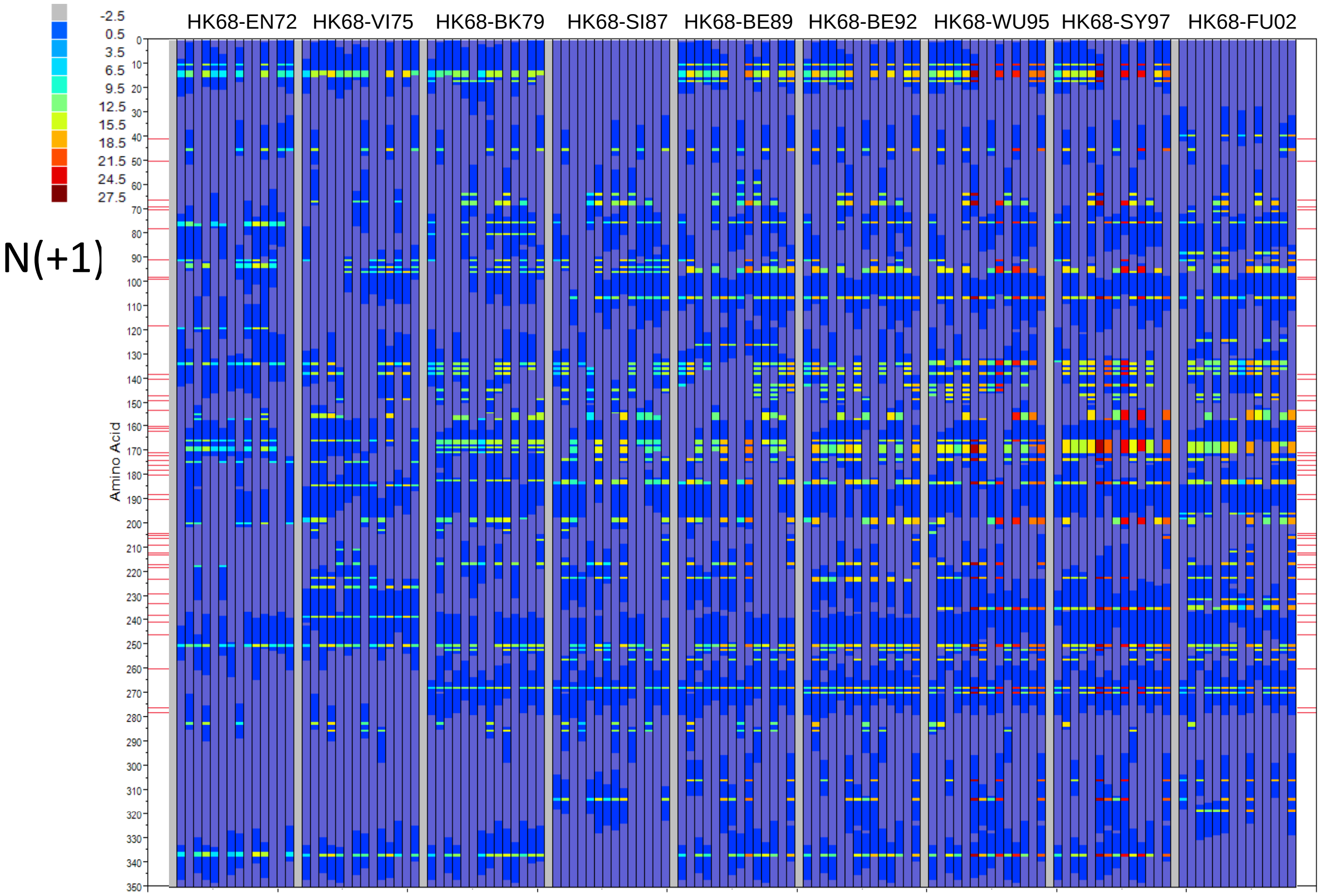
N(+1)
-2.5
0.5
3.5
6.5
9.5
12.5
15.5
18.5
21.5
24.5
27.5
HK68-EN72
HK68-VI75
HK68-BK79
HK68-SI87
HK68-BE89
HK68-BE92
HK68-WU95
HK68-SY97
HK68-FU02
Amino Acid

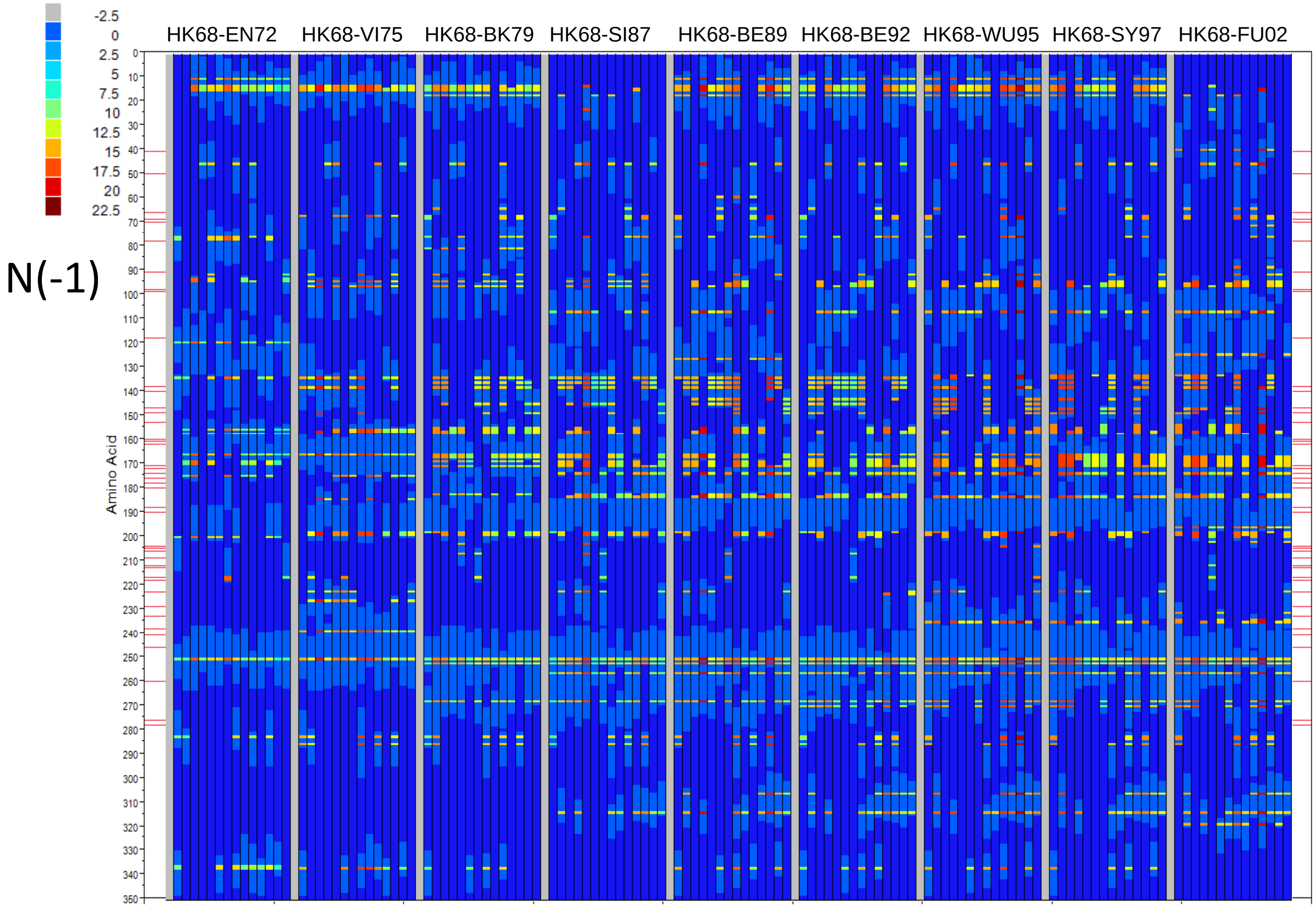
N(-1)
HK68-EN72
HK68-VI75
HK68-BK79
HK68-SI87
HK68-BE89
HK68-BE92
HK68-WU95
HK68-SY97
HK68-FU02
Amino Acid
-2.5
0
2.5
5
7.5
10
12.5
15
17.5
20
22.5
