# Supplementary material for: Patterns of Predicted T-Cell Epitopes Associated with Antigenic Drift in Influenza H3N2 Hemagglutinin
Source: PLoS One. 2011 Oct 24;6(10):e26711. doi: 10.1371/journal.pone.0026711 (PMC3200361; doi:10.1371/journal.pone.0026711)
Supplement: Table S1 — Epitope set from IEDB used for validation. (PDF) [file pone.0026711.s008.pdf]

Table S1: Epitope set from IEDB used for validation.

To generate a set of experimentally determined epitopes for comparison, we first downloaded complete IEDB records of all influenza A epitopes listed under "T-cell response". A subset was made which comprised records which had identified HLA (human or mice transgenic for HLAs) and limited to those comprising influenza isolates of hemagglutinin serotypes HA1, HA3 or HA5. Records were eliminated if the virus name was incomplete, unless the HA serotype was identified. We opted to restrict the list to publications or submissions dated 2000 or later. This was to provide a manageable number and to reduce nomenclature confusion.

These steps provided a list of 1228 records described in 35 publications and 5 groups of direct submissions. The list included many duplicate reports of the same epitope. Epitopes associated with seven publications were eliminated because the papers were designed to develop a new assay using control epitopes described by others, or where previously described epitopes were used in some secondary manner, for example to examine cross reactivity with non influenza epitopes.

As the designation of "positive" or "negative" made by IEDB denotes the response to a specific assay, we then manually curated the list by reference to the source publications. Some records listed as "positive" were removed because they identified a peptide status as an immunogen but not as an influenza epitope. Weak positives, some of which appeared in both negative and positive IEDB curations, were removed. Some negatives were removed as review of the experimental design showed negative did not equate to "non epitope" but was a function of experimental design (e.g. use as control in an adverse combination). Four additional positive records and seven additional negative records were identified from the publications. Assays used included Cr release, EPISPOT and tetramers.

The resultant list, shown below, comprised 457 positives and 171 negatives from 21 publications (PMIDs shown in table) and 3 groups of direct submissions to IEDB. The list contains duplicate entries for some epitopes, mostly positives, as IEDB records an entry for each experimental procedure. Comparative analysis was conducted twice, first using all records (including duplicates), and more conservatively, using only the unique epitope sequences. The unique sequences comprised 296 positives and 164 negatives. The outcome of the analysis based on the unique epitopes is shown in Figure S1.

IEDB Ref ID	PubMed ID	Curation	HLA	Epitope Linear Sequence	Method/Technique	MHC Allele Name	Protein	Virus_ID
931	10773346	pos	MHC I	FMYSDFHFI	51 chromium release	HLA-A*0201	PA	Influenza A virus (A/Puerto Rico/8/1934(H1N1))
931	10773346	pos	MHC I	FMYSDFHFI	51 chromium release	HLA-A*0201	PA	Influenza A virus (A/Puerto Rico/8/1934(H1N1))
931	10773346	pos	MHC I	FMYSDFHFI	51 chromium release	HLA-A*0201	PA	Influenza A virus (A/Puerto Rico/8/1934(H1N1))
931	10773346	pos	MHC I	GILGFVFTL	51 chromium release	HLA-A*0201	M1	Influenza A virus (A/Puerto Rico/8/1934(H1N1))
931	10773346	pos	MHC I	GILGFVFTL	51 chromium release	HLA-A*0201	M1	Influenza A virus (A/Puerto Rico/8/1934(H1N1))
931	10773346	pos	MHC I	GILGFVFTL	51 chromium release	HLA-A*0201	M1	Influenza A virus (A/Puerto Rico/8/1934(H1N1))
931	10773346	pos	MHC I	GLISLILQI	51 chromium release	HLA-A*0201	NA	Influenza A virus (A/Puerto Rico/8/1934(H1N1))
931	10773346	pos	MHC I	ILGFVFTLTV	51 chromium release	HLA-A*0201	M1	Influenza A virus (A/Puerto Rico/8/1934(H1N1))

931	10773346	pos	MHC I	ILGFVFTLTV	51 chromium release	HLA-A*0201	M1	Influenza A virus (A/Puerto Rico/8/1934(H1N1))
931	10773346	pos	MHC I	ILGFVFTLTV	51 chromium release	HLA-A*0201	M1	Influenza A virus (A/Puerto Rico/8/1934(H1N1))
931	10773346	pos	MHC I	NMLSTVLGV	51 chromium release	HLA-A*0201	PB1	Influenza A virus (A/Puerto Rico/8/1934(H1N1))
931	10773346	pos	MHC I	NMLSTVLGV	51 chromium release	HLA-A*0201	PB1	Influenza A virus (A/Puerto Rico/8/1934(H1N1))
931	10773346	pos	MHC I	SLCPIRGWAI	51 chromium release	HLA-A*0201	NA	Influenza A virus (A/Puerto Rico/8/1934(H1N1))
931	10773346	pos	MHC I	SLCPIRGWAI	51 chromium release	HLA-A*0201	NA	Influenza A virus (A/Puerto Rico/8/1934(H1N1))
931	10773346	pos	MHC I	SLENFRAYV	51 chromium release	HLA-A*0201	PA	Influenza A virus (A/Puerto Rico/8/1934(H1N1))
931	10773346	pos	MHC I	SLENFRAYV	51 chromium release	HLA-A*0201	PA	Influenza A virus (A/Puerto Rico/8/1934(H1N1))
931	10773346	pos	MHC I	GIAPLQLGK	51 chromium release	HLA-A11	HA	Influenza A virus (A/Puerto Rico/8/1934(H1N1))
931	10773346	pos	MHC I	KSMREEYRK	51 chromium release	HLA-A11	M2	Influenza A virus (A/Puerto Rico/8/1934(H1N1))
931	10773346	pos	MHC I	KSMREEYRK	51 chromium release	HLA-A11	M2	Influenza A virus (A/Puerto Rico/8/1934(H1N1))
931	10773346	pos	MHC I	RMVLASTTAK	51 chromium release	HLA-A11	M1	Influenza A virus (A/Puerto Rico/8/1934(H1N1))
931	10773346	pos	MHC I	RMVLASTTAK	51 chromium release	HLA-A11	M1	Influenza A virus (A/Puerto Rico/8/1934(H1N1))
931	10773346	pos	MHC I	RTLDFHDSNVK	51 chromium release	HLA-A11	HA	Influenza A virus (A/Puerto Rico/8/1934(H1N1))
931	10773346	pos	MHC I	RTLDFHDSNVK	51 chromium release	HLA-A11	HA	Influenza A virus (A/Puerto Rico/8/1934(H1N1))
931	10773346	pos	MHC I	RTLDFHDSNVK	51 chromium release	HLA-A11	HA	Influenza A virus (A/Puerto Rico/8/1934(H1N1))
931	10773346	pos	MHC I	RVLSFIKGTK	51 chromium release	HLA-A11	NP	Influenza A virus (A/Puerto Rico/8/1934(H1N1))
931	10773346	pos	MHC I	RVLSFIKGTK	51 chromium release	HLA-A11	NP	Influenza A virus (A/Puerto Rico/8/1934(H1N1))
931	10773346	pos	MHC I	RVLSFIKGTK	51 chromium release	HLA-A11	NP	Influenza A virus (A/Puerto Rico/8/1934(H1N1))
931	10773346	pos	MHC I	SIIPSGPLK	51 chromium release	HLA-A11	M1	Influenza A virus (A/Puerto Rico/8/1934(H1N1))
931	10773346	pos	MHC I	SIIPSGPLK	51 chromium release	HLA-A11	M1	Influenza A virus (A/Puerto Rico/8/1934(H1N1))
931	10773346	pos	MHC I	TMVMELVRMIK	51 chromium release	HLA-A11	NP	Influenza A virus (A/Puerto Rico/8/1934(H1N1))
931	10773346	pos	MHC I	VTAACSHAGK	51 chromium release	HLA-A11	HA	Influenza A virus (A/Puerto Rico/8/1934(H1N1))
931	10773346	pos	MHC I	VTAACSHAGK	51 chromium release	HLA-A11	HA	Influenza A virus (A/Puerto Rico/8/1934(H1N1))
893	10888619	pos	MHC I	SRYWAIRTR	51 chromium release	HLA-B27	NP	Influenza A virus H3N2
893	10888619	pos	MHC I	SRYWAIRTR	51 chromium release	HLA-B27	NP	Influenza A virus H3N2
893	10888619	pos	MHC I	SRYWAIRTR	51 chromium release	HLA-B27	NP	Influenza A virus H3N2
315643	11689620	pos	MHC I	GILGFVFTLT	51 chromium release	HLA-A*0201	M1	Influenza A virus (A/Puerto Rico/8/1934(H1N1))
315643	11689620	pos	MHC I	GILGFVFTLT	51 chromium release	HLA-A*0201	M1	Influenza A virus (A/Puerto Rico/8/1934(H1N1))
315643	11689620	pos	MHC I	CVNGSCFTV	51 chromium release	HLA-A*0201	NA	Influenza A virus (A/Puerto Rico/8/1934(H1N1))
315643	11689620	pos	MHC I	CVNGSCFTV	51 chromium release	HLA-A*0201	NA	Influenza A virus (A/Puerto Rico/8/1934(H1N1))
315643	11689620	pos	MHC I	CVNGSCFTV	51 chromium release	HLA-A*0201	NA	Influenza A virus (A/Puerto Rico/8/1934(H1N1))
315643	11689620	pos	MHC I	CVNGSCFTV	51 chromium release	HLA-A*0201	NA	Influenza A virus (A/Puerto Rico/8/1934(H1N1))
841	11752149	pos	MHC I	AIMDKNIIL	51 chromium release	HLA-A*0201	NS1	Influenza A virus H3N2 (A/Resvir-9 (H3N2))
841	11752149	pos	MHC I	CTELKLSDY	intracellular cytokine staining (ICS)	HLA-A1	NP	Influenza A virus H3N2 (A/Resvir-9 (H3N2))
841	11752149	pos	MHC I	CTELKLSDY	51 chromium release	HLA-A1	NP	Influenza A virus H3N2 (A/Resvir-9 (H3N2))
841	11752149	pos	MHC I	VSDGGPNLY	51 chromium release	HLA-A1	PB1	Influenza A virus H3N2 (A/Resvir-9 (H3N2))
841	11752149	pos	MHC I	ILRGSVAHK	51 chromium release	HLA-A3	NP	Influenza A virus H3N2 (A/Resvir-9 (H3N2))
841	11752149	pos	MHC I	SRYWAIRTR	51 chromium release	HLA-B*2705	NP	Influenza A virus H3N2 (A/Resvir-9 (H3N2))

841	11752149	pos	MHC I	GILGFVFTL	51 chromium release	HLA-B*3501	M1	Influenza A virus H3N2 (A/Resvir-9 (H3N2))
841	11752149	pos	MHC I	RRSGAAGAAVK	51 chromium release	HLA-B27	NP	Influenza A virus H3N2 (A/Resvir-9 (H3N2))
841	11752149	pos	MHC I	ELRSRYWAI	51 chromium release	HLA-B8	NP	Influenza A virus H3N2 (A/Resvir-9 (H3N2))
782	11836437	pos	MHC I	LPFDKSTIM	ELISPOT	HLA-B*3501	NP	Influenza A virus (A/Victoria/3/1975(H3N2))
782	11836437	pos	MHC I	LPFDKSTIM	51 chromium release	HLA-B*3501	NP	Influenza A virus (A/Victoria/3/1975(H3N2))
782	11836437	pos	MHC I	LPFDKPTIM	ELISPOT	HLA-B*3501	NP	Influenza A virus H3N2
782	11836437	pos	MHC I	LPFDKPTIM	51 chromium release	HLA-B*3501	NP	Influenza A virus H3N2
782	11836437	pos	MHC I	LPFEKSTVM	ELISPOT	HLA-B*3501	NP	Influenza A virus H3N2 (A/Resvir-9 (H3N2))
782	11836437	pos	MHC I	LPFEKSTVM	51 chromium release	HLA-B*3501	NP	Influenza A virus H3N2 (A/Resvir-9 (H3N2))
674	14764717	pos	MHC I	LPFDKPTIM	51 chromium release	HLA-B*3501	NP	Influenza A virus (A/nt/60/1968(H3N2))
674	14764717	pos	MHC I	LPFDKPTIM	MHC tetramer/multimer staining	HLA-B*3501	NP	Influenza A virus (A/nt/60/1968(H3N2))
674	14764717	pos	MHC I	LPFDKPTIM	MHC tetramer/multimer staining	HLA-B*3501	NP	Influenza A virus (A/nt/60/1968(H3N2))
674	14764717	pos	MHC I	LPFDKPTIM	MHC tetramer/multimer staining	HLA-B*3501	NP	Influenza A virus (A/nt/60/1968(H3N2))
674	14764717	pos	MHC I	LPFDKPTIM	MHC tetramer/multimer staining	HLA-B*3501	NP	Influenza A virus (A/nt/60/1968(H3N2))
674	14764717	pos	MHC I	LPFDKPTIM	MHC tetramer/multimer staining	HLA-B*3501	NP	Influenza A virus (A/nt/60/1968(H3N2))
674	14764717	pos	MHC I	LPFDKPTIM	MHC tetramer/multimer staining	HLA-B*3501	NP	Influenza A virus (A/nt/60/1968(H3N2))
674	14764717	pos	MHC I	LPFDKSTIM	51 chromium release	HLA-B*3501	NP	Influenza A virus (A/Texas/1/1977(H3N2))
674	14764717	pos	MHC I	LPFDKSTIM	MHC tetramer/multimer staining	HLA-B*3501	NP	Influenza A virus (A/Texas/1/1977(H3N2))
674	14764717	pos	MHC I	LPFDKSTIM	MHC tetramer/multimer staining	HLA-B*3501	NP	Influenza A virus (A/Texas/1/1977(H3N2))
674	14764717	pos	MHC I	LPFDKSTIM	MHC tetramer/multimer staining	HLA-B*3501	NP	Influenza A virus (A/Texas/1/1977(H3N2))
674	14764717	pos	MHC I	LPFEKSTVM	51 chromium release	HLA-B*3501	NP	Influenza A virus (A/Shanghai/16/1989(H3N2))
674	14764717	pos	MHC I	LPFEKSTVM	MHC tetramer/multimer staining	HLA-B*3501	NP	Influenza A virus (A/Shanghai/16/1989(H3N2))
674	14764717	neg	MHC I	LPFEKSTVM	MHC tetramer/multimer staining	HLA-B*3501	NP	Influenza A virus (A/Shanghai/16/1989(H3N2))
674	14764717	neg	MHC I	LPFEKSTVM	MHC tetramer/multimer staining	HLA-B*3501	NP	Influenza A virus (A/Shanghai/16/1989(H3N2))
674	14764717	neg	MHC I	LPFEKSTVM	MHC tetramer/multimer staining	HLA-B*3501	NP	Influenza A virus (A/Shanghai/16/1989(H3N2))
674	14764717	neg	MHC I	LPFEKSTVM	MHC tetramer/multimer staining	HLA-B*3501	NP	Influenza A virus (A/Shanghai/16/1989(H3N2))
674	14764717	neg	MHC I	LPFEKSTVM	MHC tetramer/multimer staining	HLA-B*3501	NP	Influenza A virus (A/Shanghai/16/1989(H3N2))
674	14764717	neg	MHC I	LPFDKSTIM	MHC tetramer/multimer staining	HLA-B*3501	NP	Influenza A virus (A/Texas/1/1977(H3N2))
674	14764717	neg	MHC I	LPFDKSTIM	MHC tetramer/multimer staining	HLA-B*3501	NP	Influenza A virus (A/Texas/1/1977(H3N2))
1177	15113903	neg	MHC I	SGYWAIRTR	intracellular cytokine staining (ICS)	HLA-B*2705	NP	Influenza A virus (A/Netherlands/018/94 (H3N2))
1000374	15163496	pos	MHC I	SRYWAIRTR	51 chromium release	HLA-B*2705	NP	Influenza A virus (A/Hong Kong/2/68(H3N2))

1000374	15163496	pos	MHC I	SRYWAIRTR	51 chromium release	HLA-B*2705	NP	Influenza A virus (A/Hong Kong/2/68(H3N2))
1000374	15163496	pos	MHC I	LPFDKTTIM	51 chromium release	HLA-B*3501	NP	Influenza A virus (A/Hong Kong/2/68(H3N2))
1000374	15163496	pos	MHC I	LPFDKTTIM	51 chromium release	HLA-B*3501	NP	Influenza A virus (A/Hong Kong/2/68(H3N2))
1000374	15163496	pos	MHC I	ELRSRYWAI	51 chromium release	HLA-B8	NP	Influenza A virus (A/Hong Kong/2/68(H3N2))
1000374	15163496	pos	MHC I	ELRSRYWAI	51 chromium release	HLA-B8	NP	Influenza A virus (A/Hong Kong/2/68(H3N2))
1000158	16054612	pos	MHC I	RRATAILRK	ELISPOT	HLA-B*2705	PB2	Influenza A virus (A/X-31(H3N2))
1000158	16054612	pos	MHC I	KRYGPALSI	ELISPOT	HLA-B*2705	PB2	Influenza A virus (A/X-31(H3N2))
1000158	16054612	pos	MHC I	RRSFEIKKL	ELISPOT	HLA-B*2705	PB1	Influenza A virus (A/X-31(H3N2))
1003542	17251571	pos	MHC I	AEIEDLIFS	ELISPOT	HLA-B*4002	NP	Influenza A virus (A/Bilthoven/4791/81(H3N2))
1003542	17251571	pos	MHC I	AEIEDLIFS	51 chromium release	HLA-B*4002	NP	Influenza A virus (A/Bilthoven/4791/81(H3N2))
1003542	17251571	pos	MHC I	AEIEDLIFS	ELISPOT	HLA-B*4002	NP	Influenza A virus (A/Bilthoven/4791/81(H3N2))
1003542	17251571	pos	MHC I	AEIEDLIFL	ELISPOT	HLA-B*4002	NP	Influenza A virus H3N2 (A/Netherlands/9/03 (H3N2))
1003542	17251571	pos	MHC I	AEIEDLIFL	51 chromium release	HLA-B*4002	NP	Influenza A virus H3N2 (A/Netherlands/9/03 (H3N2))
1003542	17251571	pos	MHC I	AEIEDLIFL	ELISPOT	HLA-B*4002	NP	Influenza A virus H3N2 (A/Netherlands/9/03 (H3N2))
1003542	17251571	pos	MHC I	AEIEDLIFL	51 chromium release	HLA-B*4002	NP	Influenza A virus H3N2 (A/Netherlands/9/03 (H3N2))
1007681	17507491	pos	MHC II	TYADTICIGYHANNSTDT	ELISPOT	HLA-DR1	HA	Influenza A virus (A/New Caledonia/20/1999(H1N1))
1007681	17507491	pos	MHC II	VLEKNVTVTHSVNLLEDS	ELISPOT	HLA-DR1	HA	Influenza A virus (A/New Caledonia/20/1999(H1N1))
1007681	17507491	pos	MHC II	VTHSVNLLEDSHNGKLCL	ELISPOT	HLA-DR1	HA	Influenza A virus (A/New Caledonia/20/1999(H1N1))
1007681	17507491	pos	MHC II	KLCLLKGIAPLQLGNCSV	ELISPOT	HLA-DR1	HA	Influenza A virus (A/New Caledonia/20/1999(H1N1))
1007681	17507491	pos	MHC II	IAPLQLGNCSVAGWILGN	ELISPOT	HLA-DR1	HA	Influenza A virus (A/New Caledonia/20/1999(H1N1))
1007681	17507491	pos	MHC II	NCSVAGWILGNPECELLI	ELISPOT	HLA-DR1	HA	Influenza A virus (A/New Caledonia/20/1999(H1N1))
1007681	17507491	pos	MHC II	ELLISKESWSYIVETPNP	ELISPOT	HLA-DR1	HA	Influenza A virus (A/New Caledonia/20/1999(H1N1))
1007681	17507491	pos	MHC II	SWSYIVETPNPENGTCYP	ELISPOT	HLA-DR1	HA	Influenza A virus (A/New Caledonia/20/1999(H1N1))
1007681	17507491	pos	MHC II	TPNPENGTCYPGYFADYE	ELISPOT	HLA-DR1	HA	Influenza A virus (A/New Caledonia/20/1999(H1N1))
1007681	17507491	pos	MHC II	TCYPGYFADYEELREQLS	ELISPOT	HLA-DR1	HA	Influenza A virus (A/New Caledonia/20/1999(H1N1))
1007681	17507491	pos	MHC II	EQLSSVSSFERFEIFPKE	ELISPOT	HLA-DR1	HA	Influenza A virus (A/New Caledonia/20/1999(H1N1))
1007681	17507491	pos	MHC II	SFERFEIFPKESSWPNHT	ELISPOT	HLA-DR1	HA	Influenza A virus (A/New Caledonia/20/1999(H1N1))
1007681	17507491	pos	MHC II	FPKESSWPNHTVTGVSAS	ELISPOT	HLA-DR1	HA	Influenza A virus (A/New Caledonia/20/1999(H1N1))
1007681	17507491	pos	MHC II	NGKSSFYRNLLWLTGKNG	ELISPOT	HLA-DR1	HA	Influenza A virus (A/New Caledonia/20/1999(H1N1))
1007681	17507491	pos	MHC II	RNLLWLTGKNGLYPNLSK	ELISPOT	HLA-DR1	HA	Influenza A virus (A/New Caledonia/20/1999(H1N1))
1007681	17507491	pos	MHC II	NNKEKEVLVLWGVHHPPN	ELISPOT	HLA-DR1	HA	Influenza A virus (A/New Caledonia/20/1999(H1N1))
1007681	17507491	pos	MHC II	HPPNIGNQRALYHTENAY	ELISPOT	HLA-DR1	HA	Influenza A virus (A/New Caledonia/20/1999(H1N1))
1007681	17507491	pos	MHC II	QRALYHTENAYVSVVSSH	ELISPOT	HLA-DR1	HA	Influenza A virus (A/New Caledonia/20/1999(H1N1))
1007681	17507491	pos	MHC II	GDTIIFEANGNLIAPWYA	ELISPOT	HLA-DR1	HA	Influenza A virus (A/New Caledonia/20/1999(H1N1))
1007681	17507491	pos	MHC II	ANGNLIAPWYAFALSRGF	ELISPOT	HLA-DR1	HA	Influenza A virus (A/New Caledonia/20/1999(H1N1))
1007681	17507491	pos	MHC II	MDECDAKCQTPQGAINSS	ELISPOT	HLA-DR1	HA	Influenza A virus (A/New Caledonia/20/1999(H1N1))
1007681	17507491	pos	MHC II	CQTPQGAINSSLPFQNVH	ELISPOT	HLA-DR1	HA	Influenza A virus (A/New Caledonia/20/1999(H1N1))
1007681	17507491	pos	MHC II	QNVHPVTIGECPKYVRSA	ELISPOT	HLA-DR1	HA	Influenza A virus (A/New Caledonia/20/1999(H1N1))
1007681	17507491	pos	MHC II	IGECPKYVRSAKLRMVTG	ELISPOT	HLA-DR1	HA	Influenza A virus (A/New Caledonia/20/1999(H1N1))

1007681	17507491	pos	MHC II	VRSAKLRMVTGLRNIPSI	ELISPOT	HLA-DR1	HA	Influenza A virus (A/New Caledonia/20/1999(H1N1))
1007681	17507491	pos	MHC II	TGMVDGWYGYHHQNEQGS	ELISPOT	HLA-DR1	HA	Influenza A virus (A/New Caledonia/20/1999(H1N1))
1007681	17507491	pos	MHC II	NKVNSVIEKMNTQFTAVG	ELISPOT	HLA-DR1	HA	Influenza A virus (A/New Caledonia/20/1999(H1N1))
1007681	17507491	pos	MHC II	WTYNAELLVLLENERTLD	ELISPOT	HLA-DR1	HA	Influenza A virus (A/New Caledonia/20/1999(H1N1))
1007681	17507491	pos	MHC II	LVLLENERTLDFHDSNVK	ELISPOT	HLA-DR1	HA	Influenza A virus (A/New Caledonia/20/1999(H1N1))
1007681	17507491	pos	MHC II	YHKCNNECMESVKNGTYD	ELISPOT	HLA-DR1	HA	Influenza A virus (A/New Caledonia/20/1999(H1N1))
1007681	17507491	neg	MHC II	MKAKLLVLLCTFTATYAD	ELISPOT	HLA-DR1	HA	Influenza A virus (A/New Caledonia/20/1999(H1N1))
1007681	17507491	neg	MHC II	LLCTFTATYADTICIGYH	ELISPOT	HLA-DR1	HA	Influenza A virus (A/New Caledonia/20/1999(H1N1))
1007681	17507491	neg	MHC II	IGYHANNSTDTVDTVLEK	ELISPOT	HLA-DR1	HA	Influenza A virus (A/New Caledonia/20/1999(H1N1))
1007681	17507491	neg	MHC II	ILGNPECELLISKESWSY	ELISPOT	HLA-DR1	HA	Influenza A virus (A/New Caledonia/20/1999(H1N1))
1007681	17507491	neg	MHC II	ADYEELREQLSSVSSFER	ELISPOT	HLA-DR1	HA	Influenza A virus (A/New Caledonia/20/1999(H1N1))
1007681	17507491	neg	MHC II	PNHTVTGVSASCSHNGKS	ELISPOT	HLA-DR1	HA	Influenza A virus (A/New Caledonia/20/1999(H1N1))
1007681	17507491	neg	MHC II	VSASCSHNGKSSFYRNLL	ELISPOT	HLA-DR1	HA	Influenza A virus (A/New Caledonia/20/1999(H1N1))
1007681	17507491	neg	MHC II	GKNGLYPNLSKSYVNNKE	ELISPOT	HLA-DR1	HA	Influenza A virus (A/New Caledonia/20/1999(H1N1))
1007681	17507491	neg	MHC II	NLSKSYVNNKEKEVLVLW	ELISPOT	HLA-DR1	HA	Influenza A virus (A/New Caledonia/20/1999(H1N1))
1007681	17507491	neg	MHC II	LVLWGVHHPPNIGNQRAL	ELISPOT	HLA-DR1	HA	Influenza A virus (A/New Caledonia/20/1999(H1N1))
1007681	17507491	neg	MHC II	ENAYVSVVSSHYSRRFTP	ELISPOT	HLA-DR1	HA	Influenza A virus (A/New Caledonia/20/1999(H1N1))
1007681	17507491	neg	MHC II	KRPKVRDQEGRINYYWTL	ELISPOT	HLA-DR1	HA	Influenza A virus (A/New Caledonia/20/1999(H1N1))
1007681	17507491	neg	MHC II	QEGRINYYWTLLEPGDTI	ELISPOT	HLA-DR1	HA	Influenza A virus (A/New Caledonia/20/1999(H1N1))
1007681	17507491	neg	MHC II	YWTLLEPGDTIIFEANGN	ELISPOT	HLA-DR1	HA	Influenza A virus (A/New Caledonia/20/1999(H1N1))
1007681	17507491	neg	MHC II	PWYAFALSRGFGSGIITS	ELISPOT	HLA-DR1	HA	Influenza A virus (A/New Caledonia/20/1999(H1N1))
1007681	17507491	neg	MHC II	IITSNAPMDECDAKCQTP	ELISPOT	HLA-DR1	HA	Influenza A virus (A/New Caledonia/20/1999(H1N1))
1007681	17507491	neg	MHC II	INSSLPFQNVHPVTIGEC	ELISPOT	HLA-DR1	HA	Influenza A virus (A/New Caledonia/20/1999(H1N1))
1007681	17507491	neg	MHC II	MVTGLRNIPSIQSRGLFG	ELISPOT	HLA-DR1	HA	Influenza A virus (A/New Caledonia/20/1999(H1N1))
1007681	17507491	neg	MHC II	IPSIQSRGLFGAIAGFIE	ELISPOT	HLA-DR1	HA	Influenza A virus (A/New Caledonia/20/1999(H1N1))
1007681	17507491	neg	MHC II	GLFGAIAGFIEGGWTGMV	ELISPOT	HLA-DR1	HA	Influenza A virus (A/New Caledonia/20/1999(H1N1))
1007681	17507491	neg	MHC II	GFIEGGWTGMVDGWYGYH	ELISPOT	HLA-DR1	HA	Influenza A virus (A/New Caledonia/20/1999(H1N1))
1007681	17507491	neg	MHC II	YGYHHQNEQGSGYAADQK	ELISPOT	HLA-DR1	HA	Influenza A virus (A/New Caledonia/20/1999(H1N1))
1007681	17507491	neg	MHC II	NAINGITNKVNSVIEKMN	ELISPOT	HLA-DR1	HA	Influenza A virus (A/New Caledonia/20/1999(H1N1))
1007681	17507491	neg	MHC II	EKMNTQFTAVGKEFNKLE	ELISPOT	HLA-DR1	HA	Influenza A virus (A/New Caledonia/20/1999(H1N1))
1007681	17507491	neg	MHC II	TAVGKEFNKLERRMENLN	ELISPOT	HLA-DR1	HA	Influenza A virus (A/New Caledonia/20/1999(H1N1))
1007681	17507491	neg	MHC II	NKLERRMENLNKKVDDGF	ELISPOT	HLA-DR1	HA	Influenza A virus (A/New Caledonia/20/1999(H1N1))
1007681	17507491	neg	MHC II	ENLNKKVDDGFLDIWTYN	ELISPOT	HLA-DR1	HA	Influenza A virus (A/New Caledonia/20/1999(H1N1))
1007681	17507491	neg	MHC II	DDGFLDIWTYNAELLVLL	ELISPOT	HLA-DR1	HA	Influenza A virus (A/New Caledonia/20/1999(H1N1))
1007681	17507491	neg	MHC II	RTLDFHDSNVKNLYEKVK	ELISPOT	HLA-DR1	HA	Influenza A virus (A/New Caledonia/20/1999(H1N1))
1007681	17507491	neg	MHC II	SNVKNLYEKVKSQLKNNA	ELISPOT	HLA-DR1	HA	Influenza A virus (A/New Caledonia/20/1999(H1N1))
1007681	17507491	neg	MHC II	EKVKSQLKNNAKEIGNGC	ELISPOT	HLA-DR1	HA	Influenza A virus (A/New Caledonia/20/1999(H1N1))
1007681	17507491	neg	MHC II	KNNAKEIGNGCFEFYHKC	ELISPOT	HLA-DR1	HA	Influenza A virus (A/New Caledonia/20/1999(H1N1))
1007681	17507491	neg	MHC II	GNGCFEFYHKCNNECMES	ELISPOT	HLA-DR1	HA	Influenza A virus (A/New Caledonia/20/1999(H1N1))

1007681	17507491	neg	MHC II	CMESVKNGTYDYPKYSEE	ELISPOT	HLA-DR1	HA	Influenza A virus (A/New Caledonia/20/1999(H1N1))
1007681	17507491	neg	MHC II	GTYDYPKYSEESKLNREK	ELISPOT	HLA-DR1	HA	Influenza A virus (A/New Caledonia/20/1999(H1N1))
1007681	17507491	neg	MHC II	NREKIDGVKLESMGVYQI	ELISPOT	HLA-DR1	HA	Influenza A virus (A/New Caledonia/20/1999(H1N1))
1007681	17507491	neg	MHC II	VKLESMGVYQILAIYSTV	ELISPOT	HLA-DR1	HA	Influenza A virus (A/New Caledonia/20/1999(H1N1))
1007681	17507491	neg	MHC II	YSTVASSLVLLVSLGAIS	ELISPOT	HLA-DR1	HA	Influenza A virus (A/New Caledonia/20/1999(H1N1))
1007681	17507491	neg	MHC II	GAISFWMCSNGSLQCRIC	ELISPOT	HLA-DR1	HA	Influenza A virus (A/New Caledonia/20/1999(H1N1))
1009685	18209073	pos	MHC II	ELLVLMENERTLDFHD	MHC tetramer/multimer staining	HLA-DRB1*0101	HA	Influenza A virus (A/Viet Nam/1203/2004(H5N1))
1009685	18209073	pos	MHC II	ELLVLMENERTLDFHD	intracellular cytokine staining (ICS)	HLA-DRB1*0101	HA	Influenza A virus (A/Viet Nam/1203/2004(H5N1))
1009685	18209073	pos	MHC II	ELLVLMENERTLDFHD	intracellular cytokine staining (ICS)	HLA-DRB1*0101	HA	Influenza A virus (A/Viet Nam/1203/2004(H5N1))
1009685	18209073	pos	MHC II	SGPLKAEIAQKLEDVFAGKN	MHC tetramer/multimer staining	HLA-DRB1*0101	M1	Influenza A virus (A/Viet Nam/1203/2004(H5N1))
1009685	18209073	pos	MHC II	SHLECRTFFLTQGALLNDKH	MHC tetramer/multimer staining	HLA-DRB1*0101	NA	Influenza A virus (A/Viet Nam/1203/2004(H5N1))
1009685	18209073	pos	MHC II	RMVLSAFDERRNRYLEEHPS	MHC tetramer/multimer staining	HLA-DRB1*0101	NP	Influenza A virus (A/Viet Nam/1203/2004(H5N1))
1009685	18209073	pos	MHC II	ERRNRYLEEHPSAGKDPKKT	MHC tetramer/multimer staining	HLA-DRB1*0101	NP	Influenza A virus (A/Viet Nam/1203/2004(H5N1))
1009685	18209073	pos	MHC II	PRMCSLMQGSTLPRRSGAAG	MHC tetramer/multimer staining	HLA-DRB1*0101	NP	Influenza A virus (A/Viet Nam/1203/2004(H5N1))
1009685	18209073	pos	MHC II	GVGTMVMELIRMIKRGINDR	MHC tetramer/multimer staining	HLA-DRB1*0101	NP	Influenza A virus (A/Viet Nam/1203/2004(H5N1))
1009685	18209073	pos	MHC II	SLVGIDPFRLLQNSQVFSLI	MHC tetramer/multimer staining	HLA-DRB1*0101	NP	Influenza A virus (A/Viet Nam/1203/2004(H5N1))
1009685	18209073	pos	MHC II	ASAGQISVQPTFSVQRNLPF	MHC tetramer/multimer staining	HLA-DRB1*0101	NP	Influenza A virus (A/Viet Nam/1203/2004(H5N1))
1009685	18209073	pos	MHC II	RTEIIRMMESARPEDVSFQG	MHC tetramer/multimer staining	HLA-DRB1*0101	NP	Influenza A virus (A/Viet Nam/1203/2004(H5N1))
1009685	18209073	pos	MHC II	GVTNKVNSIIDKMNTQFEAV	MHC tetramer/multimer staining	HLA-DRB1*0301	HA	Influenza A virus (A/Viet Nam/1194/2004(H5N1))
1009685	18209073	pos	MHC II	PTIKRSYNNTNQEDL	MHC tetramer/multimer staining	HLA-DRB1*0404	HA	Influenza A virus (A/Viet Nam/1203/2004(H5N1))
1009685	18209073	pos	MHC II	ELLVLMENERTLDFHD	MHC tetramer/multimer staining	HLA-DRB1*0404	HA	Influenza A virus (A/Viet Nam/1203/2004(H5N1))
1009685	18209073	pos	MHC II	TLTVPSERGLQRRRFVQNAL	MHC tetramer/multimer staining	HLA-DRB1*0404	M1	Influenza A virus (A/Viet Nam/1203/2004(H5N1))
1009685	18209073	pos	MHC II	GLQRRRFVQNALNGNGDPNN	MHC tetramer/multimer staining	HLA-DRB1*0404	M1	Influenza A virus (A/Viet Nam/1203/2004(H5N1))
1009685	18209073	pos	MHC II	VKLYKKLKREITFHGAKEVA	MHC tetramer/multimer staining	HLA-DRB1*0404	M1	Influenza A virus (A/Viet Nam/1203/2004(H5N1))
1009685	18209073	pos	MHC II	TGALASCMGLIYNRMGTVTT	MHC tetramer/multimer staining	HLA-DRB1*0404	M1	Influenza A virus (A/Viet Nam/1203/2004(H5N1))
1009685	18209073	pos	MHC II	EAMEIANQARQMVQAMRTIG	MHC tetramer/multimer staining	HLA-DRB1*0404	M1	Influenza A virus (A/Viet Nam/1203/2004(H5N1))
1009685	18209073	pos	MHC II	ARQMVQAMRTIGTHPNSSAG	MHC tetramer/multimer staining	HLA-DRB1*0404	M1	Influenza A virus (A/Viet Nam/1203/2004(H5N1))
1009685	18209073	pos	MHC II	NNILRTQESECACVNGSCFT	MHC tetramer/multimer staining	HLA-DRB1*0404	NA	Influenza A virus (A/Viet Nam/1203/2004(H5N1))
1009685	18209073	pos	MHC II	TNSRSGFEMIWDPNGWTETD	MHC tetramer/multimer staining	HLA-DRB1*0404	NA	Influenza A virus (A/Viet Nam/1203/2004(H5N1))

1009685	18209073	pos	MHC II	GRPKESTIWTSGSSISFCGV	MHC tetramer/multimer staining	HLA-DRB1*0404	NA	Influenza A virus (A/Viet Nam/1203/2004(H5N1))
1009685	18209073	pos	MHC II	NPAHKSQLVWMACHSAAFED	MHC tetramer/multimer staining	HLA-DRB1*0404	NP	Influenza A virus (A/Viet Nam/1203/2004(H5N1))
1009685	18209073	pos	MHC II	ASAGQISVQPTFSVQRNLPF	MHC tetramer/multimer staining	HLA-DRB1*0404	NP	Influenza A virus (A/Viet Nam/1203/2004(H5N1))
1009685	18209073	pos	MHC II	TEGRTSDMRTEIIRMMESAR	MHC tetramer/multimer staining	HLA-DRB1*0404	NP	Influenza A virus (A/Viet Nam/1203/2004(H5N1))
1009685	18209073	pos	MHC II	RTEIIRMMESARPEDVSFQG	MHC tetramer/multimer staining	HLA-DRB1*0404	NP	Influenza A virus (A/Viet Nam/1203/2004(H5N1))
1009685	18209073	pos	MHC II	LEKKHNGKLCDLDGVKPLIL	MHC tetramer/multimer staining	HLA-DRB1*0701	HA	Influenza A virus (A/Viet Nam/1203/2004(H5N1))
1009685	18209073	pos	MHC II	ELLVLMENERTLDFHD	intracellular cytokine staining (ICS)	HLA-DRB1*0701	HA	Influenza A virus (A/Viet Nam/1203/2004(H5N1))
1009685	18209073	pos	MHC II	ELLVLMENERTLDFHD	intracellular cytokine staining (ICS)	HLA-DRB1*0701	HA	Influenza A virus (A/Viet Nam/1203/2004(H5N1))
1009685	18209073	pos	MHC II	TYVLSIIPSGPLKAEIAQKL	MHC tetramer/multimer staining	HLA-DRB1*0701	M1	Influenza A virus (A/Viet Nam/1203/2004(H5N1))
1009685	18209073	pos	MHC II	AGKNTDLEALMEWLKTRPIL	MHC tetramer/multimer staining	HLA-DRB1*0701	M1	Influenza A virus (A/Viet Nam/1203/2004(H5N1))
1009685	18209073	pos	MHC II	ALMEWLKTRPILSPLTKGIL	MHC tetramer/multimer staining	HLA-DRB1*0701	M1	Influenza A virus (A/Viet Nam/1203/2004(H5N1))
1009685	18209073	pos	MHC II	VKLYKKLKREITFHGAKEVA	MHC tetramer/multimer staining	HLA-DRB1*0701	M1	Influenza A virus (A/Viet Nam/1203/2004(H5N1))
1009685	18209073	pos	MHC II	SHRQMATITNPLIRHENRMV	MHC tetramer/multimer staining	HLA-DRB1*0701	M1	Influenza A virus (A/Viet Nam/1203/2004(H5N1))
1009685	18209073	pos	MHC II	TNPLIRHENRMVLASTTAKA	MHC tetramer/multimer staining	HLA-DRB1*0701	M1	Influenza A virus (A/Viet Nam/1203/2004(H5N1))
1009685	18209073	pos	MHC II	NRMVLASTTAKAMEQMAGSS	MHC tetramer/multimer staining	HLA-DRB1*0701	M1	Influenza A virus (A/Viet Nam/1203/2004(H5N1))
1009685	18209073	pos	MHC II	PINGWAVYSKDNSIRIGSKG	MHC tetramer/multimer staining	HLA-DRB1*0701	NA	Influenza A virus (A/Viet Nam/1203/2004(H5N1))
1009685	18209073	pos	MHC II	PINGWAVYSKDNSIRIGSKG	intracellular cytokine staining (ICS)	HLA-DRB1*0701	NA	Influenza A virus (A/Viet Nam/1203/2004(H5N1))
1009685	18209073	pos	MHC II	PINGWAVYSKDNSIRIGSKG	intracellular cytokine staining (ICS)	HLA-DRB1*0701	NA	Influenza A virus (A/Viet Nam/1203/2004(H5N1))
1009685	18209073	pos	MHC II	SHLECRTFFLTQGALLNDKH	MHC tetramer/multimer staining	HLA-DRB1*0701	NA	Influenza A virus (A/Viet Nam/1203/2004(H5N1))
1009685	18209073	pos	MHC II	SHLECRTFFLTQGALLNDKH	intracellular cytokine staining (ICS)	HLA-DRB1*0701	NA	Influenza A virus (A/Viet Nam/1203/2004(H5N1))
1009685	18209073	pos	MHC II	SHLECRTFFLTQGALLNDKH	intracellular cytokine staining (ICS)	HLA-DRB1*0701	NA	Influenza A virus (A/Viet Nam/1203/2004(H5N1))
1009685	18209073	pos	MHC II	SHLECRTFFLTQGALLNDKH	intracellular cytokine staining (ICS)	HLA-DRB1*0701	NA	Influenza A virus (A/Viet Nam/1203/2004(H5N1))
1009685	18209073	pos	MHC II	SHLECRTFFLTQGALLNDKH	intracellular cytokine staining (ICS)	HLA-DRB1*0701	NA	Influenza A virus (A/Viet Nam/1203/2004(H5N1))
1009685	18209073	pos	MHC II	SHLECRTFFLTQGALLNDKH	intracellular cytokine staining (ICS)	HLA-DRB1*0701	NA	Influenza A virus (A/Viet Nam/1203/2004(H5N1))
1009685	18209073	pos	MHC II	SHLECRTFFLTQGALLNDKH	intracellular cytokine staining (ICS)	HLA-DRB1*0701	NA	Influenza A virus (A/Viet Nam/1203/2004(H5N1))
1009685	18209073	pos	MHC II	FLTQGALLNDKHSNGTVKDR	MHC tetramer/multimer staining	HLA-DRB1*0701	NA	Influenza A virus (A/Viet Nam/1203/2004(H5N1))
1009685	18209073	pos	MHC II	MIWHSNLNDATYQRTRALVR	MHC tetramer/multimer staining	HLA-DRB1*0701	NP	Influenza A virus (A/Viet Nam/1203/2004(H5N1))

1009685	18209073	pos	MHC II	DATYQRTRALVRTGMDPRMC	MHC tetramer/multimer staining	HLA-DRB1*0701	NP	Influenza A virus (A/Viet Nam/1203/2004(H5N1))
1009685	18209073	pos	MHC II	IFLARSALILRGSVAHKSCL	MHC tetramer/multimer staining	HLA-DRB1*0701	NP	Influenza A virus (A/Viet Nam/1203/2004(H5N1))
1009685	18209073	pos	MHC II	ILRGSVAHKSCLPACVYGLA	MHC tetramer/multimer staining	HLA-DRB1*0701	NP	Influenza A virus (A/Viet Nam/1203/2004(H5N1))
1009685	18209073	pos	MHC II	NPAHKSQLVWMACHSAAFED	MHC tetramer/multimer staining	HLA-DRB1*0701	NP	Influenza A virus (A/Viet Nam/1203/2004(H5N1))
1009685	18209073	pos	MHC II	VKLYKKLKREITFHGAKEVA	MHC tetramer/multimer staining	HLA-DRB1*1101	M1	Influenza A virus (A/Viet Nam/1203/2004(H5N1))
1009685	18209073	pos	MHC II	TNPLIRHENRMVLASTTAKA	MHC tetramer/multimer staining	HLA-DRB1*1101	M1	Influenza A virus (A/Viet Nam/1203/2004(H5N1))
1009685	18209073	pos	MHC II	EAMEIANQARQMVQAMRTIG	MHC tetramer/multimer staining	HLA-DRB1*1101	M1	Influenza A virus (A/Viet Nam/1203/2004(H5N1))
1009685	18209073	pos	MHC II	ARQMVQAMRTIGTHPNSSAG	MHC tetramer/multimer staining	HLA-DRB1*1101	M1	Influenza A virus (A/Viet Nam/1203/2004(H5N1))
1009685	18209073	pos	MHC II	GPSNGQASHKIFKMEKGKVV	MHC tetramer/multimer staining	HLA-DRB1*1101	NA	Influenza A virus (A/Viet Nam/1203/2004(H5N1))
1009685	18209073	pos	MHC II	HKIFKMEKGKVVKSVELDAP	MHC tetramer/multimer staining	HLA-DRB1*1101	NA	Influenza A virus (A/Viet Nam/1203/2004(H5N1))
1009685	18209073	pos	MHC II	TGLDCIRPCFWVELIRGRPK	MHC tetramer/multimer staining	HLA-DRB1*1101	NA	Influenza A virus (A/Viet Nam/1203/2004(H5N1))
1009685	18209073	pos	MHC II	CFWVELIRGRPKESTIWTSG	MHC tetramer/multimer staining	HLA-DRB1*1101	NA	Influenza A virus (A/Viet Nam/1203/2004(H5N1))
1009685	18209073	pos	MHC II	VRELILYDKEEIRRIWRQAN	MHC tetramer/multimer staining	HLA-DRB1*1101	NP	Influenza A virus (A/Viet Nam/1203/2004(H5N1))
1009685	18209073	pos	MHC II	KEEIRRIWRQANNGEDATAG	MHC tetramer/multimer staining	HLA-DRB1*1101	NP	Influenza A virus (A/Viet Nam/1203/2004(H5N1))
1009685	18209073	pos	MHC II	GVGTMVMELIRMIKRGINDR	MHC tetramer/multimer staining	HLA-DRB1*1101	NP	Influenza A virus (A/Viet Nam/1203/2004(H5N1))
1009685	18209073	pos	MHC II	LIRMIKRGINDRNFWRGENG	MHC tetramer/multimer staining	HLA-DRB1*1101	NP	Influenza A virus (A/Viet Nam/1203/2004(H5N1))
1009685	18209073	pos	MHC II	NTLELRSRYWAIRTRSGGNT	MHC tetramer/multimer staining	HLA-DRB1*1101	NP	Influenza A virus (A/Viet Nam/1203/2004(H5N1))
1009685	18209073	pos	MHC II	GFLDVWTYNAELLVLMENER	MHC tetramer/multimer staining	HLA-DRB1*1501	HA	Influenza A virus (A/Viet Nam/1194/2004(H5N1))
1012379	18353950	pos	MHC I	GILGFVFTL	ELISPOT	HLA-A*0201	M1	H5N1 subtype
1012379	18353950	pos	MHC I	GMLGFVFTL	ELISPOT	HLA-A*0201	M1	H5N1 subtype
1012379	18353950	pos	MHC I	AIMDKNIIL	ELISPOT	HLA-A*0201	NS1	H5N1 subtype
1012379	18353950	pos	MHC I	CTELKLSDY	ELISPOT	HLA-A1	NP	H5N1 subtype
1012379	18353950	pos	MHC I	ILRGSVAHK	ELISPOT	HLA-A3	NP	H5N1 subtype
1012379	18353950	pos	MHC I	ILRGSIAHK	ELISPOT	HLA-A3	NP	H5N1 subtype
1012379	18353950	pos	MHC I	RRSGAAGAAVK	ELISPOT	HLA-B*2705	NP	H5N1 subtype
1012379	18353950	pos	MHC I	RRSGAAGAAIK	ELISPOT	HLA-B*2705	NP	H5N1 subtype
1012379	18353950	pos	MHC I	LPFEKSTVM	ELISPOT	HLA-B*3501	NP	H5N1 subtype
1012379	18353950	pos	MHC I	LPFERATIM	ELISPOT	HLA-B*3501	NP	H5N1 subtype
1012379	18353950	pos	MHC I	LPFERSTIM	ELISPOT	HLA-B*3501	NP	H5N1 subtype
1013468	18614638	pos	MHC I	GILGFVFTL	ELISPOT	HLA-A*0201	M1	Influenza A virus (A/Puerto Rico/8/1934(H1N1))
1013468	18614638	pos	MHC I	GILGFVFTL	51 chromium release	HLA-A*0201	M1	Influenza A virus (A/Puerto Rico/8/1934(H1N1))

1013468	18614638	pos	MHC I	GILGFVFTL	51 chromium release	HLA-A*0201	M1	Influenza A virus (A/Puerto Rico/8/1934(H1N1))
1013468	18614638	pos	MHC I	SGPLKAEIAQRLEDV	51 chromium release	HLA-A*0201	M1	Influenza A virus (A/Puerto Rico/8/1934(H1N1))
1013468	18614638	pos	MHC I	SGPLKAEIAQRLEDV	51 chromium release	HLA-A*0201	M1	Influenza A virus (A/Puerto Rico/8/1934(H1N1))
1013468	18614638	pos	MHC I	AIMDKNIIL	51 chromium release	HLA-A*0201	NS1	Influenza A virus (A/Puerto Rico/8/1934(H1N1))
1013468	18614638	pos	MHC I	AIMDKNIIL	51 chromium release	HLA-A*0201	NS1	Influenza A virus (A/Puerto Rico/8/1934(H1N1))
1013935	19122146	pos	MHC I	LPFDRTTVM	ELISPOT	HLA-B*0702	NP	Influenza A virus (A/Puerto Rico/8/1934(H1N1))
1013935	19122146	pos	MHC I	SPIVPSFDM	ELISPOT	HLA-B*0702	NP	Influenza A virus (A/Puerto Rico/8/1934(H1N1))
1013935	19122146	pos	MHC I	QPEWFRNVL	ELISPOT	HLA-B*0702	PB1	Influenza A virus (A/Puerto Rico/8/1934(H1N1))
1019632	19386707	pos	MHC II	VTHSVNLLEDSHNGKL	ELISPOT	HLA-DR1	HA	Influenza A virus (A/New Caledonia/20/1999(H1N1))
1019632	19386707	pos	MHC II	SSFERFEIFPKESSWPN	ELISPOT	HLA-DR1	HA	Influenza A virus (A/New Caledonia/20/1999(H1N1))
1019632	19386707	pos	MHC II	EIFPKESSWPNHTVTGV	ELISPOT	HLA-DR1	HA	Influenza A virus (A/New Caledonia/20/1999(H1N1))
1019632	19386707	pos	MHC II	GKSSFYRNLLWLTGKNG	ELISPOT	HLA-DR1	HA	Influenza A virus (A/New Caledonia/20/1999(H1N1))
1019632	19386707	pos	MHC II	RNLLWLTGKNGLYPNLS	ELISPOT	HLA-DR1	HA	Influenza A virus (A/New Caledonia/20/1999(H1N1))
1019632	19386707	pos	MHC II	NQRALYHTENAYVSVVS	ELISPOT	HLA-DR1	HA	Influenza A virus (A/New Caledonia/20/1999(H1N1))
1019632	19386707	pos	MHC II	LEPGDTIIFEANGNLIA	ELISPOT	HLA-DR1	HA	Influenza A virus (A/New Caledonia/20/1999(H1N1))
1019632	19386707	pos	MHC II	IIFEANGNLIAPWYAFA	ELISPOT	HLA-DR1	HA	Influenza A virus (A/New Caledonia/20/1999(H1N1))
1019632	19386707	pos	MHC II	GNLIAPWYAFALSRGFG	ELISPOT	HLA-DR1	HA	Influenza A virus (A/New Caledonia/20/1999(H1N1))
1019632	19386707	pos	MHC II	SGYAADQKSTQNAINGI	ELISPOT	HLA-DR1	HA	Influenza A virus (A/New Caledonia/20/1999(H1N1))
1019632	19386707	pos	MHC II	VIEKMNTQFTAVGKEFN	ELISPOT	HLA-DR1	HA	Influenza A virus (A/New Caledonia/20/1999(H1N1))
1019632	19386707	pos	MHC II	WTYNAELLVLLENERTLD	ELISPOT	HLA-DR1	HA	Influenza A virus (A/New Caledonia/20/1999(H1N1))
1019632	19386707	pos	MHC II	LVLLENERTLDFHDSNVK	ELISPOT	HLA-DR1	HA	Influenza A virus (A/New Caledonia/20/1999(H1N1))
1019632	19386707	pos	MHC II	ESMGVYQILAIYSTVAS	ELISPOT	HLA-DR1	HA	Influenza A virus (A/New Caledonia/20/1999(H1N1))
1019632	19386707	pos	MHC II	MNPNQKIITIGSISIAI	ELISPOT	HLA-DR1	NA	Influenza A virus (A/New Caledonia/20/1999(H1N1))
1019632	19386707	pos	MHC II	SSLCSISGWAIYTKDN	ELISPOT	HLA-DR1	NA	Influenza A virus (A/New Caledonia/20/1999(H1N1))
1019632	19386707	pos	MHC II	DVFVIREPFISCSHLE	ELISPOT	HLA-DR1	NA	Influenza A virus (A/New Caledonia/20/1999(H1N1))
1019632	19386707	pos	MHC II	RTFFLTQGALLNDKHSN	ELISPOT	HLA-DR1	NA	Influenza A virus (A/New Caledonia/20/1999(H1N1))
1019632	19386707	pos	MHC II	GTVKDRSPYRALMSCPL	ELISPOT	HLA-DR1	NA	Influenza A virus (A/New Caledonia/20/1999(H1N1))
1019632	19386707	pos	MHC II	KIEKGKVTKSIELNA	ELISPOT	HLA-DR1	NA	Influenza A virus (A/New Caledonia/20/1999(H1N1))
1019632	19386707	pos	MHC II	GKVTKSIELNAPNFHYE	ELISPOT	HLA-DR1	NA	Influenza A virus (A/New Caledonia/20/1999(H1N1))
1019632	19386707	pos	MHC II	WVSFNQNLDYQIGYICS	ELISPOT	HLA-DR1	NA	Influenza A virus (A/New Caledonia/20/1999(H1N1))
1019632	19386707	pos	MHC II	NLDYQIGYICSGVFGD	ELISPOT	HLA-DR1	NA	Influenza A virus (A/New Caledonia/20/1999(H1N1))
1019632	19386707	pos	MHC II	GVKGFSYKYGNGVWIGR	ELISPOT	HLA-DR1	NA	Influenza A virus (A/New Caledonia/20/1999(H1N1))
1019632	19386707	pos	MHC II	IRASVGRMIGGIGRFYI	ELISPOT	HLA-DR1	NP	Influenza A virus (A/New York/348/2003(H1N1))
1019632	19386707	pos	MHC II	ERRNKYLEEHPSAGKDP	ELISPOT	HLA-DR1	NP	Influenza A virus (A/New York/348/2003(H1N1))
1019632	19386707	pos	MHC II	RGINDRNFWRGENGRKT	ELISPOT	HLA-DR1	NP	Influenza A virus (A/New York/348/2003(H1N1))
1019632	19386707	pos	MHC II	NFWRGENGRKTRIAYER	ELISPOT	HLA-DR1	NP	Influenza A virus (A/New York/348/2003(H1N1))
1019632	19386707	pos	MHC II	EIEDLTFLARSALILRG	ELISPOT	HLA-DR1	NP	Influenza A virus (A/New York/348/2003(H1N1))
1019632	19386707	pos	MHC II	GVDPFKLLQTSQVYSLI	ELISPOT	HLA-DR1	NP	Influenza A virus (A/New York/348/2003(H1N1))
1019632	19386707	pos	MHC II	VYSLIRPNENPAHKSQL	ELISPOT	HLA-DR1	NP	Influenza A virus (A/New York/348/2003(H1N1))

1019632	19386707	pos	MHC II	HKSQLVWMACNSAAFED	ELISPOT	HLA-DR1	NP	Influenza A virus (A/New York/348/2003(H1N1))
1019632	19386707	pos	MHC II	TLELRSRYWAIRTRSGG	ELISPOT	HLA-DR1	NP	Influenza A virus (A/New York/348/2003(H1N1))
1019632	19386707	pos	MHC II	RYWAIRTRSGGNTNQQR	ELISPOT	HLA-DR1	NP	Influenza A virus (A/New York/348/2003(H1N1))
1019632	19386707	pos	MHC II	TNQQRASAGQISTQPTF	ELISPOT	HLA-DR1	NP	Influenza A virus (A/New York/348/2003(H1N1))
1019632	19386707	pos	MHC II	SAGQISTQPTFSVQRNL	ELISPOT	HLA-DR1	NP	Influenza A virus (A/New York/348/2003(H1N1))
1019632	19386707	pos	MHC II	SDMRAEIIKMMESARPE	ELISPOT	HLA-DR1	NP	Influenza A virus (A/New York/348/2003(H1N1))
1019632	19386707	pos	MHC II	IIKMMESARPEEVSFQG	ELISPOT	HLA-DR1	NP	Influenza A virus (A/New York/348/2003(H1N1))
1019632	19386707	pos	MHC II	ESDEAFKMTMASALASR	ELISPOT	HLA-DR1	NS1	Influenza A virus (A/New York/444/2001(H1N1))
1019632	19386707	pos	MHC II	KMTMASALASRYLTDMT	ELISPOT	HLA-DR1	NS1	Influenza A virus (A/New York/444/2001(H1N1))
1019632	19386707	pos	MHC II	ALASRYLTDMTIEEMSR	ELISPOT	HLA-DR1	NS1	Influenza A virus (A/New York/444/2001(H1N1))
1019632	19386707	pos	MHC II	WFMLMPKQKVAGPLCVR	ELISPOT	HLA-DR1	NS1	Influenza A virus (A/New York/444/2001(H1N1))
1019632	19386707	pos	MHC II	DQAIMDKNIILKANFSV	ELISPOT	HLA-DR1	NS1	Influenza A virus (A/New York/444/2001(H1N1))
1019632	19386707	pos	MHC II	KNIILKANFSVIFDRLE	ELISPOT	HLA-DR1	NS1	Influenza A virus (A/New York/444/2001(H1N1))
1019632	19386707	pos	MHC II	ANFSVIFDRLETLTLLR	ELISPOT	HLA-DR1	NS1	Influenza A virus (A/New York/444/2001(H1N1))
1019632	19386707	pos	MHC II	LTLLRAFTEEGAIVGEI	ELISPOT	HLA-DR1	NS1	Influenza A virus (A/New York/444/2001(H1N1))
1019632	19386707	pos	MHC II	FTEEGAIVGEISPLPSL	ELISPOT	HLA-DR1	NS1	Influenza A virus (A/New York/444/2001(H1N1))
1019632	19386707	pos	MHC II	VKNAIGVLIGGLEWNDN	ELISPOT	HLA-DR1	NS1	Influenza A virus (A/New York/444/2001(H1N1))
1019632	19386707	pos	MHC II	RSSNETGGPPFTPTQKR	ELISPOT	HLA-DR1	NS1	Influenza A virus (A/New York/444/2001(H1N1))
1019632	19386707	pos	MHC II	GGPPFTPTQKRKMAGTI	ELISPOT	HLA-DR1	NS1	Influenza A virus (A/New York/444/2001(H1N1))
1019632	19386707	pos	MHC II	PTQKRKMAGTIRSEV	ELISPOT	HLA-DR1	NS1	Influenza A virus (A/New York/444/2001(H1N1))
1019632	19386707	pos	MHC II	WTYNAELLVLMENERTLD	ELISPOT	HLA-DR1	HA	Influenza A virus (A/Vietnam/1203/2004(H5N1))
1019632	19386707	pos	MHC II	LVLMENERTLDFHDSNVK	ELISPOT	HLA-DR1	HA	Influenza A virus (A/Vietnam/1203/2004(H5N1))
1019632	19386707	pos	MHC II	MNPNKKIITIGSICMVT	ELISPOT	HLA-DR1	NA	Influenza A virus (A/Vietnam/1203/2004(H5N1))
1019632	19386707	pos	MHC II	SSLCPINGWAVYSKDN	ELISPOT	HLA-DR1	NA	Influenza A virus (A/Vietnam/1203/2004(H5N1))
1019632	19386707	pos	MHC II	KMEKGKVVKSVELDA	ELISPOT	HLA-DR1	NA	Influenza A virus (A/Vietnam/1203/2004(H5N1))
1019632	19386707	pos	MHC II	NLEYQIGYICSGVFGD	ELISPOT	HLA-DR1	NA	Influenza A virus (A/Vietnam/1203/2004(H5N1))
1019632	19386707	pos	MHC II	EMIWDPNGWTETDSSFS	ELISPOT	HLA-DR1	NA	Influenza A virus (A/Vietnam/1203/2004(H5N1))
1019632	19386707	neg	MHC II	DTVDTVLEKNVTVTHSV	ELISPOT	HLA-DR1	HA	Influenza A virus (A/New Caledonia/20/1999(H1N1))
1019632	19386707	neg	MHC II	NLLEDSHNGKLCLLKGI	ELISPOT	HLA-DR1	HA	Influenza A virus (A/New Caledonia/20/1999(H1N1))
1019632	19386707	neg	MHC II	LLKGIAPLQLGNCSVAG	ELISPOT	HLA-DR1	HA	Influenza A virus (A/New Caledonia/20/1999(H1N1))
1019632	19386707	neg	MHC II	CSVAGWILGNPECELLI	ELISPOT	HLA-DR1	HA	Influenza A virus (A/New Caledonia/20/1999(H1N1))
1019632	19386707	neg	MHC II	ILGNPECELLISKESWS	ELISPOT	HLA-DR1	HA	Influenza A virus (A/New Caledonia/20/1999(H1N1))
1019632	19386707	neg	MHC II	KESWSYIVETPNPENGT	ELISPOT	HLA-DR1	HA	Influenza A virus (A/New Caledonia/20/1999(H1N1))
1019632	19386707	neg	MHC II	EQLSSVSSFERFEIFPK	ELISPOT	HLA-DR1	HA	Influenza A virus (A/New Caledonia/20/1999(H1N1))
1019632	19386707	neg	MHC II	SYVNNKEKEVLVLWGVH	ELISPOT	HLA-DR1	HA	Influenza A virus (A/New Caledonia/20/1999(H1N1))
1019632	19386707	neg	MHC II	PPNIGNQRALYHTENAY	ELISPOT	HLA-DR1	HA	Influenza A virus (A/New Caledonia/20/1999(H1N1))
1019632	19386707	neg	MHC II	HTENAYVSVVSSHYSRR	ELISPOT	HLA-DR1	HA	Influenza A virus (A/New Caledonia/20/1999(H1N1))
1019632	19386707	neg	MHC II	HYSRRFTPEIAKRPKVR	ELISPOT	HLA-DR1	HA	Influenza A virus (A/New Caledonia/20/1999(H1N1))
1019632	19386707	neg	MHC II	TPEIAKRPKVRDQEGRI	ELISPOT	HLA-DR1	HA	Influenza A virus (A/New Caledonia/20/1999(H1N1))

1019632	19386707	neg	MHC II	SSLPFQNVHPVTIGECP	ELISPOT	HLA-DR1	HA	Influenza A virus (A/New Caledonia/20/1999(H1N1))
1019632	19386707	neg	MHC II	HQNEQGSGYAADQKSTQ	ELISPOT	HLA-DR1	HA	Influenza A virus (A/New Caledonia/20/1999(H1N1))
1019632	19386707	neg	MHC II	KYSEESKLNREKIDGVK	ELISPOT	HLA-DR1	HA	Influenza A virus (A/New Caledonia/20/1999(H1N1))
1019632	19386707	neg	MHC II	YQILAIYSTVASSLVLL	ELISPOT	HLA-DR1	HA	Influenza A virus (A/New Caledonia/20/1999(H1N1))
1019632	19386707	neg	MHC II	SLVLLVSLGAISFWMCS	ELISPOT	HLA-DR1	HA	Influenza A virus (A/New Caledonia/20/1999(H1N1))
1019632	19386707	neg	MHC II	TNVVAGKDKTSVTLAGN	ELISPOT	HLA-DR1	NA	Influenza A virus (A/New Caledonia/20/1999(H1N1))
1019632	19386707	neg	MHC II	GWLTIGISGPDNGAVAV	ELISPOT	HLA-DR1	NA	Influenza A virus (A/New Caledonia/20/1999(H1N1))
1019632	19386707	neg	MHC II	RTQESECVCVNGSCFTI	ELISPOT	HLA-DR1	NA	Influenza A virus (A/New Caledonia/20/1999(H1N1))
1019632	19386707	neg	MHC II	TDGPSNGAASYKIFKIE	ELISPOT	HLA-DR1	NA	Influenza A virus (A/New Caledonia/20/1999(H1N1))
1019632	19386707	neg	MHC II	IELNAPNFHYEECSCYP	ELISPOT	HLA-DR1	NA	Influenza A virus (A/New Caledonia/20/1999(H1N1))
1019632	19386707	neg	MHC II	NFHYEECSCYPDTGTVM	ELISPOT	HLA-DR1	NA	Influenza A virus (A/New Caledonia/20/1999(H1N1))
1019632	19386707	neg	MHC II	TGTVMCVCRDNWHGSNR	ELISPOT	HLA-DR1	NA	Influenza A virus (A/New Caledonia/20/1999(H1N1))
1019632	19386707	neg	MHC II	HGSNRPWVSFNQNLDYQ	ELISPOT	HLA-DR1	NA	Influenza A virus (A/New Caledonia/20/1999(H1N1))
1019632	19386707	neg	MHC II	RPKDGEGSCNPVTVDGA	ELISPOT	HLA-DR1	NA	Influenza A virus (A/New Caledonia/20/1999(H1N1))
1019632	19386707	neg	MHC II	YKYGNGVWIGRTKSNRL	ELISPOT	HLA-DR1	NA	Influenza A virus (A/New Caledonia/20/1999(H1N1))
1019632	19386707	neg	MHC II	VWIGRTKSNRLRKGFEM	ELISPOT	HLA-DR1	NA	Influenza A virus (A/New Caledonia/20/1999(H1N1))
1019632	19386707	neg	MHC II	VVAITDWSGYSGSFVQH	ELISPOT	HLA-DR1	NA	Influenza A virus (A/New Caledonia/20/1999(H1N1))
1019632	19386707	neg	MHC II	NTTIWTSGSSISFCGVN	ELISPOT	HLA-DR1	NA	Influenza A virus (A/New Caledonia/20/1999(H1N1))
1019632	19386707	neg	MHC II	MDSHTVSSFQVDCFLWH	ELISPOT	HLA-DR1	NS1	Influenza A virus (A/New York/444/2001(H1N1))
1019632	19386707	neg	MHC II	SSFQVDCFLWHVRKQVA	ELISPOT	HLA-DR1	NS1	Influenza A virus (A/New York/444/2001(H1N1))
1019632	19386707	neg	MHC II	CFLWHVRKQVADQDLGD	ELISPOT	HLA-DR1	NS1	Influenza A virus (A/New York/444/2001(H1N1))
1019632	19386707	neg	MHC II	RKQVADQDLGDAPFLDR	ELISPOT	HLA-DR1	NS1	Influenza A virus (A/New York/444/2001(H1N1))
1019632	19386707	neg	MHC II	APFLDRLRRDQKSLKGR	ELISPOT	HLA-DR1	NS1	Influenza A virus (A/New York/444/2001(H1N1))
1019632	19386707	neg	MHC II	LRRDQKSLKGRGSTLGL	ELISPOT	HLA-DR1	NS1	Influenza A virus (A/New York/444/2001(H1N1))
1019632	19386707	neg	MHC II	SLKGRGSTLGLNIETAT	ELISPOT	HLA-DR1	NS1	Influenza A virus (A/New York/444/2001(H1N1))
1019632	19386707	neg	MHC II	STLGLNIETATCVGKQI	ELISPOT	HLA-DR1	NS1	Influenza A virus (A/New York/444/2001(H1N1))
1019632	19386707	neg	MHC II	IETATCVGKQIVERILK	ELISPOT	HLA-DR1	NS1	Influenza A virus (A/New York/444/2001(H1N1))
1019632	19386707	neg	MHC II	LTDMTIEEMSRDWFMLM	ELISPOT	HLA-DR1	NS1	Influenza A virus (A/New York/444/2001(H1N1))
1019632	19386707	neg	MHC II	PLCVRMDQAIMDKNIIL	ELISPOT	HLA-DR1	NS1	Influenza A virus (A/New York/444/2001(H1N1))
1019632	19386707	neg	MHC II	VLIGGLEWNDNTVRVSE	ELISPOT	HLA-DR1	NS1	Influenza A virus (A/New York/444/2001(H1N1))
1019632	19386707	neg	MHC II	EWNDNTVRVSETLQRFA	ELISPOT	HLA-DR1	NS1	Influenza A virus (A/New York/444/2001(H1N1))
1019632	19386707	neg	MHC II	LQRFAWRSSNETGGPPF	ELISPOT	HLA-DR1	NS1	Influenza A virus (A/New York/444/2001(H1N1))
1019632	19386707	neg	MHC II	MASQGTKRSYEQMETDG	ELISPOT	HLA-DR1	NP	Influenza A virus (A/New York/348/2003(H1N1))
1019632	19386707	neg	MHC II	LNDYEGRLIQNSLTIER	ELISPOT	HLA-DR1	NP	Influenza A virus (A/New York/348/2003(H1N1))
1019632	19386707	neg	MHC II	RLIQNSLTIERMVLSAF	ELISPOT	HLA-DR1	NP	Influenza A virus (A/New York/348/2003(H1N1))
1019632	19386707	neg	MHC II	AGKDPKKTGGPIYKRVD	ELISPOT	HLA-DR1	NP	Influenza A virus (A/New York/348/2003(H1N1))
1019632	19386707	neg	MHC II	RQANNGDDATAGLTHIM	ELISPOT	HLA-DR1	NP	Influenza A virus (A/New York/348/2003(H1N1))
1019632	19386707	neg	MHC II	LTHIMIWHSNLNDTTYQ	ELISPOT	HLA-DR1	NP	Influenza A virus (A/New York/348/2003(H1N1))
1019632	19386707	neg	MHC II	DTTYQRTRALVRTGMDP	ELISPOT	HLA-DR1	NP	Influenza A virus (A/New York/348/2003(H1N1))

1019632	19386707	neg	MHC II	AAVKGVGTMVLELIRMI	ELISPOT	HLA-DR1	NP	Influenza A virus (A/New York/348/2003(H1N1))
1019632	19386707	neg	MHC II	LPACVYGPAVASGYDFE	ELISPOT	HLA-DR1	NP	Influenza A virus (A/New York/348/2003(H1N1))
1019632	19386707	neg	MHC II	GYDFEKEGYSLVGVDPF	ELISPOT	HLA-DR1	NP	Influenza A virus (A/New York/348/2003(H1N1))
1019632	19386707	neg	MHC II	AAFEDLRVSSFIRGTRV	ELISPOT	HLA-DR1	NP	Influenza A virus (A/New York/348/2003(H1N1))
1019632	19386707	neg	MHC II	DMSNEGSYFFGDNAEEY	ELISPOT	HLA-DR1	NP	Influenza A virus (A/New York/348/2003(H1N1))
1018856	19446935	pos	MHC II	DQICIGYHANNSTEQVDTIM	MHC tetramer/multimer staining	HLA-DRA*0101/DRB1*0401	HA	Influenza A virus (A/Viet Nam/1203/2004(H5N1))
1018856	19446935	pos	MHC II	DQICIGYHANNSTEQVDTIM	3H-thymidine	HLA-DRA*0101/DRB1*0401	HA	Influenza A virus (A/Viet Nam/1203/2004(H5N1))
1018856	19446935	pos	MHC II	LCDLDGVKPLILRDCSVAGW	MHC tetramer/multimer staining	HLA-DRA*0101/DRB1*0401	HA	Influenza A virus (A/Viet Nam/1203/2004(H5N1))
1018856	19446935	pos	MHC II	LCDLDGVKPLILRDCSVAGW	3H-thymidine	HLA-DRA*0101/DRB1*0401	HA	Influenza A virus (A/Viet Nam/1203/2004(H5N1))
1018856	19446935	pos	MHC II	LCDLDGVKPLILRDCSVAGW	3H-thymidine	HLA-DRA*0101/DRB1*0401	HA	Influenza A virus (A/Viet Nam/1203/2004(H5N1))
1018856	19446935	pos	MHC II	LCDLDGVKPLILRDCSVAGW	3H-thymidine	HLA-DRA*0101/DRB1*0401	HA	Influenza A virus (A/Viet Nam/1203/2004(H5N1))
1018856	19446935	pos	MHC II	LSRINHFEKIQIIPKSSWSS	MHC tetramer/multimer staining	HLA-DRA*0101/DRB1*0401	HA	Influenza A virus (A/Viet Nam/1203/2004(H5N1))
1018856	19446935	pos	MHC II	LSRINHFEKIQIIPKSSWSS	3H-thymidine	HLA-DRA*0101/DRB1*0401	HA	Influenza A virus (A/Viet Nam/1203/2004(H5N1))
1018856	19446935	pos	MHC II	LSRINHFEKIQIIPKSSWSS	3H-thymidine	HLA-DRA*0101/DRB1*0401	HA	Influenza A virus (A/Viet Nam/1203/2004(H5N1))
1018856	19446935	pos	MHC II	KNSTYPTIKRSYNNTNQEDL	MHC tetramer/multimer staining	HLA-DRA*0101/DRB1*0401	HA	Influenza A virus (A/Viet Nam/1203/2004(H5N1))
1018856	19446935	pos	MHC II	GSGYAADKESTQKAIDGVTN	MHC tetramer/multimer staining	HLA-DRA*0101/DRB1*0401	HA	Influenza A virus (A/Viet Nam/1203/2004(H5N1))
1018856	19446935	pos	MHC II	GSGYAADKESTQKAIDGVTN	3H-thymidine	HLA-DRA*0101/DRB1*0401	HA	Influenza A virus (A/Viet Nam/1203/2004(H5N1))
1018856	19446935	pos	MHC II	GSGYAADKESTQKAIDGVTN	3H-thymidine	HLA-DRA*0101/DRB1*0401	HA	Influenza A virus (A/Viet Nam/1203/2004(H5N1))
1018856	19446935	pos	MHC II	NAELLVLMENERTLDFHDSN	MHC tetramer/multimer staining	HLA-DRA*0101/DRB1*0401	HA	Influenza A virus (A/Viet Nam/1203/2004(H5N1))
1018856	19446935	pos	MHC II	NAELLVLMENERTLDFHDSN	3H-thymidine	HLA-DRA*0101/DRB1*0401	HA	Influenza A virus (A/Viet Nam/1203/2004(H5N1))
1018856	19446935	pos	MHC II	NAELLVLMENERTLDFHDSN	3H-thymidine	HLA-DRA*0101/DRB1*0401	HA	Influenza A virus (A/Viet Nam/1203/2004(H5N1))
1018856	19446935	neg	MHC II	MEKIVLLFAIVSLVKSDQIC	MHC tetramer/multimer staining	HLA-DRA*0101/DRB1*0401	HA	Influenza A virus (A/Viet Nam/1203/2004(H5N1))
1018856	19446935	neg	MHC II	AIVSLVKSDQICIGYHANNS	MHC tetramer/multimer staining	HLA-DRA*0101/DRB1*0401	HA	Influenza A virus (A/Viet Nam/1203/2004(H5N1))
1018856	19446935	neg	MHC II	ANNSTEQVDTIMEKNVTVTH	MHC tetramer/multimer staining	HLA-DRA*0101/DRB1*0401	HA	Influenza A virus (A/Viet Nam/1203/2004(H5N1))
1018856	19446935	neg	MHC II	DTIMEKNVTVTHAQDILEKK	MHC tetramer/multimer staining	HLA-DRA*0101/DRB1*0401	HA	Influenza A virus (A/Viet Nam/1203/2004(H5N1))
1018856	19446935	neg	MHC II	TVTHAQDILEKKHNGKLCDL	MHC tetramer/multimer staining	HLA-DRA*0101/DRB1*0401	HA	Influenza A virus (A/Viet Nam/1203/2004(H5N1))
1018856	19446935	neg	MHC II	LEKKHNGKLCDLDGVKPLIL	MHC tetramer/multimer staining	HLA-DRA*0101/DRB1*0401	HA	Influenza A virus (A/Viet Nam/1203/2004(H5N1))
1018856	19446935	neg	MHC II	PLILRDCSVAGWLLGNPMCD	MHC tetramer/multimer staining	HLA-DRA*0101/DRB1*0401	HA	Influenza A virus (A/Viet Nam/1203/2004(H5N1))
1018856	19446935	neg	MHC II	VAGWLLGNPMCDEFINVPEW	MHC tetramer/multimer staining	HLA-DRA*0101/DRB1*0401	HA	Influenza A virus (A/Viet Nam/1203/2004(H5N1))

1018856	19446935	neg	MHC II	PMCDEFINVPEWSYIVEKAN	MHC tetramer/multimer staining	HLA-DRA*0101/DRB1*0401	HA	Influenza A virus (A/Viet Nam/1203/2004(H5N1))
1018856	19446935	neg	MHC II	VPEWSYIVEKANPVNDLCYP	MHC tetramer/multimer staining	HLA-DRA*0101/DRB1*0401	HA	Influenza A virus (A/Viet Nam/1203/2004(H5N1))
1018856	19446935	neg	MHC II	EKANPVNDLCYPGDFNDYEE	MHC tetramer/multimer staining	HLA-DRA*0101/DRB1*0401	HA	Influenza A virus (A/Viet Nam/1203/2004(H5N1))
1018856	19446935	neg	MHC II	LCYPGDFNDYEELKHLLSRI	MHC tetramer/multimer staining	HLA-DRA*0101/DRB1*0401	HA	Influenza A virus (A/Viet Nam/1203/2004(H5N1))
1018856	19446935	neg	MHC II	DYEELKHLLSRINHFEKIQI	MHC tetramer/multimer staining	HLA-DRA*0101/DRB1*0401	HA	Influenza A virus (A/Viet Nam/1203/2004(H5N1))
1018856	19446935	neg	MHC II	KIQIIPKSSWSSHEASLGVS	MHC tetramer/multimer staining	HLA-DRA*0101/DRB1*0401	HA	Influenza A virus (A/Viet Nam/1203/2004(H5N1))
1018856	19446935	neg	MHC II	SWSSHEASLGVSSACPYQGK	MHC tetramer/multimer staining	HLA-DRA*0101/DRB1*0401	HA	Influenza A virus (A/Viet Nam/1203/2004(H5N1))
1018856	19446935	neg	MHC II	LGVSSACPYQGKSSFFRNVV	MHC tetramer/multimer staining	HLA-DRA*0101/DRB1*0401	HA	Influenza A virus (A/Viet Nam/1203/2004(H5N1))
1018856	19446935	neg	MHC II	YQGKSSFFRNVVWLIKKNST	MHC tetramer/multimer staining	HLA-DRA*0101/DRB1*0401	HA	Influenza A virus (A/Viet Nam/1203/2004(H5N1))
1018856	19446935	neg	MHC II	RNVVWLIKKNSTYPTIKRSY	MHC tetramer/multimer staining	HLA-DRA*0101/DRB1*0401	HA	Influenza A virus (A/Viet Nam/1203/2004(H5N1))
1018856	19446935	neg	MHC II	KRSYNNTNQEDLLVLWGIHH	MHC tetramer/multimer staining	HLA-DRA*0101/DRB1*0401	HA	Influenza A virus (A/Viet Nam/1203/2004(H5N1))
1018856	19446935	neg	MHC II	QEDLLVLWGIHHPNDAAEQT	MHC tetramer/multimer staining	HLA-DRA*0101/DRB1*0401	HA	Influenza A virus (A/Viet Nam/1203/2004(H5N1))
1018856	19446935	neg	MHC II	GIHHPNDAAEQTKLYQNPTT	MHC tetramer/multimer staining	HLA-DRA*0101/DRB1*0401	HA	Influenza A virus (A/Viet Nam/1203/2004(H5N1))
1018856	19446935	neg	MHC II	AEQTKLYQNPTTYISVGTST	MHC tetramer/multimer staining	HLA-DRA*0101/DRB1*0401	HA	Influenza A virus (A/Viet Nam/1203/2004(H5N1))
1018856	19446935	neg	MHC II	NPTTYISVGTSTLNQRLVPR	MHC tetramer/multimer staining	HLA-DRA*0101/DRB1*0401	HA	Influenza A virus (A/Viet Nam/1203/2004(H5N1))
1018856	19446935	neg	MHC II	GTSTLNQRLVPRIATRSKVN	MHC tetramer/multimer staining	HLA-DRA*0101/DRB1*0401	HA	Influenza A virus (A/Viet Nam/1203/2004(H5N1))
1018856	19446935	neg	MHC II	LVPRIATRSKVNGQSGRMEF	MHC tetramer/multimer staining	HLA-DRA*0101/DRB1*0401	HA	Influenza A virus (A/Viet Nam/1203/2004(H5N1))
1018856	19446935	neg	MHC II	SKVNGQSGRMEFFWTILKPN	MHC tetramer/multimer staining	HLA-DRA*0101/DRB1*0401	HA	Influenza A virus (A/Viet Nam/1203/2004(H5N1))
1018856	19446935	neg	MHC II	RMEFFWTILKPNDAINFESN	MHC tetramer/multimer staining	HLA-DRA*0101/DRB1*0401	HA	Influenza A virus (A/Viet Nam/1203/2004(H5N1))
1018856	19446935	neg	MHC II	LKPNDAINFESNGNFIAPEY	MHC tetramer/multimer staining	HLA-DRA*0101/DRB1*0401	HA	Influenza A virus (A/Viet Nam/1203/2004(H5N1))
1018856	19446935	neg	MHC II	FESNGNFIAPEYAYKIVKKG	MHC tetramer/multimer staining	HLA-DRA*0101/DRB1*0401	HA	Influenza A virus (A/Viet Nam/1203/2004(H5N1))
1018856	19446935	neg	MHC II	APEYAYKIVKKGDSTIMKSE	MHC tetramer/multimer staining	HLA-DRA*0101/DRB1*0401	HA	Influenza A virus (A/Viet Nam/1203/2004(H5N1))
1018856	19446935	neg	MHC II	VKKGDSTIMKSELEYGNCNT	MHC tetramer/multimer staining	HLA-DRA*0101/DRB1*0401	HA	Influenza A virus (A/Viet Nam/1203/2004(H5N1))
1018856	19446935	neg	MHC II	MKSELEYGNCNTKCQTPMGA	MHC tetramer/multimer staining	HLA-DRA*0101/DRB1*0401	HA	Influenza A virus (A/Viet Nam/1203/2004(H5N1))
1018856	19446935	neg	MHC II	NCNTKCQTPMGAINSSMPFH	MHC tetramer/multimer staining	HLA-DRA*0101/DRB1*0401	HA	Influenza A virus (A/Viet Nam/1203/2004(H5N1))
1018856	19446935	neg	MHC II	PMGAINSSMPFHNIHPLTIG	MHC tetramer/multimer staining	HLA-DRA*0101/DRB1*0401	HA	Influenza A virus (A/Viet Nam/1203/2004(H5N1))
1018856	19446935	neg	MHC II	MPFHNIHPLTIGECPKYVKS	MHC tetramer/multimer staining	HLA-DRA*0101/DRB1*0401	HA	Influenza A virus (A/Viet Nam/1203/2004(H5N1))

1018856	19446935	neg	MHC II	LTIGECPKYVKSNRLVLATG	MHC tetramer/multimer staining	HLA-DRA*0101/DRB1*0401	HA	Influenza A virus (A/Viet Nam/1203/2004(H5N1))
1018856	19446935	neg	MHC II	YVKSNRLVLATGLRNSPQRE	MHC tetramer/multimer staining	HLA-DRA*0101/DRB1*0401	HA	Influenza A virus (A/Viet Nam/1203/2004(H5N1))
1018856	19446935	neg	MHC II	LATGLRNSPQRERRRKKRGL	MHC tetramer/multimer staining	HLA-DRA*0101/DRB1*0401	HA	Influenza A virus (A/Viet Nam/1203/2004(H5N1))
1018856	19446935	neg	MHC II	PQRERRRKKRGLFGAIAGFI	MHC tetramer/multimer staining	HLA-DRA*0101/DRB1*0401	HA	Influenza A virus (A/Viet Nam/1203/2004(H5N1))
1018856	19446935	neg	MHC II	KRGLFGAIAGFIEGGWQGMV	MHC tetramer/multimer staining	HLA-DRA*0101/DRB1*0401	HA	Influenza A virus (A/Viet Nam/1203/2004(H5N1))
1018856	19446935	neg	MHC II	AGFIEGGWQGMVDGWYGYHH	MHC tetramer/multimer staining	HLA-DRA*0101/DRB1*0401	HA	Influenza A virus (A/Viet Nam/1203/2004(H5N1))
1018856	19446935	neg	MHC II	QGMVDGWYGYHHSNEQGSGY	MHC tetramer/multimer staining	HLA-DRA*0101/DRB1*0401	HA	Influenza A virus (A/Viet Nam/1203/2004(H5N1))
1018856	19446935	neg	MHC II	GYHHSNEQGSGYAADKESTQ	MHC tetramer/multimer staining	HLA-DRA*0101/DRB1*0401	HA	Influenza A virus (A/Viet Nam/1203/2004(H5N1))
1018856	19446935	neg	MHC II	ESTQKAIDGVTNKVNSIIDK	MHC tetramer/multimer staining	HLA-DRA*0101/DRB1*0401	HA	Influenza A virus (A/Viet Nam/1203/2004(H5N1))
1018856	19446935	neg	MHC II	GVTNKVNSIIDKMNTQFEAV	MHC tetramer/multimer staining	HLA-DRA*0101/DRB1*0401	HA	Influenza A virus (A/Viet Nam/1203/2004(H5N1))
1018856	19446935	neg	MHC II	IIDKMNTQFEAVGREFNNLE	MHC tetramer/multimer staining	HLA-DRA*0101/DRB1*0401	HA	Influenza A virus (A/Viet Nam/1203/2004(H5N1))
1018856	19446935	neg	MHC II	FEAVGREFNNLERRIENLNK	MHC tetramer/multimer staining	HLA-DRA*0101/DRB1*0401	HA	Influenza A virus (A/Viet Nam/1203/2004(H5N1))
1018856	19446935	neg	MHC II	NNLERRIENLNKKMEDGFLD	MHC tetramer/multimer staining	HLA-DRA*0101/DRB1*0401	HA	Influenza A virus (A/Viet Nam/1203/2004(H5N1))
1018856	19446935	neg	MHC II	NLNKKMEDGFLDVWTYNAEL	MHC tetramer/multimer staining	HLA-DRA*0101/DRB1*0401	HA	Influenza A virus (A/Viet Nam/1203/2004(H5N1))
1018856	19446935	neg	MHC II	GFLDVWTYNAELLVLMENER	MHC tetramer/multimer staining	HLA-DRA*0101/DRB1*0401	HA	Influenza A virus (A/Viet Nam/1203/2004(H5N1))
1018856	19446935	neg	MHC II	NAELLVLLENERTLDFHDSN	MHC tetramer/multimer staining	HLA-DRA*0101/DRB1*0401	HA	H1N1 subtype
1018856	19446935	neg	MHC II	NAELLVLLENERTLDFHDSN	MHC tetramer/multimer staining	HLA-DRA*0101/DRB1*0401	HA	H1N1 subtype
1018856	19446935	neg	MHC II	NAELLVLMENERTLDFHDSN	MHC tetramer/multimer staining	HLA-DRA*0101/DRB1*0401	HA	Influenza A virus (A/Viet Nam/1203/2004(H5N1))
1018856	19446935	neg	MHC II	ENERTLDFHDSNVKNLYDKV	MHC tetramer/multimer staining	HLA-DRA*0101/DRB1*0401	HA	Influenza A virus (A/Viet Nam/1203/2004(H5N1))
1018856	19446935	neg	MHC II	HDSNVKNLYDKVRLQLRDNA	MHC tetramer/multimer staining	HLA-DRA*0101/DRB1*0401	HA	Influenza A virus (A/Viet Nam/1203/2004(H5N1))
1018856	19446935	neg	MHC II	YDKVRLQLRDNAKELGNGCF	MHC tetramer/multimer staining	HLA-DRA*0101/DRB1*0401	HA	Influenza A virus (A/Viet Nam/1203/2004(H5N1))
1018856	19446935	neg	MHC II	RDNAKELGNGCFEFYHKCDN	MHC tetramer/multimer staining	HLA-DRA*0101/DRB1*0401	HA	Influenza A virus (A/Viet Nam/1203/2004(H5N1))
1018856	19446935	neg	MHC II	NGCFEFYHKCDNECMESVRN	MHC tetramer/multimer staining	HLA-DRA*0101/DRB1*0401	HA	Influenza A virus (A/Viet Nam/1203/2004(H5N1))
1018856	19446935	neg	MHC II	KCDNECMESVRNGTYDYPQY	MHC tetramer/multimer staining	HLA-DRA*0101/DRB1*0401	HA	Influenza A virus (A/Viet Nam/1203/2004(H5N1))
1018856	19446935	neg	MHC II	SVRNGTYDYPQYSEEARLKR	MHC tetramer/multimer staining	HLA-DRA*0101/DRB1*0401	HA	Influenza A virus (A/Viet Nam/1203/2004(H5N1))
1018856	19446935	neg	MHC II	YPQYSEEARLKREEISGVKL	MHC tetramer/multimer staining	HLA-DRA*0101/DRB1*0401	HA	Influenza A virus (A/Viet Nam/1203/2004(H5N1))
1018856	19446935	neg	MHC II	RLKREEISGVKLESIGIYQI	MHC tetramer/multimer staining	HLA-DRA*0101/DRB1*0401	HA	Influenza A virus (A/Viet Nam/1203/2004(H5N1))

1018856	19446935	neg	MHC II	GVKLESIGIYQILSIYSTVA	MHC tetramer/multimer staining	HLA-DRA*0101/DRB1*0401	HA	Influenza A virus (A/Viet Nam/1203/2004(H5N1))
1018856	19446935	neg	MHC II	IYQILSIYSTVASSLALAIM	MHC tetramer/multimer staining	HLA-DRA*0101/DRB1*0401	HA	Influenza A virus (A/Viet Nam/1203/2004(H5N1))
1018856	19446935	neg	MHC II	STVASSLALAIMVAGLSLWM	MHC tetramer/multimer staining	HLA-DRA*0101/DRB1*0401	HA	Influenza A virus (A/Viet Nam/1203/2004(H5N1))
1018856	19446935	neg	MHC II	LAIMVAGLSLWMCSNGSLQC	MHC tetramer/multimer staining	HLA-DRA*0101/DRB1*0401	HA	Influenza A virus (A/Viet Nam/1203/2004(H5N1))
1018856	19446935	neg	MHC II	SLWMCSNGSLQCRICI	MHC tetramer/multimer staining	HLA-DRA*0101/DRB1*0401	HA	Influenza A virus (A/Viet Nam/1203/2004(H5N1))
1019592	19524006	pos	MHC I	LTKGILGFVFTLTVPSE	ELISPOT	HLA-A*0201	M1	Influenza A virus (A/New York/348/2003(H1N1))
1019592	19524006	pos	MHC I	ISIAIGIISLMLQIGNI	ELISPOT	HLA-A*0201	NA	Influenza A virus (A/New Caledonia/20/1999(H1N1))
1019592	19524006	pos	MHC I	DQAIMDKNIILKANFSV	ELISPOT	HLA-A*0201	NS1	Influenza A virus (A/New York/444/2001(H1N1))
1019592	19524006	pos	MHC I	PPNFSCIENFRAYVDGF	ELISPOT	HLA-A*0201	PA	Influenza A virus (A/New York/348/2003(H1N1))
1019592	19524006	pos	MHC I	GMMMGMFNMLSTVLGVS	ELISPOT	HLA-A*0201	PB1	Influenza A virus (A/New York/348/2003(H1N1))
1019592	19524006	pos	MHC I	FLARSALILRGSVAHKS	ELISPOT	HLA-A3	NP	Influenza A virus (A/New York/348/2003(H1N1))
1019592	19524006	pos	MHC I	AGALASCMGLIYNRMGA	ELISPOT	HLA-B*3501	M1	Influenza A virus (A/New York/348/2003(H1N1))
1019592	19524006	pos	MHC II	LIAPRGYFKIRSGKSSI	ELISPOT	HLA-DR	HA	Influenza A virus (A/New York/384/2005(H3N2))
1019592	19524006	pos	MHC II	YFKIRSGKSSIMRSDAP	ELISPOT	HLA-DR	HA	Influenza A virus (A/New York/384/2005(H3N2))
1019592	19524006	pos	MHC II	CPRYVKQNTLKLATGMR	ELISPOT	HLA-DR	HA	Influenza A virus (A/New York/384/2005(H3N2))
1019592	19524006	pos	MHC II	NNMDRAVKLYRKLKREI	ELISPOT	HLA-DR	M1	Influenza A virus (A/New York/348/2003(H1N1))
1019592	19524006	pos	MHC II	VKLYRKLKREITFHGAK	ELISPOT	HLA-DR	M1	Influenza A virus (A/New York/348/2003(H1N1))
1019592	19524006	pos	MHC II	RQMVTTTNPLIRHENRM	ELISPOT	HLA-DR	M1	Influenza A virus (A/New York/348/2003(H1N1))
1019592	19524006	pos	MHC II	LIAPRGYFKIRSGKSSI	51 chromium release	HLA-DR1	HA	Influenza A virus (A/New York/384/2005(H3N2))
1019592	19524006	pos	MHC II	SIVPSGPLKAEIAQRLE	ELISPOT	HLA-DRB1*0101	M1	Influenza A virus (A/New York/348/2003(H1N1))
1019592	19524006	pos	MHC II	SIVPSGPLKAEIAQRLE	ELISPOT	HLA-DRB1*1501	M1	Influenza A virus (A/New York/348/2003(H1N1))
1019592	19524006	pos	MHC II	TNPLIRHENRMVLASTT	ELISPOT	HLA-DRB1*1501	M1	Influenza A virus (A/New York/348/2003(H1N1))
1019592	19524006	pos	MHC II	TNPLIRHENRMVLASTT	ELISPOT	HLA-DRB5	M1	Influenza A virus (A/New York/348/2003(H1N1))
1019592	19524006	pos	MHC II	KAMEQMAGSSEQAAEAM	ELISPOT	HLA-DRB5	M1	Influenza A virus (A/New York/348/2003(H1N1))
1019592	19524006	pos	MHC II	GLKNDLLENLQAYQKRM	ELISPOT	HLA-DRB5	M1	Influenza A virus (A/New York/348/2003(H1N1))
1016566	19553306	pos	MHC I	LPFDKSTVM	ELISPOT	HLA-B*0702	NP	H1N1 subtype
1016566	19553306	pos	MHC I	LPFDKTTIM	ELISPOT	HLA-B*0702	NP	H1N1 subtype Influenza A/Oklahoma/7485/01
1016566	19553306	pos	MHC I	LPFDRTTVM	ELISPOT	HLA-B*0702	NP	Influenza A virus (A/Puerto Rico/8/1934(H1N1))
1016566	19553306	pos	MHC I	LPFDRPTIM	ELISPOT	HLA-B*0702	NP	H1N1 subtype
1016566	19553306	pos	MHC I	LPFDKATIM	ELISPOT	HLA-B*0702	NP	H1N1 subtype
1016566	19553306	pos	MHC I	LPFDKSTIM	ELISPOT	HLA-B*0702	NP	H1N1 subtype
1016566	19553306	pos	MHC I	LPFDRTTIM	ELISPOT	HLA-B*0702	NP	H1N1 subtype
1019112	19830726	pos	MHC II	TNPLIRHENRMVLASTTAKA	MHC tetramer/multimer staining	HLA-DRB1*0301	M1	Influenza A virus (A/Puerto Rico/8/1934(H1N1))
1019112	19830726	pos	MHC II	VLMEWLKTRPILSPLTKGIL	MHC tetramer/multimer staining	HLA-DRB1*1101	M1	Influenza A virus (A/Puerto Rico/8/1934(H1N1))
1019112	19830726	pos	MHC II	VKLYRKLKREITFHGAKEIS	MHC tetramer/multimer staining	HLA-DRB1*1101	M1	Influenza A virus (A/Puerto Rico/8/1934(H1N1))
1019112	19830726	pos	MHC II	EAMEVASQARQMVQAMRTIG	MHC tetramer/multimer	HLA-DRB1*1101	M1	Influenza A virus (A/Puerto Rico/8/1934(H1N1))

					staining			
1019205	20060099	pos	MHC I	GILGFVFTL	intracellular cytokine staining (ICS)	HLA-A*0201	M1	Influenza A virus (A/Puerto Rico/8/1934(H1N1))
1019205	20060099	pos	MHC I	GILGFVFTL	in vivo assay	HLA-A*0201	M1	Influenza A virus (A/Puerto Rico/8/1934(H1N1))
1019205	20060099	pos	MHC I	GILGFVFTL	challenge assay	HLA-A*0201	M1	Influenza A virus (A/Puerto Rico/8/1934(H1N1))
1019205	20060099	pos	MHC I	GILGFVFTL	challenge assay	HLA-A*0201	M1	Influenza A virus (A/Puerto Rico/8/1934(H1N1))
1019526	20071564	pos	MHC II	QRALYHTENAYVSVVS	3H-thymidine	HLA-DRB1*0401	HA	Influenza A virus (A/New Caledonia/20/1999(H1N1))
1019526	20071564	pos	MHC II	IIFEANGNLIAPWYAFA	3H-thymidine	HLA-DRB1*0401	HA	Influenza A virus (A/New Caledonia/20/1999(H1N1))
1019526	20071564	pos	MHC II	TGLRNIPSIQSRGLFGAIA	3H-thymidine	HLA-DRB1*0401	HA	Influenza A virus (A/New Caledonia/20/1999(H1N1))
1019526	20071564	pos	MHC II	SVIEKMNTQFTAVGKE	3H-thymidine	HLA-DRB1*0401	HA	Influenza A virus (A/New Caledonia/20/1999(H1N1))
1019526	20071564	pos	MHC II	ELLVLLENERTLDFHDS	3H-thymidine	HLA-DRB1*0401	HA	Influenza A virus (A/New Caledonia/20/1999(H1N1))
1019526	20071564	pos	MHC II	ELLVLLENERTLDFHDS	3H-thymidine	HLA-DRB1*0401	HA	Influenza A virus (A/New Caledonia/20/1999(H1N1))
1019526	20071564	pos	MHC II	ELLVLLENERTLDFHDS	ELISPOT	HLA-DRB1*0401	HA	Influenza A virus (A/New Caledonia/20/1999(H1N1))
1019526	20071564	pos	MHC II	ELLVLLENERTLDYHDS	3H-thymidine	HLA-DRB1*0401	HA	Influenza A virus (A/California/04/2009(H1N1))
1019526	20071564	pos	MHC II	ELLVLLENERTLDYHDS	ELISPOT	HLA-DRB1*0401	HA	Influenza A virus (A/California/04/2009(H1N1))
1019526	20071564	pos	MHC II	TYVLSIIPSGPLKAEIAQRL	3H-thymidine	HLA-DRB1*0401	M1	Influenza A virus (A/California/04/2009(H1N1))
1019526	20071564	pos	MHC II	TYVLSIIPSGPLKAEIAQRL	ELISPOT	HLA-DRB1*0401	M1	Influenza A virus (A/California/04/2009(H1N1))
1019526	20071564	pos	MHC II	TYVLSIVPSGPLKAEIAQRL	3H-thymidine	HLA-DRB1*0401	M1	Influenza A virus (A/New Caledonia/20/1999(H1N1))
1019526	20071564	pos	MHC II	TYVLSIVPSGPLKAEIAQRL	3H-thymidine	HLA-DRB1*0401	M1	Influenza A virus (A/New Caledonia/20/1999(H1N1))
1019526	20071564	pos	MHC II	TYVLSIVPSGPLKAEIAQRL	ELISPOT	HLA-DRB1*0401	M1	Influenza A virus (A/New Caledonia/20/1999(H1N1))
1019526	20071564	pos	MHC II	ILGFVFTLTVPSERG	3H-thymidine	HLA-DRB1*0401	M1	Influenza A virus (A/New Caledonia/20/1999(H1N1))
1019526	20071564	pos	MHC II	GWAIYSKDNSVRIGSKG	3H-thymidine	HLA-DRB1*0401	NA	Influenza A virus (A/California/04/2009(H1N1))
1019526	20071564	pos	MHC II	GWAIYSKDNSVRIGSKG	ELISPOT	HLA-DRB1*0401	NA	Influenza A virus (A/California/04/2009(H1N1))
1019526	20071564	pos	MHC II	GWAIYTKDNSIRIGSKG	3H-thymidine	HLA-DRB1*0401	NA	Influenza A virus (A/New Caledonia/20/1999(H1N1))
1019526	20071564	pos	MHC II	GWAIYTKDNSIRIGSKG	3H-thymidine	HLA-DRB1*0401	NA	Influenza A virus (A/New Caledonia/20/1999(H1N1))
1019526	20071564	pos	MHC II	GWAIYTKDNSIRIGSKG	ELISPOT	HLA-DRB1*0401	NA	Influenza A virus (A/New Caledonia/20/1999(H1N1))
1019526	20071564	pos	MHC II	GQASYKIFRIEKGKIVK	3H-thymidine	HLA-DRB1*0401	NA	Influenza A virus (A/California/04/2009(H1N1))
1019526	20071564	pos	MHC II	GQASYKIFRIEKGKIVK	ELISPOT	HLA-DRB1*0401	NA	Influenza A virus (A/California/04/2009(H1N1))
1019526	20071564	pos	MHC II	GAASYKIFKIEKGKVTK	3H-thymidine	HLA-DRB1*0401	NA	Influenza A virus (A/New Caledonia/20/1999(H1N1))
1019526	20071564	pos	MHC II	GAASYKIFKIEKGKVTK	3H-thymidine	HLA-DRB1*0401	NA	Influenza A virus (A/New Caledonia/20/1999(H1N1))
1019526	20071564	pos	MHC II	GAASYKIFKIEKGKVTK	ELISPOT	HLA-DRB1*0401	NA	Influenza A virus (A/New Caledonia/20/1999(H1N1))
1019526	20071564	pos	MHC II	GFEMIWDPNGWTGTDN	3H-thymidine	HLA-DRB1*0401	NA	Influenza A virus (A/California/04/2009(H1N1))
1019526	20071564	pos	MHC II	GFEMIWDPNGWTGTDN	ELISPOT	HLA-DRB1*0401	NA	Influenza A virus (A/California/04/2009(H1N1))
1019526	20071564	pos	MHC II	GFEMIWDPNGWTDTDS	3H-thymidine	HLA-DRB1*0401	NA	Influenza A virus (A/New Caledonia/20/1999(H1N1))
1019526	20071564	pos	MHC II	GFEMIWDPNGWTDTDS	3H-thymidine	HLA-DRB1*0401	NA	Influenza A virus (A/New Caledonia/20/1999(H1N1))
1019526	20071564	pos	MHC II	GFEMIWDPNGWTDTDS	ELISPOT	HLA-DRB1*0401	NA	Influenza A virus (A/New Caledonia/20/1999(H1N1))
1019526	20071564	pos	MHC II	NPAHKSQLVWMACNSAAFED	3H-thymidine	HLA-DRB1*0401	NP	Influenza A virus (A/New Caledonia/20/1999(H1N1))
1019526	20071564	pos	MHC II	NPAHKSQLVWMACNSAAFED	3H-thymidine	HLA-DRB1*0401	NP	Influenza A virus (A/New Caledonia/20/1999(H1N1))
1019526	20071564	pos	MHC II	NPAHKSQLVWMACNSAAFED	ELISPOT	HLA-DRB1*0401	NP	Influenza A virus (A/New Caledonia/20/1999(H1N1))
1019526	20071564	pos	MHC II	RTEVIRMMESAKPEDLSFQ	3H-thymidine	HLA-DRB1*0401	NP	Influenza A virus (A/California/04/2009(H1N1))

1019526	20071564	pos	MHC II	RTEVIRMMESAKPEDLSFQ	ELISPOT	HLA-DRB1*0401	NP	Influenza A virus (A/California/04/2009(H1N1))
1019526	20071564	pos	MHC II	RAEIIKMMESARPEEVSFQ	3H-thymidine	HLA-DRB1*0401	NP	Influenza A virus (A/New Caledonia/20/1999(H1N1))
1019526	20071564	pos	MHC II	RAEIIKMMESARPEEVSFQ	3H-thymidine	HLA-DRB1*0401	NP	Influenza A virus (A/New Caledonia/20/1999(H1N1))
1019526	20071564	pos	MHC II	RAEIIKMMESARPEEVSFQ	ELISPOT	HLA-DRB1*0401	NP	Influenza A virus (A/New Caledonia/20/1999(H1N1))
1019526	20071564	pos	MHC II	NPAHKSQLVWMACHSAAFEI	3H-thymidine	HLA-DRB1*0401	NP	Influenza A virus (A/California/04/2009(H1N1))
1019526	20071564	pos	MHC II	NPAHKSQLVWMACHSAAFEI	ELISPOT	HLA-DRB1*0401	NP	Influenza A virus (A/California/04/2009(H1N1))
1019526	20071564	pos	MHC II	TGTGYTMDTVNRTHQ	3H-thymidine	HLA-DRB1*0401	PB1	Influenza A virus (A/New Caledonia/20/1999(H1N1))
1019526	20071564	pos	MHC II	KLANVVRKMMTNSQDTE	3H-thymidine	HLA-DRB1*0401	PB1	Influenza A virus (A/New Caledonia/20/1999(H1N1))
1019526	20071564	pos	MHC II	GMFNMLSTVLGVSILNLGQ	3H-thymidine	HLA-DRB1*0401	PB1	Influenza A virus (A/New Caledonia/20/1999(H1N1))
1020782		pos	MHC II	MNPNQKIITIGSVCMTI	MHC tetramer/multimer staining	HLA-DRB1*0101	NA	Influenza A virus (A/California/04/2009(H1N1))
1020782		pos	MHC II	LECRTFFLTQGALLNDK	MHC tetramer/multimer staining	HLA-DRB1*0101	NA	Influenza A virus (A/California/04/2009(H1N1))
1020782		pos	MHC II	YKIFRIEKGKIVKSVEM	MHC tetramer/multimer staining	HLA-DRB1*0101	NA	Influenza A virus (A/California/04/2009(H1N1))
1020782		pos	MHC II	EKGKIVKSVEMNAPNYH	MHC tetramer/multimer staining	HLA-DRB1*0101	NA	Influenza A virus (A/California/04/2009(H1N1))
1020782		pos	MHC II	NQNLEYQIGYICSGIFG	MHC tetramer/multimer staining	HLA-DRB1*0101	NA	Influenza A virus (A/California/04/2009(H1N1))
1019566		pos	MHC II	TLAGNSSLCSISGWAIY	MHC tetramer/multimer staining	HLA-DRB1*0101	NA	Influenza A virus (A/New Caledonia/20/1999(H1N1))
1019566		pos	MHC II	CSHLECRTFFLTQGALL	MHC tetramer/multimer staining	HLA-DRB1*0101	NA	Influenza A virus (A/New Caledonia/20/1999(H1N1))
1019566		pos	MHC II	RTFFLTQGALLNDKHSN	MHC tetramer/multimer staining	HLA-DRB1*0101	NA	Influenza A virus (A/New Caledonia/20/1999(H1N1))
1019566		pos	MHC II	GAASYKIFKIEKGKVTK	MHC tetramer/multimer staining	HLA-DRB1*0101	NA	Influenza A virus (A/New Caledonia/20/1999(H1N1))
1019566		pos	MHC II	IFKIEKGKVTKSIELNA	MHC tetramer/multimer staining	HLA-DRB1*0101	NA	Influenza A virus (A/New Caledonia/20/1999(H1N1))
1019566		pos	MHC II	KGFEMIWDPNGWTDTDS	MHC tetramer/multimer staining	HLA-DRB1*0101	NA	Influenza A virus (A/New Caledonia/20/1999(H1N1))
1019566		pos	MHC II	DCIRPCFWVELVRGLPR	MHC tetramer/multimer staining	HLA-DRB1*0101	NA	Influenza A virus (A/New Caledonia/20/1999(H1N1))
1019566		pos	MHC II	YQFALGQGTTLNNVHSN	MHC tetramer/multimer staining	HLA-DRB1*0101	NA	Influenza A virus (A/New York/384/2005(H3N2))
1019566		pos	MHC II	WSKEILRTQESECVCIN	MHC tetramer/multimer staining	HLA-DRB1*0101	NA	Influenza A virus (A/New York/384/2005(H3N2))
1019566		pos	MHC II	CVCINGTCTVVMTDGSA	MHC tetramer/multimer staining	HLA-DRB1*0101	NA	Influenza A virus (A/New York/384/2005(H3N2))
1019566		pos	MHC II	GKADTKILFIEEGKIVH	MHC tetramer/multimer staining	HLA-DRB1*0101	NA	Influenza A virus (A/New York/384/2005(H3N2))
1019566		pos	MHC II	NRSGYSGIFSVEGKSCI	MHC tetramer/multimer staining	HLA-DRB1*0101	NA	Influenza A virus (A/New York/384/2005(H3N2))
1019566		pos	MHC II	GWAIYTKDNSIRIGSKG	MHC tetramer/multimer staining	HLA-DRB1*0301	NA	Influenza A virus (A/New Caledonia/20/1999(H1N1))
1019566		pos	MHC II	FCGVNSDTANWSWPDGA	MHC tetramer/multimer staining	HLA-DRB1*0301	NA	Influenza A virus (A/New Caledonia/20/1999(H1N1))
1019566		pos	MHC II	GFAPFSKDNSIRLSAGG	MHC tetramer/multimer staining	HLA-DRB1*0301	NA	Influenza A virus (A/New York/384/2005(H3N2))
1019566		pos	MHC II	TDGSASGKADTKILFIE	MHC tetramer/multimer staining	HLA-DRB1*0301	NA	Influenza A virus (A/New York/384/2005(H3N2))

1016040	pos	MHC II	GTMVMELVRMIKRGI	MHC tetramer/multimer staining	HLA-DRB1*0301	NP	Influenza A virus (A/Puerto Rico/8/1934(H1N1))
1020782	pos	MHC II	WAIYSKDNSVRIGSKGD	MHC tetramer/multimer staining	HLA-DRB1*0401	NA	Influenza A virus (A/California/04/2009(H1N1))
1020782	pos	MHC II	IITDTIKSWRNNILRTQ	MHC tetramer/multimer staining	HLA-DRB1*0401	NA	Influenza A virus (A/California/04/2009(H1N1))
1020782	pos	MHC II	KSWRNNILRTQESECAC	MHC tetramer/multimer staining	HLA-DRB1*0401	NA	Influenza A virus (A/California/04/2009(H1N1))
1020782	pos	MHC II	ILRTQESECACVNGSCF	MHC tetramer/multimer staining	HLA-DRB1*0401	NA	Influenza A virus (A/California/04/2009(H1N1))
1020782	pos	MHC II	SNGQASYKIFRIEKGKI	MHC tetramer/multimer staining	HLA-DRB1*0401	NA	Influenza A virus (A/California/04/2009(H1N1))
1020782	pos	MHC II	SRNGFEMIWDPNGWTGT	MHC tetramer/multimer staining	HLA-DRB1*0401	NA	Influenza A virus (A/California/04/2009(H1N1))
1019566	pos	MHC II	GWAIYTKDNSIRIGSKG	MHC tetramer/multimer staining	HLA-DRB1*0401	NA	Influenza A virus (A/New Caledonia/20/1999(H1N1))
1019566	pos	MHC II	GAVAVLKYNGIITETIK	MHC tetramer/multimer staining	HLA-DRB1*0401	NA	Influenza A virus (A/New Caledonia/20/1999(H1N1))
1019566	pos	MHC II	GAASYKIFKIEKGKVTK	MHC tetramer/multimer staining	HLA-DRB1*0401	NA	Influenza A virus (A/New Caledonia/20/1999(H1N1))
1019566	pos	MHC II	KSNRLRKGFEMIWDPNG	MHC tetramer/multimer staining	HLA-DRB1*0401	NA	Influenza A virus (A/New Caledonia/20/1999(H1N1))
1019566	pos	MHC II	KGFEMIWDPNGWTDTDS	MHC tetramer/multimer staining	HLA-DRB1*0401	NA	Influenza A virus (A/New Caledonia/20/1999(H1N1))
1019566	pos	MHC II	GFAPFSKDNSIRLSAGG	MHC tetramer/multimer staining	HLA-DRB1*0401	NA	Influenza A virus (A/New York/384/2005(H3N2))
1019566	pos	MHC II	TCTVVMTDGSASGKADT	MHC tetramer/multimer staining	HLA-DRB1*0401	NA	Influenza A virus (A/New York/384/2005(H3N2))
1019566	pos	MHC II	GKIVHTSTLSGSAQHVE	MHC tetramer/multimer staining	HLA-DRB1*0401	NA	Influenza A virus (A/New York/384/2005(H3N2))
1019566	pos	MHC II	NRSGYSGIFSVEGKSCI	MHC tetramer/multimer staining	HLA-DRB1*0401	NA	Influenza A virus (A/New York/384/2005(H3N2))
1020782	pos	MHC II	MNPNQKIITIGSVCMTI	MHC tetramer/multimer staining	HLA-DRB1*0404	NA	Influenza A virus (A/California/04/2009(H1N1))
1020782	pos	MHC II	IISLMLQIGNIISIWAS	MHC tetramer/multimer staining	HLA-DRB1*0404	NA	Influenza A virus (A/New Caledonia/20/1999(H1N1))
1020782	pos	MHC II	LQIGNIISIWASHSIQT	MHC tetramer/multimer staining	HLA-DRB1*0404	NA	Influenza A virus (A/New Caledonia/20/1999(H1N1))
1020782	pos	MHC II	KSNRLRKGFEMIWDPNG	MHC tetramer/multimer staining	HLA-DRB1*0404	NA	Influenza A virus (A/New Caledonia/20/1999(H1N1))
1020782	pos	MHC II	KGFEMIWDPNGWTDTDS	MHC tetramer/multimer staining	HLA-DRB1*0404	NA	Influenza A virus (A/New Caledonia/20/1999(H1N1))
1020782	pos	MHC II	FSVKQDVVAITDWSGYS	MHC tetramer/multimer staining	HLA-DRB1*0404	NA	Influenza A virus (A/New Caledonia/20/1999(H1N1))
1020782	pos	MHC II	VVAITDWSGYSGSFVQH	MHC tetramer/multimer staining	HLA-DRB1*0404	NA	Influenza A virus (A/New Caledonia/20/1999(H1N1))
1020782	pos	MHC II	NTTIWTSGSSISFCGVN	MHC tetramer/multimer staining	HLA-DRB1*0404	NA	Influenza A virus (A/New Caledonia/20/1999(H1N1))
1019566	pos	MHC II	MNPNQKIITIGSVSLTI	MHC tetramer/multimer staining	HLA-DRB1*0404	NA	Influenza A virus (A/New York/384/2005(H3N2))
1019566	pos	MHC II	GQGTTLNNVHSNDTVHD	MHC tetramer/multimer staining	HLA-DRB1*0404	NA	Influenza A virus (A/New York/384/2005(H3N2))
1019566	pos	MHC II	ILFIEEGKIVHTSTLSG	MHC tetramer/multimer staining	HLA-DRB1*0404	NA	Influenza A virus (A/New York/384/2005(H3N2))

1019566	pos	MHC II	GKIVHTSTLSGSAQHVE	MHC tetramer/multimer staining	HLA-DRB1*0404	NA	Influenza A virus (A/New York/384/2005(H3N2))
1020782	pos	MHC II	QIGNIISIWISHSIQLG	MHC tetramer/multimer staining	HLA-DRB1*0701	NA	Influenza A virus (A/California/04/2009(H1N1))
1020782	pos	MHC II	FSFKYGNGVWIGRTKSI	MHC tetramer/multimer staining	HLA-DRB1*0701	NA	Influenza A virus (A/California/04/2009(H1N1))
1020782	pos	MHC II	NGVWIGRTKSISSRNGF	MHC tetramer/multimer staining	HLA-DRB1*0701	NA	Influenza A virus (A/California/04/2009(H1N1))
1020782	pos	MHC II	LECRTFFLTQGALLNDK	MHC tetramer/multimer staining	HLA-DRB1*0701	NA	Influenza A virus (A/California/04/2009(H1N1))
1020782	pos	MHC II	MNPNQKIITIGSVCMTI	MHC tetramer/multimer staining	HLA-DRB1*0701	NA	Influenza A virus (A/California/04/2009(H1N1))
1020782	pos	MHC II	MNPNQKIITIGSVCMTI	MHC tetramer/multimer staining	HLA-DRB1*0701	NA	Influenza A virus (A/California/04/2009(H1N1))
1020782	pos	MHC II	WAIYSKDNSVRIGSKGD	MHC tetramer/multimer staining	HLA-DRB1*0701	NA	Influenza A virus (A/California/04/2009(H1N1))
1020782	pos	MHC II	GWAIYTKDNSIRIGSKG	MHC tetramer/multimer staining	HLA-DRB1*0701	NA	Influenza A virus (A/New Caledonia/20/1999(H1N1))
1020782	pos	MHC II	CSHLECRTFFLTQGALL	MHC tetramer/multimer staining	HLA-DRB1*0701	NA	Influenza A virus (A/New Caledonia/20/1999(H1N1))
1020782	pos	MHC II	RTFFLTQGALLNDKHSN	MHC tetramer/multimer staining	HLA-DRB1*0701	NA	Influenza A virus (A/New Caledonia/20/1999(H1N1))
1020782	pos	MHC II	GAVAVLKYNGIITETIK	MHC tetramer/multimer staining	HLA-DRB1*0701	NA	Influenza A virus (A/New Caledonia/20/1999(H1N1))
1020782	pos	MHC II	IFKIEKGKVTKSIELNA	MHC tetramer/multimer staining	HLA-DRB1*0701	NA	Influenza A virus (A/New Caledonia/20/1999(H1N1))
1020782	pos	MHC II	YKYGNGVWIGRTKSNRL	MHC tetramer/multimer staining	HLA-DRB1*0701	NA	Influenza A virus (A/New Caledonia/20/1999(H1N1))
1020782	pos	MHC II	VWIGRTKSNRLRKGFEM	MHC tetramer/multimer staining	HLA-DRB1*0701	NA	Influenza A virus (A/New Caledonia/20/1999(H1N1))
1019566	pos	MHC II	DDGNDVWMGRTISEKLR	MHC tetramer/multimer staining	HLA-DRB1*0701	NA	Influenza A virus (A/New York/384/2005(H3N2))
1019566	pos	MHC II	WMGRTISEKLRSGYETF	MHC tetramer/multimer staining	HLA-DRB1*0701	NA	Influenza A virus (A/New York/384/2005(H3N2))
1019566	pos	MHC II	EETEVLWTSNSIVVFCG	MHC tetramer/multimer staining	HLA-DRB1*0701	NA	Influenza A virus (A/New York/384/2005(H3N2))
1020782	pos	MHC II	GSKGDVFVIREPFISCS	MHC tetramer/multimer staining	HLA-DRB1*1101	NA	Influenza A virus (A/California/04/2009(H1N1))
1020782	pos	MHC II	FVIREPFISCSPLECRT	MHC tetramer/multimer staining	HLA-DRB1*1101	NA	Influenza A virus (A/California/04/2009(H1N1))
1020782	pos	MHC II	NGVWIGRTKSISSRNGF	MHC tetramer/multimer staining	HLA-DRB1*1101	NA	Influenza A virus (A/California/04/2009(H1N1))
1020782	pos	MHC II	SNGQASYKIFRIEKGKI	MHC tetramer/multimer staining	HLA-DRB1*1101	NA	Influenza A virus (A/California/04/2009(H1N1))
1020782	pos	MHC II	YKIFRIEKGKIVKSVEM	MHC tetramer/multimer staining	HLA-DRB1*1101	NA	Influenza A virus (A/California/04/2009(H1N1))
1020782	pos	MHC II	DVFVIREPFISCSHLE	MHC tetramer/multimer staining	HLA-DRB1*1101	NA	Influenza A virus (A/New Caledonia/20/1999(H1N1))
1020782	pos	MHC II	GAASYKIFKIEKGKVTK	MHC tetramer/multimer staining	HLA-DRB1*1101	NA	Influenza A virus (A/New Caledonia/20/1999(H1N1))
1020782	pos	MHC II	DCIRPCFWVELVRGLPR	MHC tetramer/multimer staining	HLA-DRB1*1101	NA	Influenza A virus (A/New Caledonia/20/1999(H1N1))
1020782	pos	MHC II	FWVELVRGLPRENTTIW	MHC tetramer/multimer staining	HLA-DRB1*1101	NA	Influenza A virus (A/New Caledonia/20/1999(H1N1))

1020782	pos	MHC II	RTFFLTQGALLNDKHSN	MHC tetramer/multimer staining	HLA-DRB1*1101	NA	Influenza A virus (A/New Caledonia/20/1999(H1N1))
1019566	pos	MHC II	GKADTKILFIEEGKIVH	MHC tetramer/multimer staining	HLA-DRB1*1101	NA	Influenza A virus (A/New York/384/2005(H3N2))
1019566	pos	MHC II	ILFIEEGKIVHTSTLSG	MHC tetramer/multimer staining	HLA-DRB1*1101	NA	Influenza A virus (A/New York/384/2005(H3N2))
1019566	pos	MHC II	SEKLRSGYETFKVIEGW	MHC tetramer/multimer staining	HLA-DRB1*1101	NA	Influenza A virus (A/New York/384/2005(H3N2))
1019566	pos	MHC II	RCFYVELIRGRKEETEV	MHC tetramer/multimer staining	HLA-DRB1*1101	NA	Influenza A virus (A/New York/384/2005(H3N2))
1016040	pos	MHC II	RALYHTENAYVSVVS	MHC tetramer/multimer staining	HLA-DRB1*1501	HA	Influenza A virus (A/New Caledonia/20/1999(H1N1))
1016040	pos	MHC II	GNLIAPWYAFALSRG	MHC tetramer/multimer staining	HLA-DRB1*1501	HA	Influenza A virus (A/New Caledonia/20/1999(H1N1))
1016040	pos	MHC II	DGFLDIWTYNAELLV	MHC tetramer/multimer staining	HLA-DRB1*1501	HA	Influenza A virus (A/New Caledonia/20/1999(H1N1))
1016040	pos	MHC II	SRLNWLTHLKYKYPA	MHC tetramer/multimer staining	HLA-DRB1*1501	HA	Influenza A virus (A/Wyoming/03/2003(H3N2))
1020782	pos	MHC II	DNGAVAVLKYNGIITDT	MHC tetramer/multimer staining	HLA-DRB1*1501	NA	Influenza A virus (A/California/04/2009(H1N1))
1020782	pos	MHC II	VLKYNGIITDTIKSWRN	MHC tetramer/multimer staining	HLA-DRB1*1501	NA	Influenza A virus (A/California/04/2009(H1N1))
1020782	pos	MHC II	IITDTIKSWRNNILRTQ	MHC tetramer/multimer staining	HLA-DRB1*1501	NA	Influenza A virus (A/California/04/2009(H1N1))
1020782	pos	MHC II	KSWRNNILRTQESECAC	MHC tetramer/multimer staining	HLA-DRB1*1501	NA	Influenza A virus (A/California/04/2009(H1N1))
1020782	pos	MHC II	MNPNQKIITIGSVCMTI	MHC tetramer/multimer staining	HLA-DRB1*1501	NA	Influenza A virus (A/California/04/2009(H1N1))
1020782	pos	MHC II	QIGNIISIWISHSIQLG	MHC tetramer/multimer staining	HLA-DRB1*1501	NA	Influenza A virus (A/California/04/2009(H1N1))
1020782	pos	MHC II	VCNQRIITYENSTWVNH	MHC tetramer/multimer staining	HLA-DRB1*1501	NA	Influenza A virus (A/New Caledonia/20/1999(H1N1))
1020782	pos	MHC II	CVCVNGSCFTIMTDGPS	MHC tetramer/multimer staining	HLA-DRB1*1501	NA	Influenza A virus (A/New Caledonia/20/1999(H1N1))
1020782	pos	MHC II	GAVAVLKYNGIITETIK	MHC tetramer/multimer staining	HLA-DRB1*1501	NA	Influenza A virus (A/New Caledonia/20/1999(H1N1))
1020782	pos	MHC II	LQIGNIISIWASHSIQT	MHC tetramer/multimer staining	HLA-DRB1*1501	NA	Influenza A virus (A/New Caledonia/20/1999(H1N1))
1020782	pos	MHC II	LDCIRPCFWVELIRGRP	MHC tetramer/multimer staining	HLA-DRB5*0101	NA	Influenza A virus (A/California/04/2009(H1N1))
1020782	pos	MHC II	CFWVELIRGRPKENTIW	MHC tetramer/multimer staining	HLA-DRB5*0101	NA	Influenza A virus (A/California/04/2009(H1N1))
1020782	pos	MHC II	EKGKIVKSVEMNAPNYH	MHC tetramer/multimer staining	HLA-DRB5*0101	NA	Influenza A virus (A/California/04/2009(H1N1))
1020782	pos	MHC II	GSKGDVFVIREPFISCS	MHC tetramer/multimer staining	HLA-DRB5*0101	NA	Influenza A virus (A/California/04/2009(H1N1))
1020782	pos	MHC II	LECRTFFLTQGALLNDK	MHC tetramer/multimer staining	HLA-DRB5*0101	NA	Influenza A virus (A/California/04/2009(H1N1))
1020782	pos	MHC II	YKIFRIEKGKIVKSVEM	MHC tetramer/multimer staining	HLA-DRB5*0101	NA	Influenza A virus (A/California/04/2009(H1N1))
1019566	pos	MHC II	RCFYVELIRGRKEETEV	MHC tetramer/multimer staining	HLA-DRB5*0101	NA	Influenza A virus (A/New York/384/2005(H3N2))
