# Supplementary material for: Patterns of Predicted T-Cell Epitopes Associated with Antigenic Drift in Influenza H3N2 Hemagglutinin
Source: PLoS One. 2011 Oct 24;6(10):e26711. doi: 10.1371/journal.pone.0026711 (PMC3200361; doi:10.1371/journal.pone.0026711)
Supplement: Table S2 — Example of data set showing change in predicted MHC binding affinity with cluster transition changes in amino acids. (PDF) [file pone.0026711.s009.pdf]

Table S2: Example of data set showing change in predicted MHC binding affinity with cluster transition changes in amino acids.

MHC I (Highly mutable region) HK68 -> EN72 Transition

Classification	Change	Yr Old Cluster	Yr New Cluster	pos	Allele	Old Peptide	New Peptide	Delta Peptide	Delta Pattern	Old Sigma	New Sigma	N
Lost	Affinity Loss	1968_HK68	1972_EN72	166	nB_0702	RLNWLTKSG	RLNWLYKSE	RLNWL*KS*	'000001001'	-1.07	1.72	1
					nB_0801	RLNWLTKSG	RLNWLYKSE	RLNWL*KS*	'000001001'	-1.23	1.2	1
				168	nA_0202	NWLTKSGST	NWLYKSECT	NWL*KS**T	'000100110'	-1.48	0.3	1
				169	nA_2402	WLTKSGSTY	WLYKSECTY	WL*KS**TY	'001001100'	-3	0.86	1
					nB_1501	WLTKSGSTY	WLYKSECTY	WL*KS**TY	'001001100'	-2.04	0.53	1
					nB_3501	WLTKSGSTY	WLYKSECTY	WL*KS**TY	'001001100'	-1.85	0.86	1
					nB_4001	WLTKSGSTY	WLYKSECTY	WL*KS**TY	'001001100'	-1.01	0.97	1
					nB_4002	WLTKSGSTY	WLYKSECTY	WL*KS**TY	'001001100'	-1.27	0.34	1
					nB_5101	WLTKSGSTY	WLYKSECTY	WL*KS**TY	'001001100'	-1.04	0.24	1
				171	nA_2601	TKSGSTYPV	YKSECTYPV	*KS**TYPV	'100110000'	-2.63	1.02	1
					nB_5101	TKSGSTYPV	YKSECTYPV	*KS**TYPV	'100110000'	-1.45	0.54	1
				172	nB_4002	KSGSTYPVL	KSECTYPVQ	KS**TYPV*	'001100001'	-1.75	0.52	1
					nB_4402	KSGSTYPVL	KSECTYPVQ	KS**TYPV*	'001100001'	-1.18	1.2	1
				174	nB_3501	GSTYPVLNV	ECTYPVQNV	**TYPV*NV	'110000100'	-1.08	0.24	1
New	Affinity Increase	1968_HK68	1972_EN72	166	nA_1101	RLNWLTKSG	RLNWLYKSE	RLNWL*KS*	'000001001'	1.48	-1.37	1
					nA_2902	RLNWLTKSG	RLNWLYKSE	RLNWL*KS*	'000001001'	-0.89	-2.6	1
					nA_3001	RLNWLTKSG	RLNWLYKSE	RLNWL*KS*	'000001001'	0.53	-1.65	1
					nA_6801	RLNWLTKSG	RLNWLYKSE	RLNWL*KS*	'000001001'	-0.27	-1.64	1
					nB_5301	RLNWLTKSG	RLNWLYKSE	RLNWL*KS*	'000001001'	0.31	-1.51	1
				167	nA_0201	LNWLTKSGS	LNWLYKSEC	LNWL*KS**	'000010011'	-0.67	-1.92	1
					nA_0206	LNWLTKSGS	LNWLYKSEC	LNWL*KS**	'000010011'	-0.44	-1.66	1
					nA_0301	LNWLTKSGS	LNWLYKSEC	LNWL*KS**	'000010011'	-0.33	-1.52	1
					nA_2402	LNWLTKSGS	LNWLYKSEC	LNWL*KS**	'000010011'	0.06	-1.27	1
					nA_2403	LNWLTKSGS	LNWLYKSEC	LNWL*KS**	'000010011'	-1.49	-2.74	1
					nB_1501	LNWLTKSGS	LNWLYKSEC	LNWL*KS**	'000010011'	-1.3	-2.56	1
					nB_1801	LNWLTKSGS	LNWLYKSEC	LNWL*KS**	'000010011'	0.1	-1.02	1
					nB_4001	LNWLTKSGS	LNWLYKSEC	LNWL*KS**	'000010011'	0.64	-1.5	1
					nB_5101	LNWLTKSGS	LNWLYKSEC	LNWL*KS**	'000010011'	-0.11	-1.5	1
				168	nA_1101	NWLTKSGST	NWLYKSECT	NWL*KS**T	'000100110'	-0.31	-2.36	1
					nA_3002	NWLTKSGST	NWLYKSECT	NWL*KS**T	'000100110'	1.31	-1.07	1
					nB_5801	NWLTKSGST	NWLYKSECT	NWL*KS**T	'000100110'	-0.42	-1.54	1
				169	nB_0702	WLTKSGSTY	WLYKSECTY	WL*KS**TY	'001001100'	0.2	-1.54	1
				170	nB_4002	LTKSGSTYP	LYKSECTYP	L*KS**TYP	'010011000'	0	-1.18	1
				171	nA_0203	TKSGSTYPV	YKSECTYPV	*KS**TYPV	'100110000'	0.19	-1.5	1
					nB_4403	TKSGSTYPV	YKSECTYPV	*KS**TYPV	'100110000'	-1.51	-2.86	1
					nB_4501	TKSGSTYPV	YKSECTYPV	*KS**TYPV	'100110000'	-0.26	-1.59	1
				172	nA_2601	KSGSTYPVL	KSECTYPVQ	KS**TYPV*	'001100001'	-0.57	-2.54	1
					nB_0801	KSGSTYPVL	KSECTYPVQ	KS**TYPV*	'001100001'	0.22	-3.22	1
				173	nB_0702	SGSTYPVLN	SECTYPVQN	S**TYPV*N	'011000010'	0.37	-1.92	1
					nB_2705	SGSTYPVLN	SECTYPVQN	S**TYPV*N	'011000010'	-0.01	-2.2	1
					nB_3501	SGSTYPVLN	SECTYPVQN	S**TYPV*N	'011000010'	0.17	-1.4	1
					nB_5101	SGSTYPVLN	SECTYPVQN	S**TYPV*N	'011000010'	-0.67	-1.85	1
					nB_5401	SGSTYPVLN	SECTYPVQN	S**TYPV*N	'011000010'	0.75	-1.42	1
Retained	Affinity Increase	1968_HK68	1972_EN72	166	nA_3301	RLNWLTKSG	RLNWLYKSE	RLNWL*KS*	'000001001'	-1.85	-1.92	1
				167	nA_2301	LNWLTKSGS	LNWLYKSEC	LNWL*KS**	'000010011'	-1.36	-1.67	1
					nA_2403	LNWLTKSGS	LNWLYKSEC	LNWL*KS**	'000010011'	-1.49	-2.74	1
					nB_1501	LNWLTKSGS	LNWLYKSEC	LNWL*KS**	'000010011'	-1.3	-2.56	1

Classification	Change	Yr Old Cluster	Yr New Cluster	pos	Allele	Old Peptide	New Peptide	Delta Peptide	Delta Pattern	Old Sigma	New Sigma	N
				168	nA_2403	NWLTKSGST	NWLYKSECT	NWL*KS**T	'000100110'	-1.62	-1.94	1
				169	nA_0101	WLTKSGSTY	WLYKSECTY	WL*KS**TY	'001001100'	-1.62	-1.89	1
					nA_2601	WLTKSGSTY	WLYKSECTY	WL*KS**TY	'001001100'	-2.13	-2.34	1
					nA_2902	WLTKSGSTY	WLYKSECTY	WL*KS**TY	'001001100'	-2.74	-3.25	1
					nB_5801	WLTKSGSTY	WLYKSECTY	WL*KS**TY	'001001100'	-1.86	-2.23	1
				170	nA_3001	LTKSGSTYP	LYKSECTYP	L*KS**TYP	'010011000'	-1.3	-1.91	1
				171	nB_4002	TKSGSTYPV	YKSECTYPV	*KS**TYPV	'100110000'	-1.32	-2.2	1
					nB_4403	TKSGSTYPV	YKSECTYPV	*KS**TYPV	'100110000'	-1.51	-2.86	1
				172	nA_0203	KSGSTYPVL	KSECTYPVQ	KS**TYPV*	'001100001'	-1.64	-1.95	1
					nA_2301	KSGSTYPVL	KSECTYPVQ	KS**TYPV*	'001100001'	-1.24	-1.33	1
					nA_2402	KSGSTYPVL	KSECTYPVQ	KS**TYPV*	'001100001'	-1.14	-1.32	1
				173	nB_5701	SGSTYPVLN	SECTYPVQN	S**TYPV*N	'011000010'	-1.01	-1.2	1
				174	nA_0101	GSTYPVLNV	ECTYPVQNV	**TYPV*NV	'110000100'	-1.47	-2.1	1
	Affinity Loss	1968_HK68	1972_EN72	166	nA_0301	RLNWLTKSG	RLNWLYKSE	RLNWL*KS*	'000001001'	-2.1	-1.18	1
				167	nA_3101	LNWLTKSGS	LNWLYKSEC	LNWL*KS**	'000010011'	-1.66	-1.61	1
				168	nA_3301	NWLTKSGST	NWLYKSECT	NWL*KS**T	'000100110'	-2.03	-1.82	1
					nB_5301	NWLTKSGST	NWLYKSECT	NWL*KS**T	'000100110'	-1.82	-1.35	1
				169	nA_0201	WLTKSGSTY	WLYKSECTY	WL*KS**TY	'001001100'	-1.71	-1.59	1
				172	nA_0202	KSGSTYPVL	KSECTYPVQ	KS**TYPV*	'001100001'	-2.02	-1.76	1
					nB_3501	KSGSTYPVL	KSECTYPVQ	KS**TYPV*	'001100001'	-1.98	-1.03	1
				173	nA_3001	SGSTYPVLN	SECTYPVQN	S**TYPV*N	'011000010'	-2.04	-1.81	1
					nB_4002	SGSTYPVLN	SECTYPVQN	S**TYPV*N	'011000010'	-1.75	-1.22	1
				174	nA_6901	GSTYPVLNV	ECTYPVQNV	**TYPV*NV	'110000100'	-2.36	-1.5	1
					nB_0801	GSTYPVLNV	ECTYPVQNV	**TYPV*NV	'110000100'	-1.69	-1.27	1
					nB_4001	GSTYPVLNV	ECTYPVQNV	**TYPV*NV	'110000100'	-2.72	-1.25	1

76675 rows have been excluded.

MHC II (Highly Mutable Region) HK68-> EN72 Transition

Classification	Change	Yr Old Cluster	Yr New Cluster	Allele	pos	Old Peptide	New Peptide	Delta Peptide	Delta Pattern	Old Sigma	New Sigma	N
Lost	Affinity Loss	1968_HK68	1972_EN72	nDRB1_0802	166	RLNWLTKSGSTYPVL	RLNWLYKSECTYPVQ	RLNWL*KS**TYPV*	'000001001100001'	-1.26	0.23	1
				nDRB1_1501	173	SGSTYPVLNVTMPNN	SECTYPVQNVTMPNN	S**TYPV*NVTMPNN	'011000010000000'	-1.1	0.94	1
New	Affinity Increase	1968_HK68	1972_EN72	nDRB1_0101	167	LNWLTKSGSTYPVLN	LNWLYKSECTYPVQN	LNWL*KS**TYPV*N	'000010011000010'	0.05	-1.02	1
					169	WLTKSGSTYPVLNVT	WLYKSECTYPVQNVT	WL*KS**TYPV*NVT	'001001100001000'	0.39	-1.23	1
				nDRB1_0301	167	LNWLTKSGSTYPVLN	LNWLYKSECTYPVQN	LNWL*KS**TYPV*N	'000010011000010'	-1.61	-2.69	1
				nDRB1_0401	167	LNWLTKSGSTYPVLN	LNWLYKSECTYPVQN	LNWL*KS**TYPV*N	'000010011000010'	-0.41	-1.5	1
					169	WLTKSGSTYPVLNVT	WLYKSECTYPVQNVT	WL*KS**TYPV*NVT	'001001100001000'	-0.15	-2.1	1
					172	KSGSTYPVLNVTMPN	KSECTYPVQNVTMPN	KS**TYPV*NVTMPN	'001100001000000'	1.4	-1.23	1
				nDRB1_0404	172	KSGSTYPVLNVTMPN	KSECTYPVQNVTMPN	KS**TYPV*NVTMPN	'001100001000000'	-0.45	-1.47	1
				nDRB1_0701	172	KSGSTYPVLNVTMPN	KSECTYPVQNVTMPN	KS**TYPV*NVTMPN	'001100001000000'	0.72	-1.03	1
				nDRB1_0901	172	KSGSTYPVLNVTMPN	KSECTYPVQNVTMPN	KS**TYPV*NVTMPN	'001100001000000'	0.08	-1.29	1
				nDRB3_0101	167	LNWLTKSGSTYPVLN	LNWLYKSECTYPVQN	LNWL*KS**TYPV*N	'000010011000010'	-1.02	-2.87	1
					173	SGSTYPVLNVTMPNN	SECTYPVQNVTMPNN	S**TYPV*NVTMPNN	'011000010000000'	1.14	-1.13	1
				nDRB5_0101	171	TKSGSTYPVLNVTMP	YKSECTYPVQNVTMP	*KS**TYPV*NVTMP	'100110000100000'	0.26	-1.39	1
Retained	Affinity Increase	1968_HK68	1972_EN72	nDRB1_0301	167	LNWLTKSGSTYPVLN	LNWLYKSECTYPVQN	LNWL*KS**TYPV*N	'000010011000010'	-1.61	-2.69	1
				nDRB1_0401	168	NWLTKSGSTYPVLNV	NWLYKSECTYPVQNV	NWL*KS**TYPV*NV	'000100110000100'	-1.46	-1.49	1
				nDRB1_1101	166	RLNWLTKSGSTYPVL	RLNWLYKSECTYPVQ	RLNWL*KS**TYPV*	'000001001100001'	-1.23	-1.81	1
				nDRB1_1302	168	NWLTKSGSTYPVLNV	NWLYKSECTYPVQNV	NWL*KS**TYPV*NV	'000100110000100'	-1.01	-1.21	1
				nDRB3_0101	167	LNWLTKSGSTYPVLN	LNWLYKSECTYPVQN	LNWL*KS**TYPV*N	'000010011000010'	-1.02	-2.87	1
	Affinity Loss	1968_HK68	1972_EN72	nDRB1_0301	166	RLNWLTKSGSTYPVL	RLNWLYKSECTYPVQ	RLNWL*KS**TYPV*	'000001001100001'	-1.63	-1.2	1
					168	NWLTKSGSTYPVLNV	NWLYKSECTYPVQNV	NWL*KS**TYPV*NV	'000100110000100'	-1.88	-1.47	1
				nDRB1_0401	166	RLNWLTKSGSTYPVL	RLNWLYKSECTYPVQ	RLNWL*KS**TYPV*	'000001001100001'	-3.58	-1.86	1
				nDRB1_0701	166	RLNWLTKSGSTYPVL	RLNWLYKSECTYPVQ	RLNWL*KS**TYPV*	'000001001100001'	-1.76	-1.22	1
				nDRB3_0101	166	RLNWLTKSGSTYPVL	RLNWLYKSECTYPVQ	RLNWL*KS**TYPV*	'000001001100001'	-2.35	-1.08	1

29215 rows have been excluded.
